# Divergent histopathological and molecular patterns in chemically induced interstitial cystitis/bladder pain syndrome rat models

**DOI:** 10.1038/s41598-024-67162-y

**Published:** 2024-07-12

**Authors:** Ya-Chuan Chang, Chia-Ying Yu, Chen Dong, Sung-Lang Chen, Wen-Wei Sung

**Affiliations:** 1 Department of Urology, Chung Shan Medical University Hospital, Taichung, 40201 Taiwan; 2 School of Medicine, Chung Shan Medical University, Taichung, 40201 Taiwan; 3 Institute of Medicine, Chung Shan Medical University, Taichung, 40201 Taiwan

**Keywords:** Interstitial cystitis, Bladder pain syndrome, Next-generation sequencing, RNA sequencing, Kyoto encyclopedia of genes and genomes, Medical research, Pathogenesis, Urology

## Abstract

Interstitial cystitis/bladder pain syndrome (IC/BPS) is a complex chronic pain disorder with an elusive etiology and nonspecific symptoms. Although numerous animal models with phenotypes similar to human disease have been established, no available regimen can consistently alleviate clinical symptoms. This dilemma led us to question whether current animal models adequately represent IC/BPS. We compared four commonly used IC/BPS rat models to determine their diverse histopathological and molecular patterns. Female rats were given single treatments with hydrochloric acid (HCL), acetic acid (AA), protamine sulfate plus lipopolysaccharide (PS + LPS), or cyclophosphamide (CYP) to induce IC/BPS. Bladder sections were stained for histopathologic evaluation, and mRNA expression profiles were examined using next-generation sequencing and gene set analyses. Mast cell counts were significantly higher in the HCL and AA groups than in the PS + LPS, CYP, and control groups, but only the AA group showed significant collagen accumulation. The models differed substantially in terms of their gene ontology and Kyoto encyclopedia of genes and genomes pathways. Our observations suggest that none of these rat models fully reflects the complexity of IC/BPS. We recommend that future studies apply and compare multiple models simultaneously to fully replicate the complicated features of IC/BPS.

## Introduction

Interstitial cystitis/bladder pain syndrome (IC/BPS) is a complex chronic pain disorder that affects the urinary bladder and is characterized by symptoms such as urinary frequency, urgency, and pain^1^. The prevalence of IC/BPS is higher in women (45–300/100,000) than in men (8–30/100,000). Due to the diverse and nonspecific nature of IC/BPS symptoms, various terminologies have been used in clinical settings to describe the condition^2^. Moreover, identifying accurate biomarkers for IC/BPS diagnosis, such as urinary and serum nerve growth factors, pro-inflammatory cytokines, and pro-nociceptive inflammatory receptors, remains challenging in clinical practice. Furthermore, the diagnosis of IC/BPS primarily relies on clinical symptoms and the exclusion of other potential causes, such as vesical stones, bladder cancer, and urethral diverticula^3,4^. Consequently, the absence of standardized diagnostic procedures and the use of ambiguous diagnostic terminology in clinical practice may lead to underreporting of the condition’s prevalence. The 2022 American Urological Association guidelines categorize treatment options for IC/BPS into behavioral/non-pharmacologic remedies, oral medications, bladder instillations and procedures, and major surgeries. These treatment plans are individually tailored to address the unique and varied characteristics of each patient’s condition^5^, since the lack of a comprehensive understanding of IC/BPS pathophysiology and its nonspecific symptoms hinders the establishment of standardized diagnostic procedures. In practice, surgical interventions for IC/BPS are associated with relatively low-quality outcomes, with a clinical symptom improvement failure rate of approximately 23%^6^.

The etiology of IC/BPS remains elusive, and several related hypotheses have been proposed, including neurogenic inflammation via mast cell infiltration, increased barrier permeability due to bladder epithelium injury, and potential autoimmune involvement^7^. However, due to the limited understanding of IC/BPS pathophysiology, it has proven challenging to develop an appropriate animal model that fully replicates the symptoms of this condition. Currently, more than 20 existing animal models with phenotypes resembling human IC/BPS have been used with various chemical agents, but no standard animal model has yet been established. Among the chemical toxiins used, cyclophosphamide (CYP) is the predominant choice for inducing IC/BPS, followed by hydrochloric acid (HCL), protamine sulfate (PS), lipopolysaccharide (LPS), or a combination of multiple toxins^8^. All these models have demonstrated mechanisms that impair bladder function^9^. CYP is metabolized into acrolein in the liver, which is a highly reactive aldehyde that is subsequently excreted into the bladder through the kidneys. As acrolein accumulates in the bladder, it interacts with the umbrella cells of the luminal urothelium, inducing an inflammatory response^9^. These chemical agents are preferred due to their ability to directly damage the urothelium and other bladder cells, leading to mucosa erosion, hemorrhage, edema, and leukocytic infiltration of the bladder wall, which closely resembles the pathology seen in human IC/BPS cases. Using such animal models provides better control over the timing, duration, and severity of inflammation^10^. Although some studies have explored more complex IC/BPS models, including autoimmune and stress-induced models^8^, they are less frequently employed in research compared to chemically induced models.

Despite the establishment of numerous animal IC/BPS models to assist in the selection of clinical treatment regimens, no single or combined treatment regimen has been developed that can consistently alleviate IC/BPS symptoms and ensure long-term treatment efficacy^7^. This has led to concerns among researchers regarding potentially underrepresented or unknown variables in existing models. In the present study, the researchers conducted a comprehensive comparison of the most frequently employed IC/BPS animal models to examine their histopathological and molecular characteristics, assess the representativeness of the current models, and evaluate the credibility of existing research.

## Methods

### Animal model

The animal use protocol was approved by the Chung-Shan Medical University Experimental Animal Center (No. 2098), and all experiments were performed in accordance with relevant guidelines and regulations. All methods are reported in accordance with ARRIVE guidelines. We established four distinct IC animal models using 7-weeks-old female Sprague–Dawley rats weighing 200–230 g (source: BioLASCO Taiwan Co., Ltd). The rats were anesthetized with Rompun 6 mg/kg and Zoletil 30 mg/kg administered intraperitoneally. The control rats (n = 4) received no treatment. For the experimental groups, a sterile polyethylene catheter (PE-50) was inserted into the bladder through the urethra for intravesical instillation (IVI). The HCL group (n = 3) underwent IVI with 0.4 M HCL for 4 min. The AA group (n = 4) was subjected to IVI with 3% AA for 30 min. The PS + LPS group (n = 4) received IVI with a combination of 10 mg/ml PS and 1.5 mg/ml LPS for 30 min. All IVI procedures were carried out during the specified sedation period. The CYP group (n = 4) received 150 mg/kg of CYP intraperitoneally. All treated rats underwent indicative interventions once to induce IC symptoms and were sacrificed 14 days after induction according to previous study^11^. The bladder is longitudinally bisected, with one half immersed in formalin for pathological evaluation and the other half preserved in RNA keeper for molecular study.

### Histopathological study

The tissue sections were fixed in formalin solution and then dehydrated with alcohol at increasing concentrations, after which they were treated with xylene and placed in paraffin. The paraffin-embedded blocks were then cut into 3-µm sections for further analysis. The tissue sections were stained with hematoxylin and eosin (H&E). To detect collagen deposition and evaluate the intensity of the inflammatory process in the bladder, the researchers used Masson’s trichrome and toluidine blue, respectively. The staining process followed the manufacturer’s protocol. The stained tissue sections were then photographed with TissueFAX Plus (v. 20, https://www.meyerinst.com/brand/tissuegnostics/tissuefaxs-plus/), and the sum intensity of the collagen was analyzed. The number of mast cells per mm^2^ were counted by the same experienced researcher (n = 3). Statistical analysis was conducted using SPSS (v. 20.0, https://www.ibm.com/support/pages/spss-statistics-20-available-download) based on mean ± SD. There is no significant difference between bars with the same letters, and there is a significant statistical difference between bars with different letters determined using one-way ANOVA and a post-hoc test with Tukey’s HSD (honest significant difference) test^12,13^.

### RNA preparation

Bladder tissue total RNA from interstitial cystitis rat models was extracted using Trizol™ Reagent (Invitrogen, USA) according to the instructions. Purified RNA was quantified at OD 260 nm using a Nanodrop® ND-1000 spectrophotometer (Thermo Fisher Scientific, USA) and qualitated using a 2100 Bioanalyzer (Agilent Technologies, USA) with an RNA 6000 LabChip nano assay kit (Agilent Technologies, USA). All RNA sample preparation procedures (n = 3) were conducted according to the protocol provided by Illumina (USA). For mRNA sequencing, libraries were constructed using the Agilent Technologies’ SureSelect Strand-Specific RNA library preparation kit, followed by size selection with AMPure XP bead-based reagent (Beckman Coulter, USA). Following library preparation, the sequencing process was executed using Illumina’s sequencing-by-synthesis (SBS) technology. The acquisition of sequencing data in the form of FASTQ reads was facilitated by leveraging Welgene Biotech’s pipeline (Taipei, Taiwan), which is based on Illumina’s base-calling bcl2fastq program (v. 2.20, https://emea.support.illumina.com/sequencing/sequencing_software/bcl2fastq-conversion-software.html). To eliminate subpar quality reads or bases, a procedure known as sequence quality trimming was conducted using Trimmomatic (v. 0.36, http://www.usadellab.org/cms/?page=trimmomatic). The HISAT2 software tool (v. 2.2.1, https://www.ccb.jhu.edu/software/hisat/index.shtml) was employed for mRNA alignment because it is renowned for its rapid and sensitive alignment capabilities, which effectively facilitate the mapping of next-generation sequencing reads onto genomic references^14^.

### Gene ontology (GO) and Kyoto encyclopedia of genes and genomes (KEGG) pathway analyses

To evaluate the overrepresented GO of molecular function and biological processes, KEGG pathway analyses were performed using the compareCluster function of the clusterProfiler R package (v. 3.6, https://guangchuangyu.github.io/software/clusterProfiler/)^15–17^. Differential expression analysis was conducted employing cuffdiff (Cufflinks v. 2.2.1, https://github.com/cole-trapnell-lab/cufflinks) while incorporating genome bias detection/correction methodologies^18^ and Welgene Biotech’s internally developed pipeline. Functional enrichment analysis of differentially expressed genes for each experimental design was conducted using clusterProfiler(v. 3.6, https://guangchuangyu.github.io/software/clusterProfiler/)^19^. Genes exhibiting statistical significance were indicated by *p*-values ≤ 0.05, and fold changes ≥ 2 were identified as significantly differentially expressed. Furthermore, genes characterized by low expression levels (with FPKM values < 0.3) in either or both the treated and control samples were excluded from the analysis.

## Results

**Figure 1:**
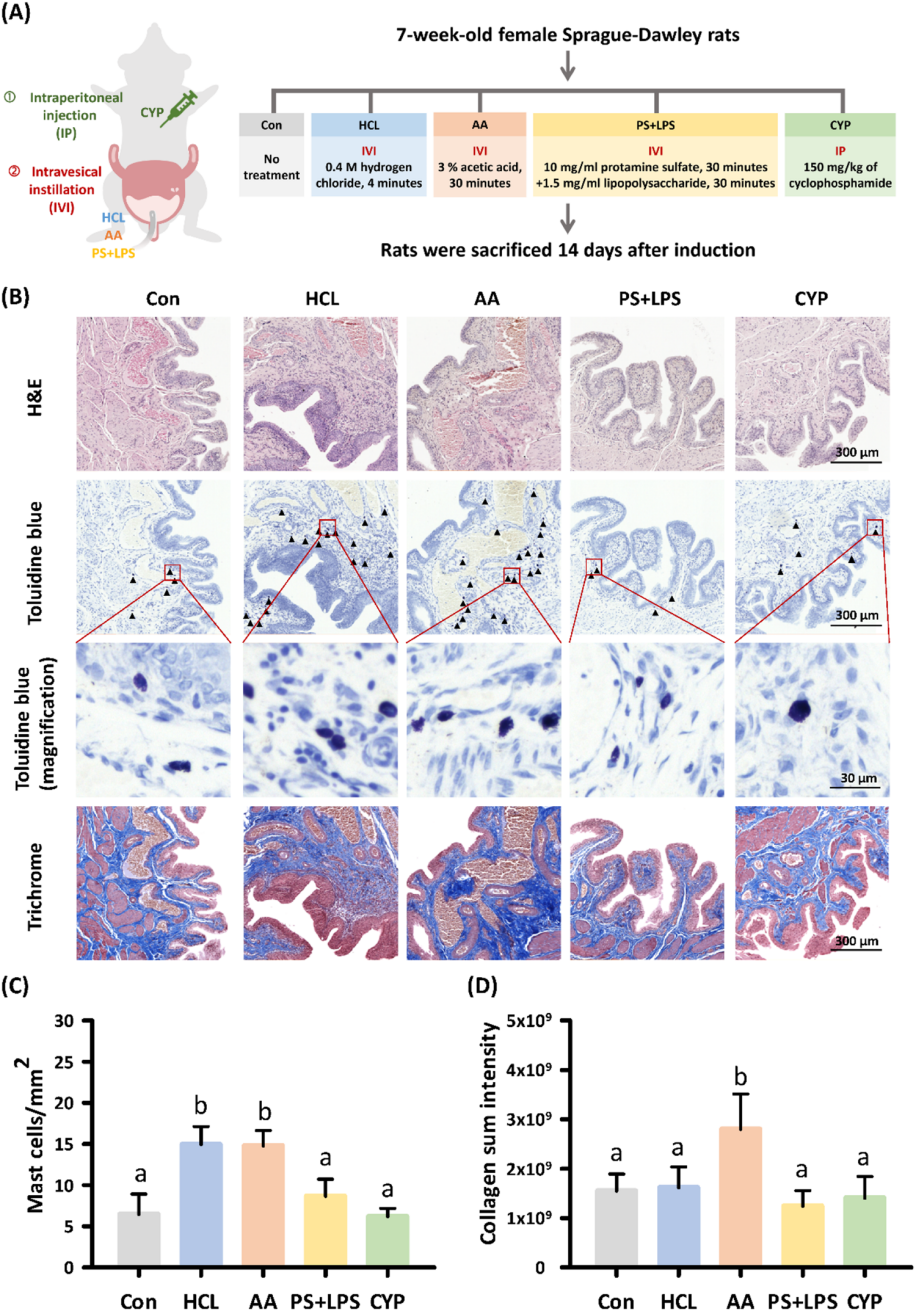
Bladder tissue pathological assessment and quantification in interstitial cystitis rat models. (**A**) Flowchart showing the experimental design of the animal studies. (**B**) Histologic features of interstitial cystitis induction in bladder tissue were observed using H&E staining, and the infiltrated mast cells distributed in bladder tissues were stained with toluidine blue (arrows). Characterization of rat bladder sections using Masson’s trichrome staining; images show collagen with blue staining and muscle with purple staining. (**C**) The number of mast cells per mm^2^ in the bladder epithelium and lamina propria (n = 3). (**D**) The sum intensity of collagen in the bladder epithelium and lamina propria was determined based on the images stained with Masson’s trichrome taken using TissueFAX Plus software (n = 3–4). Bars depict the means ± SDs. There is no significant difference between bars with the same letters, and there is a significant statistical difference between bars with different letters determined using one-way ANOVA and a post-hoc test with Tukey’s HSD (honest significant difference) test.

The similarities and differences in histopathological patterns between the four IC/BPS models were evaluated by analyzing stained bladder sections using the animal model flowchart shown in Fig. 1A. The histological manifestations revealed by H&E staining, compared to the controls, indicated significant thickening within the bladder epithelium in the HCL-induced model, whereas no significant thickness changes were noted for the other groups (Fig. 1B). Toluidine blue staining revealed that both the HCL-induced and AA-induced models showed significantly increased mast cell infiltration compared to the controls, which aligns with previous studies that identified mast cell infiltration as a hallmark of IC/BPS. However, no significant changes in mast cell infiltration were observed in the PS + LPS-induced and CYP-induced models compared to the controls. Interestingly, significant differences were noted in the mast cell infiltration of the HCL, AA, PS + LPS, and CYP groups (Fig. 1C). Trichrome staining, performed to detect fibrosis, revealed that only the AA-induced model showed collagen deposition/fibrotic tissue in the bladder epithelium and lamina propria, with significant differences in the collagen deposition/fibrotic tissue compared to the other groups (Fig. 1D). Overall, the four IC/BPS models showed few similarities in their histopathological patterns.

**Figure 2:**
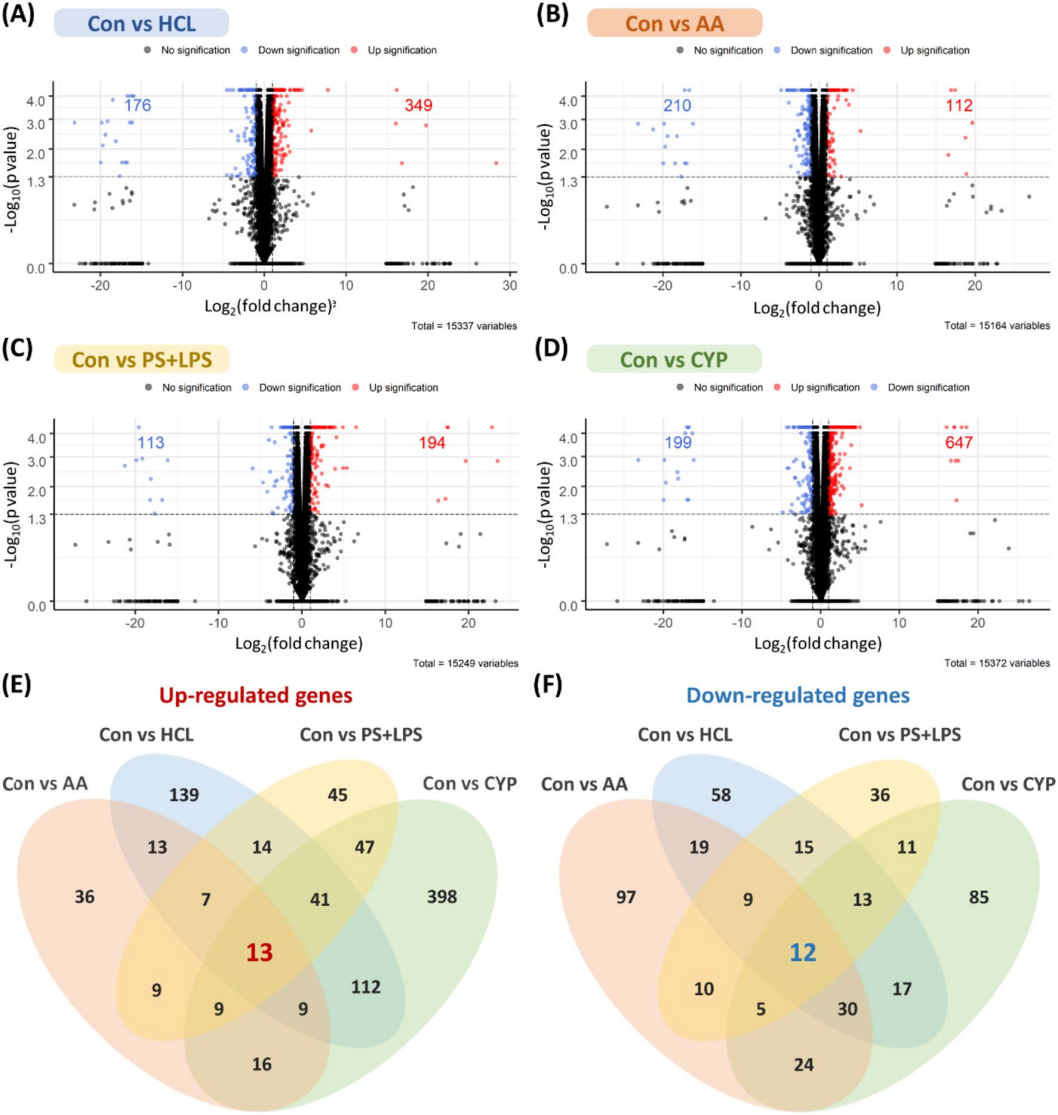
Differentially expressed genes in four interstitial cystitis rat models. Volcano plots of upregulated and downregulated differentially expressed genes in (**A**) HCL, (**B**) AA, (**C**) PS + LPS, and (**D**) CYP models, respectively (n = 3). The x-axis represents a relative expression fold change of Log_2_ fold compared to the controls, and the y-axis indicates the threshold of a − Log_10_ *p* value. The red and blue points in the top right and top left sections show significantly upregulated and downregulated genes, respectively. Common (**E**) upregulated and (**F**) downregulated differentially expressed genes in four interstitial cystitis rat models.

The RNA sequencing analysis of bladder tissue from various animal models compared to the controls identified common upregulated or downregulated genes. In the HCL-, AA-, PS + LPS-, and CYP-induced models, there were 349, 112, 194, and 647 significantly upregulated genes, respectively, and 176, 210, 113, and 199 significantly downregulated genes, respectively (Fig. 2A–D). Table 1 summarizes the top 10 genes based on fold changes with statistical significance and known molecular functions. The complete lists are provided in Supplementary Tables 1–8. Comparing these models, the HCL, AA, and PS + LPS models showed 7 common upregulated and 9 downregulated genes, while the CYP, PS + LPS, and HCL models exhibited 41 common upregulated and 13 downregulated genes. Furthermore, the AA, CYP, and PS + LPS models shared 9 upregulated and 30 downregulated genes, and the PS + LPS, CYP, and AA models had 9 upregulated and 5 downregulated genes in common. Collectively, all four models shared 13 upregulated and 12 downregulated genes compared to the controls (Fig. 2E,F).

**Table 1:** Lists of selected genes with the top 10 fold changes compared to the control group

	Con versus HCL				Con versus AA			
	Gene id	Gene name	Log_2_(fold change)	*p* value	Gene id	Gene name	Log_2_(fold change)	*p* value
Top 10 up-regulated	ENSRNOG00000051481	SCARNA18	28.323	0.02395	ENSRNOG00000056555	AABR07054983.1	19.646	0.00135
	ENSRNOG00000032130	LOC691427	19.766	0.0017	ENSRNOG00000059770	RT1-CE16	18.868	0.04285
	ENSRNOG00000050257	AABR07003273.1	16.812	0.02395	ENSRNOG00000056094	AC125873.1	18.756	0.0044
	ENSRNOG00000060174	AABR07070775.1	16.163	0.00005	ENSRNOG00000014118	Klkb1	17.423	0.00005
	ENSRNOG00000022724	Cnih3	16.070	0.00145	ENSRNOG00000051360	RGD1563270	16.903	0.00005
	ENSRNOG00000047706	RT1-CE16	7.778	0.00005	ENSRNOG00000047971	LOC100910528	16.553	0.01435
	ENSRNOG00000046414	LOC108348048	5.722	0.0026	ENSRNOG00000046414	LOC108348048	5.323	0.00265
	ENSRNOG00000045569	Nol8	4.615	0.00005	ENSRNOG00000059348	Ube2b	4.313	0.00005
	ENSRNOG00000050407	NEWGENE_1305281	4.311	0.00005	ENSRNOG00000045655	Tbcb	3.839	0.00025
	ENSRNOG00000009508	Casp6	4.123	0.00005	ENSRNOG00000060898	LOC100910418	3.619	0.00005
Top 10 down-regulated	ENSRNOG00000035103	5S_rRNA	− 23.188	0.00135	ENSRNOG00000035103	5S_rRNA	− 23.188	0.00145
	ENSRNOG00000054722	U1	− 19.970	0.02285	ENSRNOG00000048176	AABR07024593.2	− 21.260	0.00235
	ENSRNOG00000048073	LOC103690821	− 19.868	0.00135	ENSRNOG00000054722	U1	− 19.970	0.02395
	ENSRNOG00000055311	U1	− 19.700	0.0077	ENSRNOG00000048073	LOC103690821	− 19.868	0.00145
	ENSRNOG00000051920	U1	− 19.409	0.0036	ENSRNOG00000055311	U1	− 19.700	0.0083
	ENSRNOG00000043400	LOC100910678	− 19.144	0.0012	ENSRNOG00000051920	U1	− 19.409	0.00395
	ENSRNOG00000059703	AC123425.1	− 18.500	0.00015	ENSRNOG00000032847	RGD1561636	− 18.434	0.02395
	ENSRNOG00000052706	AABR07072841.1	− 18.132	0.0058	ENSRNOG00000053409	AABR07068852.1	− 18.222	0.00395
	ENSRNOG00000028311	Cnpy1	− 17.673	0.0481	ENSRNOG00000028311	Cnpy1	− 17.673	0.04905
	ENSRNOG00000043480	Timm8a1	− 17.365	0.02285	ENSRNOG00000050226	RGD1565894	− 17.224	0.00005

	Con versus PC + LPS				Con versus CYP			
	Gene id	Gene name	Log_2_(fold change)	*p* value	Gene id	Gene name	Log_2_(fold change)	*p* value
Top 10 up-regulated	ENSRNOG00000055067	5S_rRNA	23.473	0.00145	ENSRNOG00000050567	LOC100911674	18.560	0.00005
	ENSRNOG00000060522	Metazoa_SRP	22.800	0.00005	ENSRNOG00000011557	S100a8	17.927	0.00005
	ENSRNOG00000051831	5_8S_rRNA	19.662	0.00145	ENSRNOG00000050257	AABR07003273.1	17.493	0.00145
	ENSRNOG00000011557	S100a8	17.488	0.00005	ENSRNOG00000049123	AABR07066510.1	17.281	0.02365
	ENSRNOG00000060292	NEWGENE_1587253	17.463	0.00005	ENSRNOG00000051891	RGD1562378	17.166	0.00145
	ENSRNOG00000049123	AABR07066510.1	17.228	0.02135	ENSRNOG00000060181	AABR07050321.2	17.003	0.00005
	ENSRNOG00000054804	AABR07017783.3	16.361	0.02395	ENSRNOG00000028114	Hbq1b	16.986	0.00005
	ENSRNOG00000047706	RT1-CE16	6.487	0.00005	ENSRNOG00000016278	Ccl17	16.555	0.00145
	ENSRNOG00000046414	LOC108348048	5.369	0.0026	ENSRNOG00000026133	Ly6d	16.028	0.00005
	ENSRNOG00000054959	Mmp11	4.934	0.0026	ENSRNOG00000016361	Plcd4	5.201	0.03105
Top 10 down-regulated	ENSRNOG00000048176	AABR07024593.2	− 21.260	0.00215	ENSRNOG00000035103	5S_rRNA	− 23.188	0.00135
	ENSRNOG00000048073	LOC103690821	− 19.868	0.00135	ENSRNOG00000054722	U1	− 19.970	0.02285
	ENSRNOG00000053109	Mrpl53	− 19.564	0.00005	ENSRNOG00000048073	LOC103690821	− 19.868	0.00135
	ENSRNOG00000043400	LOC100910678	− 19.144	0.0012	ENSRNOG00000055311	U1	− 19.700	0.0077
	ENSRNOG00000057273	AABR07062157.1	− 18.213	0.02285	ENSRNOG00000053409	AABR07068852.1	− 18.222	0.0036
	ENSRNOG00000052706	AABR07072841.1	− 18.132	0.0058	ENSRNOG00000052706	AABR07072841.1	− 18.132	0.0058
	ENSRNOG00000028311	Cnpy1	− 17.673	0.0481	ENSRNOG00000050226	RGD1565894	− 17.224	0.0001
	ENSRNOG00000057895	AABR07033745.1	− 16.764	0.02285	ENSRNOG00000028803	Cd209e	− 17.027	0.00005
	ENSRNOG00000058218	AC131806.3	− 16.104	0.00135	ENSRNOG00000061507	AABR07032328.1	− 16.982	0.02285
	ENSRNOG00000045790	LOC100910130	− 5.932	0.00265	ENSRNOG00000046991	RGD1565472	− 16.861	0.00005

**Figure 3:**
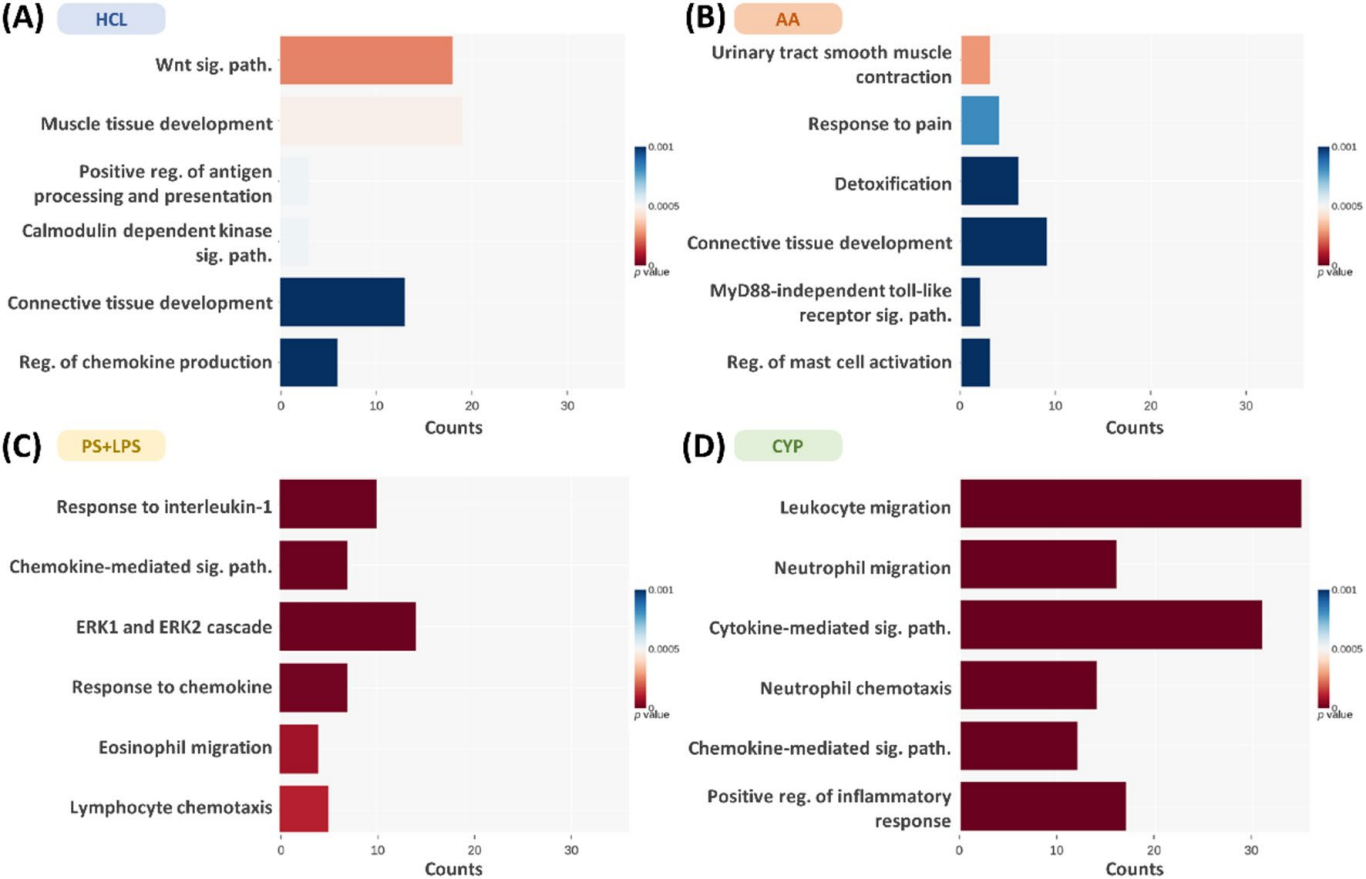
The top five enriched biological processes of the differentially expressed genes in the four interstitial cystitis rat models. RNA sequencing data revealed distinct alterations in biological process (BP) gene sets in the GO database for (**A**) HCL, (**B**) AA, (**C**) PS + LPS, and (**D**) CYP models, respectively (n = 3). The x-axis indicates the number of genes annotated under the indicated pathway, and the y-axis indicates the names of the KEGG pathways. The bar color indicates the *p* value for each term.

Analyzing the RNA sequencing data revealed distinct alterations in biological process (BP) and molecular function (MF) gene sets in the GO database for each IC/BPS model. The common biological process gene sets among the four animal models were only for connective tissue development (HCL and AA) and chemokine-mediated signaling pathways (PS + LPS and CYP). Notable changes in biological processes were observed in specific models. The HCL model showed alterations in Wnt signaling, muscle tissue development, antigen processing regulation, calmodulin-dependent kinase signaling, and chemokine production. The AA model exhibited changes in urinary tract muscle contraction, pain response, detoxification, MyD88-independent toll-like receptor (TLR) signaling, and mast cell activation regulation. The PS + LPS model showed alterations in interleukin-1 response, ERK1/ERK2 cascade, chemokine response, eosinophil migration, and lymphocyte chemotaxis. Finally, the CYP model demonstrated changes in leukocyte migration, cytokine signaling, neutrophil activities, and inflammatory response regulation (Fig. 3A–D).

**Figure 4:**
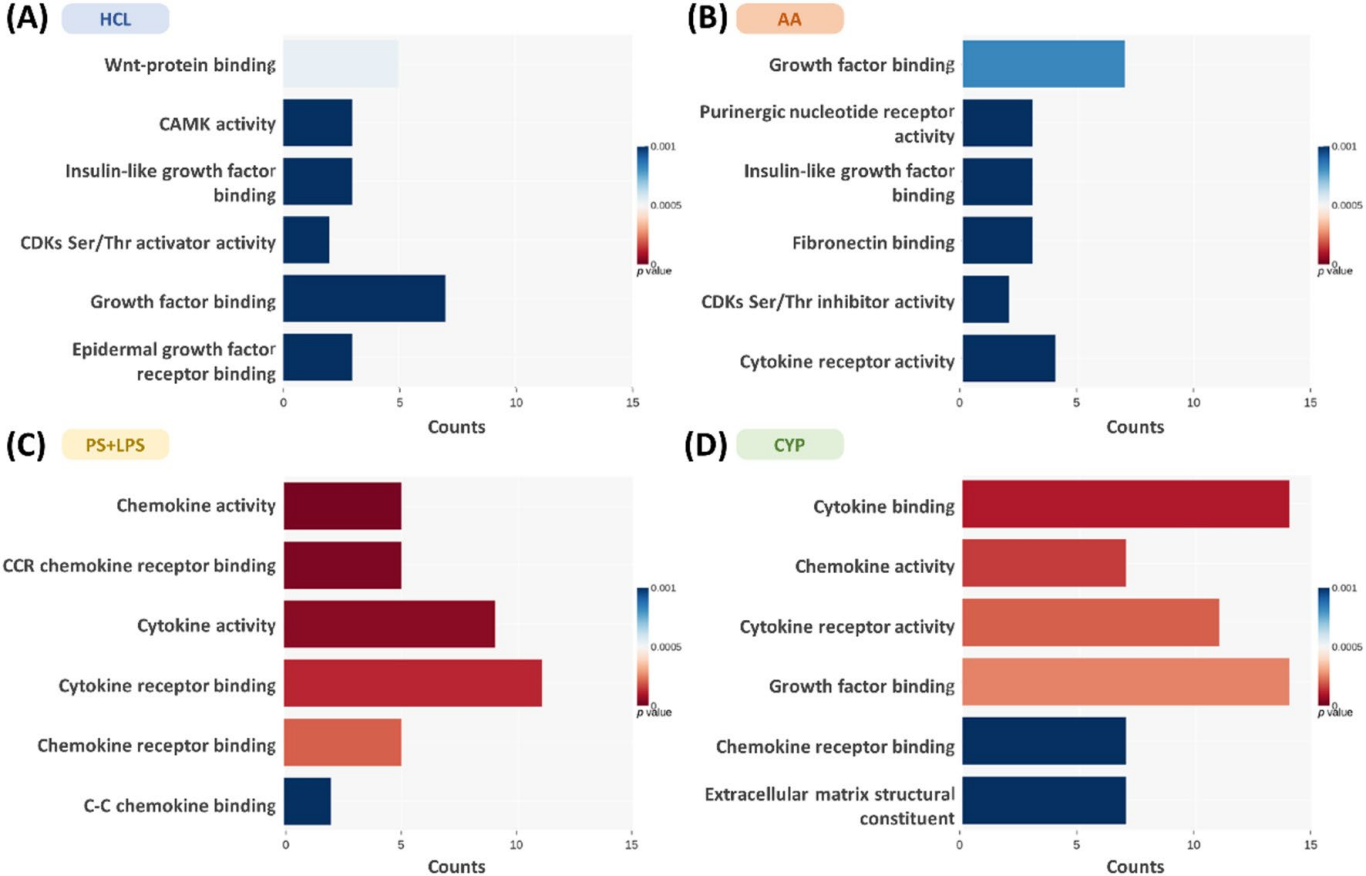
The top five enriched molecular functions of the differentially expressed genes in the four interstitial cystitis rat models. RNA sequencing data revealed distinct alterations in molecular function (MF) gene sets in the GO database for (**A**) HCL, (**B**) AA, (**C**) PS + LPS, and (**D**) CYP models (n = 3). The x-axis indicates the number of genes annotated under the indicated pathway, and the y-axis indicates the names of the KEGG pathways. The bar color indicates the *p* value for each term.

The common alterations in MF gene sets among the four animal models included insulin-like growth factor binding (HCL and AA), growth factor binding (HCL, AA, and CYP), cytokine receptor activity (AA and CYP), chemokine activity (PS + LPS and CYP), and chemokine receptor binding (PS + LPS and CYP). Distinct alterations in molecular functions were also evident. The HCL model showed changes in Wnt-protein binding, CAMK activity, CDK activation, and growth factor receptor binding. The AA model displayed alterations in purinergic receptor activity, fibronectin binding, and CDK inhibition. The PS + LPS model demonstrated changes in chemokine/cytokine receptor binding, cytokine activity, and C–C chemokine binding. The CYP model showed variations in cytokine binding and extracellular matrix structural constituents (Fig. 4A–D).

**Figure 5:**
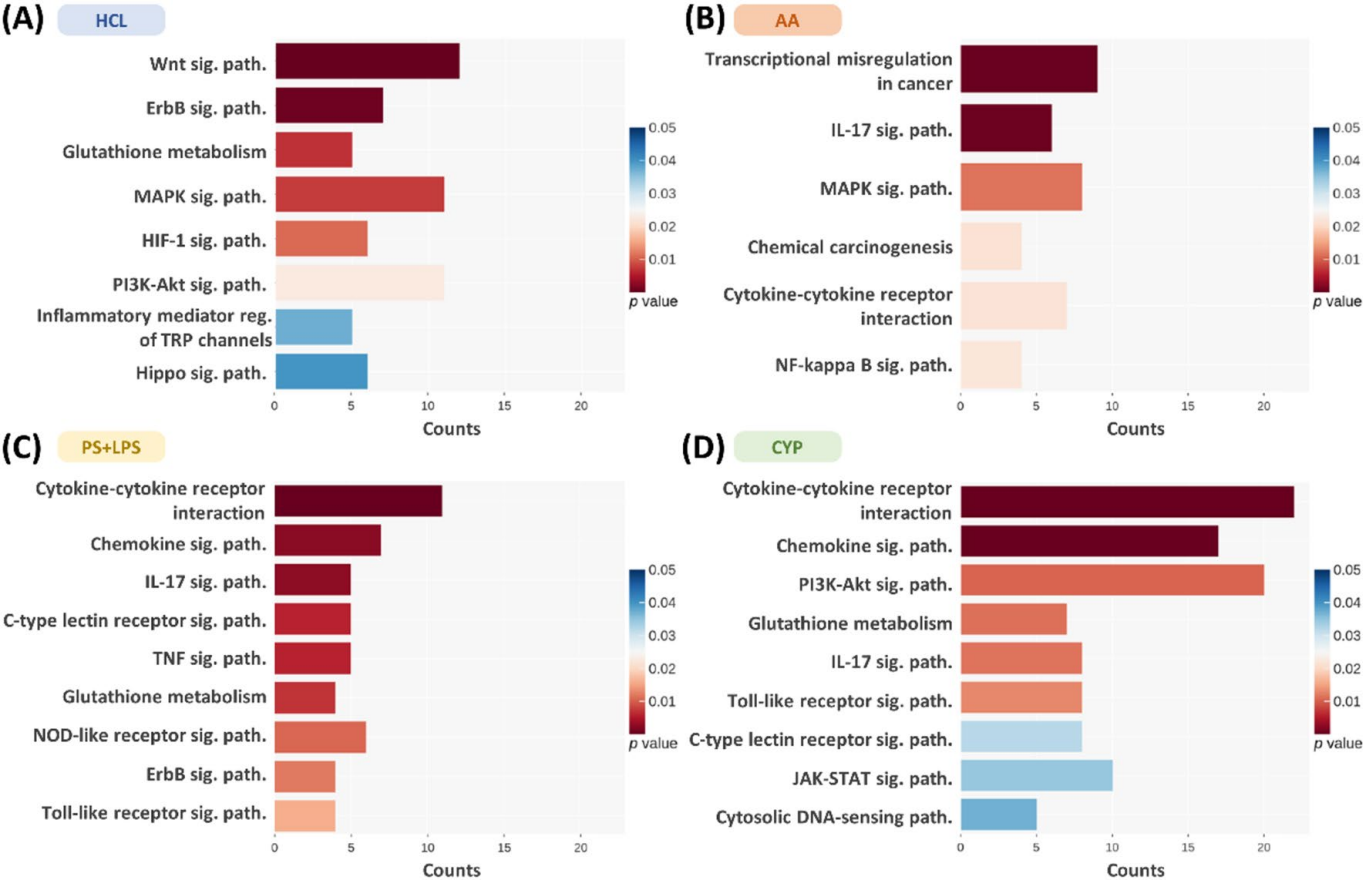
The top 10 significantly enriched Kyoto Encyclopedia of Genes and Genomes (KEGG) pathways of the differentially expressed genes in 4 interstitial cystitis rat models. RNA sequencing data revealed distinct alterations in the KEGG gene sets for (**A**) HCL, (**B**) AA, (**C**) PS + LPS, and (**D**) CYP models (n = 3). The x-axis indicates the number of genes annotated under the indicated pathway, and the y-axis indicates the names of the KEGG pathways. The bar color indicates the *p* value for each term.

The KEGG pathways differed significantly among the models (Fig. 5A–D). Common pathway alterations included ErbB signaling (HCL and PS + LPS), glutathione metabolism (HCL, PS + LPS, and CYP), MAPK signaling (HCL and AA), PI3K-Akt signaling (HCL and CYP), chemokine c-type lectin receptor and TLR signaling (PS + LPS and CYP), and cytokine–cytokine receptor interactions, and IL-17 signaling (AA, PS + LPS, and CYP). Each model exhibited specific pathway changes. The HCL model showed alterations in Wnt signaling, HIF-1 signaling, TRP channels, and hippo signaling. The AA model displayed changes in transcriptional dysregulation in cancer, chemical carcinogenesis, and NF-κB signaling. The PS + LPS model exhibited alterations in the TNF- and Nod-like receptor signaling pathways. Finally, the CYP model demonstrated variations in JAK-STAT and cytosolic DNA-sensing pathways (Fig. 5A–D). Overall, the GO and KEGG pathway analyses highlighted significant differences among the commonly used IC/BPS rat models, indicating the uniqueness of these models.

## Discussion

The current discussion is based on a comparative analysis of histopathological patterns, GO, and KEGG pathways across the four commonly employed IC/BPS rat models and a control group. The analysis revealed notable differences in mast cell counts, with higher counts observed in the HCL and AA groups compared to the PS + LPS, CYP, and control groups, while distinctive collagen accumulation was observed solely in the AA group. Despite these distinct histopathological features, the alterations detected in the GO and KEGG pathways indicate incomplete replication of the multifaceted nature of IC/BPS within these models. These outcomes suggest that future investigations should encompass multiple models to comprehensively grasp the spectrum of IC/BPS manifestations.

In terms of bladder anatomy and physiology, the urothelium is the innermost layer of the bladder, also known as the epithelial or mucosal barrier. It serves a crucial function by providing a robust barrier that prevents urine from seeping into surrounding tissues, thereby averting infection and maintaining a stable internal environment. Below the urothelium, the detrusor muscle, a layer of smooth muscle in the bladder wall, is essential for the bladder’s capacity to store and expel urine. Nerves relay signals to and from the central nervous system to regulate bladder functions, including sensing bladder fullness and initiating contractions. Proper coordination between the nervous system and bladder muscles is vital for voluntary urination control. In discussing bladder anatomy, it is important to mention the trigone area, which is often overlooked. Located at the bladder’s base, the trigone is a triangular region defined by the two ureteral orifices and the urethral orifice. It plays a significant role in the pathophysiology of various bladder diseases due to its unique structure and functions. Unlike other parts of the bladder, the trigone does not significantly expand or contract. In many bladder disorders, especially those affecting the lower urinary tract, the trigone can be a key site for pathological changes. In the context of bladder-centric diseases, the trigone’s unmyelinated nociceptive C-fibers are believed to be upregulated, highlighting its potential as a target for diagnostic techniques and treatments such as local anesthetics and chemodenervation. In women, accessing the trigone is more straightforward through the vaginal route-T3 than via cystoscopy. This simpler approach could be utilized both for diagnostic assessments and for treating acute episodes or long-term management of IC/BPS with botulinum toxin A^20^.

Elucidating the underlying mechanisms of IC/BPS remains challenging despite the use of more than 20 animal models, none of which have been standardized. In terms of histopathological patterns, previous research demonstrated that HCL, AA, and uroplakin (UPK) groups with severe urothelial erosion and CYP and LPS groups had hyperplasia. In addition, toluidine blue staining of the UPK, CYP, HCL, and LPS groups showed increased tissue fibrosis and infiltration of mast cells^11^. The difference in mast cell counts in the CYP group was possibly due to variations in the chemical administration method (IVI method vs. intraperitoneal injection). The difference in the AA group results may require further exploration to achieve confirmation. In summary, we expected to see the occurrence of fibrosis in all four models in response to physiological and pathological stimulation, as well as tissue remodeling/repair and inflammation during subsequent wound healing, but the four IC/BPS models showed few similarities in their histopathological patterns.

Analysis of BP and MF in each IC/BPS model unveiled alterations in genes associated with cytokines, chemokines, or their receptors, aligning with current research findings. One study indicated elevated levels of nerve growth factor, insulin-like growth factor-1, and transforming growth factor- β1 in an inflamed bladder treated with CYP compared to the controls^21^. Additionally, the inflamed bladder of CYP-induced rodents exhibited increased levels of inflammatory cytokines, including TNF-α/β, IL-1α, IL-1β, IL-2, IL-4, IL-5, IL-6, IL-10, and IL-18^22–27^. Following LPS treatment of rodents, a surge in pro-inflammatory cytokines, such as IL-1α, IL-1β, IL-6, IFN, and TNF-α, was observed within 24 h post-treatment^28–34^. Moreover, previous researchers noted elevated levels of IL-6 in bladder tissue after HCL treatment^35^. Limited data are available for the AA group. Studies on various inducers revealed elevations in inflammatory cytokines in rodents, corroborating our observations. However, the underlying mechanisms and cellular interactions require further investigation, given the limited prior data available.

Similarly, significant alterations in the KEGG pathways in our study mostly align with previous research. Prior studies have demonstrated increased expression of Neuregulin 1 and ErbB2 tyrosine kinase in CYP-induced IC/BPS^36^—a phenomenon linked to microglia activation and the potential induction of allodynia^37^. While glutathione metabolism has proven predictive and therapeutic in bladder cancer response to neoadjuvant chemotherapy^38^, its role in IC/BPS remains ambiguous, necessitating further investigation. Moreover, rats treated with PS exhibited a heightened expression of proteins related to the TGF-β/MAPK signaling pathway^39^. Notably, a study using LY294002—a PI3K inhibitor—showed reduced bladder wall thickening in a CYP-induced cystitis rat model, supporting the involvement of PI3K–Akt pathway signaling in IC/BPS models^40^. Although limited data exist on IL-17 signaling pathway alterations in animal models, elevated IL-17A mRNA levels in the biopsies of seven IC/BPS patients have been reported^41^. Another study indicated that IL-17 receptor A might prolong the chronic inflammatory process in IC/BPS while also inducing protective responses, potentially serving as a therapeutic target^42^.

Various cytokine–cytokine receptor interactions^22^ and chemokine signaling pathways^23,43–46^ were elevated in the CYP-induced animal model (a subject already discussed regarding GO analysis). Additionally, prior research has highlighted the role of the Nod-like and C-type lectin receptor pathways in triggering acute CYP-induced cystitis in mice through caspase recruitment domain-containing protein 9 (CARD9) signaling^47,48^. TLRs have also been implicated in IC/BPS pathogenesis; TLR7 was observed to be upregulated in bladder biopsies of Hunner-type IC patients and in TLR7 agonist-induced cystitis in mice^49^. Moreover, TLR4 inflammatory responses within peripheral blood mononuclear cells have been identified as a marker for widespread pain in IC/BPS, necessitating further research to elucidate their specific roles in the pathway^50^. Notably, one study showed the crucial role of TLR4 in triggering bladder dysfunction and inflammation in animal models subjected to CYP treatment^51^. In summary, our results mostly align with previous research in that some of the common KEGG pathways are involved during IC/BPS pathogenesis. Nevertheless, discrepancies emerged in the activation of specific pathways among the four diverse animal models, emphasizing the need for a more nuanced understanding of the pathways involved in each model.

The complicated and poorly understood pathophysiology of IC/BPS creates difficulties in developing therapeutic regimens and establishing standardized animal models. Previous studies have also demonstrated the limitations of each chemical agent used for the induction of IC/BPS in animal models, especially given that each model represents only part of the human IC/BPS phenotype, such as the pathophysiological manifestations, specific protein expressions, and inflammatory responses. The selected agent may also induce other non-IC/BPS characteristics that are not relevant to human disease, including nonspecific damage to the mucosa and the glycosaminoglycan layer of the bladder^52,53^.

Our study outcomes, given the divergent histopathological and molecular patterns, indicate that none of these rat models effectively reflects the complexity of IC/BPS, thereby raising concerns about comparing the results obtained with different IC/BPS models or applying the models interchangeably. These methodological flaws could impair the credibility and value of preclinical studies performed to date. We recommend that future IC/BPS model studies apply and compare multiple models simultaneously to better reflect the complicated features of IC/BPS while bridging the gap between theory and practice.

Several limitations were evident in our study. In our exploratory study, a limited number of animals were used in adherence to the 3Rs: replacement, reduction, and refinement. However, from the standpoint of rigor and reproducibility, this is not sufficient. Future work is necessary to address these limitations. The lack of information on Hunner’s lesions and the inability to perform cystoscopy to confirm the type of IC/BPS might have influenced the study outcomes. However, considering the minority representation of Hunner lesions (5–7%), the impact on the study results is probably marginal. As to the evaluation of mast cells, nonspecific staining may occur, which is a significant limitation of these results. Furthermore, we did not perform functional analysis on these models, considering that invasive procedures might cause tissue injury and lead to additional molecular changes. Therefore, evaluating both function and molecular patterns in the same animal might not be reliable. Fortunately, the functional pattern was evidenced and well established^11^. Based on this, using intravitreal injection (IVI) of chemotherapeutic agents in animal models might also induce unnecessary tissue micro-irritation. We did not include a control group for the IVI intervention, which is another limitation of our study.

## Conclusion

The four commonly used IC/BPS rat models (HCL, AA, PS + LPS, and CYP) differed significantly in their histopathological patterns and GO and KEGG pathways, which demonstrated that none of these rat models could fully reflect the complexity of IC/BPS. The present study’s findings suggest that future studies should include multiple models to obtain a comprehensive evaluation of IC/BPS.

### Supplementary Information

Supplementary Tables.

## Data Availability

The datasets generated and/or analyzed in this study are available from the corresponding author on reasonable request.
