# Supplementary material for: Divergent histopathological and molecular patterns in chemically induced interstitial cystitis/bladder pain syndrome rat models
Source: Sci Rep. 2024 Jul 12;14:16134. doi: 10.1038/s41598-024-67162-y (PMC11245554; doi:10.1038/s41598-024-67162-y)
Supplement: Supplementary file 1 — Supplementary Tables. [file 41598_2024_67162_MOESM1_ESM.docx]

Supplementary Table 1. List of significantly upregulated genes in the HCL-induced model compared to the control group

| Gene id | | Gene name | | Log_2_(Fold Change) | | p_Value |
| --- | --- | --- | --- | --- | --- | --- |
| ENSRNOG00000051481 | SCARNA18 | | 28.323 | | 0.02395 | |
| ENSRNOG00000032130 | LOC691427 | | 19.766 | | 0.0017 | |
| ENSRNOG00000050257 | AABR07003273.1 | | 16.812 | | 0.02395 | |
| ENSRNOG00000060174 | AABR07070775.1 | | 16.163 | | 0.00005 | |
| ENSRNOG00000022724 | Cnih3 | | 16.070 | | 0.00145 | |
| ENSRNOG00000047706 | RT1-CE16 | | 7.778 | | 0.00005 | |
| ENSRNOG00000046414 | LOC108348048 | | 5.722 | | 0.0026 | |
| ENSRNOG00000045569 | Nol8 | | 4.615 | | 0.00005 | |
| ENSRNOG00000050407 | NEWGENE_1305281 | | 4.311 | | 0.00005 | |
| ENSRNOG00000009508 | Casp6 | | 4.123 | | 0.00005 | |
| ENSRNOG00000028356 | LOC103694872 | | 4.095 | | 0.00005 | |
| ENSRNOG00000059348 | Ube2b | | 3.820 | | 0.00005 | |
| ENSRNOG00000046379 | LOC100912604 | | 3.665 | | 0.00005 | |
| ENSRNOG00000061814 | Dgcr2 | | 3.643 | | 0.00005 | |
| ENSRNOG00000018712 | Camk2a | | 3.180 | | 0.00005 | |
| ENSRNOG00000058439 | Fam50a | | 3.126 | | 0.00005 | |
| ENSRNOG00000007334 | Ly6h | | 3.110 | | 0.0118 | |
| ENSRNOG00000054334 | Abhd10 | | 2.982 | | 0.0017 | |
| ENSRNOG00000061813 | Ly6c | | 2.976 | | 0.0002 | |
| ENSRNOG00000050450 | LOC100911951 | | 2.888 | | 0.00805 | |
| ENSRNOG00000020650 | Slc17a7 | | 2.769 | | 0.0011 | |
| ENSRNOG00000048365 | LOC100911625 | | 2.738 | | 0.0001 | |
| ENSRNOG00000046250 | LOC100911027 | | 2.647 | | 0.0144 | |
| ENSRNOG00000037793 | Cdk5r2 | | 2.577 | | 0.0041 | |
| ENSRNOG00000006037 | Snap25 | | 2.513 | | 0.00005 | |
| ENSRNOG00000019473 | NEWGENE_1582994 | | 2.489 | | 0.00045 | |
| ENSRNOG00000018039 | Sncb | | 2.421 | | 0.01505 | |
| ENSRNOG00000049802 | AABR07031533.1 | | 2.397 | | 0.0138 | |
| ENSRNOG00000048297 | LOC108348175 | | 2.382 | | 0.00005 | |
| ENSRNOG00000048295 | Dmrtc1a | | 2.376 | | 0.0013 | |
| ENSRNOG00000047396 | Rmnd5b | | 2.359 | | 0.00005 | |
| ENSRNOG00000049575 | Atn1 | | 2.333 | | 0.00005 | |
| ENSRNOG00000030222 | Tcaf2 | | 2.327 | | 0.00005 | |
| ENSRNOG00000026055 | Neurod6 | | 2.320 | | 0.0015 | |
| ENSRNOG00000048187 | LOC691153 | | 2.308 | | 0.00175 | |
| ENSRNOG00000046246 | Spon2 | | 2.295 | | 0.0017 | |
| ENSRNOG00000024149 | Prr18 | | 2.259 | | 0.0174 | |
| ENSRNOG00000049868 | LOC100909725 | | 2.245 | | 0.0097 | |
| ENSRNOG00000056585 | Fscn1 | | 2.234 | | 0.00005 | |
| ENSRNOG00000057527 | RNase_MRP | | 2.222 | | 0.00145 | |
| ENSRNOG00000002456 | Hlf | | 2.195 | | 0.00005 | |
| ENSRNOG00000008225 | AABR07064349.1 | | 2.144 | | 0.0234 | |
| ENSRNOG00000005479 | Slc1a2 | | 2.140 | | 0.0204 | |
| ENSRNOG00000050877 | Cetn3 | | 2.109 | | 0.00005 | |
| ENSRNOG00000013209 | Barhl1 | | 2.078 | | 0.00005 | |
| ENSRNOG00000011445 | Nkain1 | | 2.077 | | 0.0106 | |
| ENSRNOG00000028436 | Rprml | | 2.062 | | 0.00325 | |
| ENSRNOG00000003054 | Cask | | 2.056 | | 0.0349 | |
| ENSRNOG00000013657 | Stmn3 | | 2.056 | | 0.0043 | |
| ENSRNOG00000019587 | Ptprn | | 2.052 | | 0.00005 | |
| ENSRNOG00000047907 | Mapk7 | | 2.047 | | 0.0001 | |
| ENSRNOG00000038539 | Lrrc15 | | 2.028 | | 0.0011 | |
| ENSRNOG00000047112 | LOC100912537 | | 2.016 | | 0.00025 | |
| ENSRNOG00000006970 | Ntn3 | | 2.014 | | 0.00005 | |
| ENSRNOG00000017002 | Adrb1 | | 1.997 | | 0.00005 | |
| ENSRNOG00000020263 | Atp1a3 | | 1.980 | | 0.00005 | |
| ENSRNOG00000050453 | Fbxo27 | | 1.979 | | 0.0146 | |
| ENSRNOG00000047901 | Acp7 | | 1.939 | | 0.00045 | |
| ENSRNOG00000053703 | Rn50_7_1411.1 | | 1.930 | | 0.0059 | |
| ENSRNOG00000049491 | RT1-DMb | | 1.926 | | 0.00005 | |
| ENSRNOG00000014007 | Gfod1 | | 1.921 | | 0.00005 | |
| ENSRNOG00000049598 | LOC100912571 | | 1.896 | | 0.00005 | |
| ENSRNOG00000011078 | Srm | | 1.876 | | 0.0021 | |
| ENSRNOG00000009437 | LOC100912481 | | 1.866 | | 0.00005 | |
| ENSRNOG00000007778 | Alox15b | | 1.851 | | 0.00035 | |
| ENSRNOG00000057522 | Fam134b | | 1.809 | | 0.00005 | |
| ENSRNOG00000056151 | Rasgrf2 | | 1.801 | | 0.00045 | |
| ENSRNOG00000038406 | Tmem132b | | 1.800 | | 0.00075 | |
| ENSRNOG00000001167 | Ubash3a | | 1.783 | | 0.0006 | |
| ENSRNOG00000001747 | Pak2 | | 1.783 | | 0.00005 | |
| ENSRNOG00000016444 | AABR07067506.1 | | 1.781 | | 0.00005 | |
| ENSRNOG00000019207 | Shank1 | | 1.763 | | 0.00215 | |
| ENSRNOG00000000455 | Tap2 | | 1.760 | | 0.00005 | |
| ENSRNOG00000047125 | Foxl1 | | 1.749 | | 0.00005 | |
| ENSRNOG00000036762 | Itpripl2 | | 1.744 | | 0.00005 | |
| ENSRNOG00000053699 | AC096809.1 | | 1.742 | | 0.0004 | |
| ENSRNOG00000022146 | Hist1h2af | | 1.739 | | 0.0026 | |
| ENSRNOG00000028826 | AC128901.1 | | 1.728 | | 0.03595 | |
| ENSRNOG00000013578 | Trem2 | | 1.717 | | 0.00005 | |
| ENSRNOG00000011271 | Mcc | | 1.698 | | 0.00005 | |
| ENSRNOG00000058248 | Rn60_6_0648.1 | | 1.697 | | 0.0106 | |
| ENSRNOG00000021685 | Cdk5r1 | | 1.690 | | 0.00765 | |
| ENSRNOG00000024294 | AABR07019083.1 | | 1.684 | | 0.00005 | |
| ENSRNOG00000020628 | LOC100361913 | | 1.681 | | 0.00005 | |
| ENSRNOG00000012477 | Eef1a2 | | 1.678 | | 0.00005 | |
| ENSRNOG00000060594 | LOC100911672 | | 1.667 | | 0.00005 | |
| ENSRNOG00000007354 | Trpa1 | | 1.659 | | 0.00005 | |
| ENSRNOG00000000500 | Scube3 | | 1.639 | | 0.00005 | |
| ENSRNOG00000021437 | AABR07073181.1 | | 1.623 | | 0.00005 | |
| ENSRNOG00000053373 | AC140765.1 | | 1.620 | | 0.00005 | |
| ENSRNOG00000045543 | Frat2 | | 1.605 | | 0.0028 | |
| ENSRNOG00000001892 | Tbx1 | | 1.602 | | 0.00005 | |
| ENSRNOG00000013661 | Kif26a | | 1.602 | | 0.00005 | |
| ENSRNOG00000046699 | Slpi | | 1.583 | | 0.00005 | |
| ENSRNOG00000060341 | AABR07031399.1 | | 1.576 | | 0.00145 | |
| ENSRNOG00000022248 | Fbxl16 | | 1.571 | | 0.0013 | |
| ENSRNOG00000015428 | Mff | | 1.569 | | 0.00005 | |
| ENSRNOG00000050995 | AABR07002774.2 | | 1.563 | | 0.0134 | |
| ENSRNOG00000047880 | LOC103689975 | | 1.556 | | 0.00005 | |
| ENSRNOG00000006144 | Cntn3 | | 1.538 | | 0.00005 | |
| ENSRNOG00000053206 | LOC688442 | | 1.530 | | 0.0001 | |
| ENSRNOG00000018082 | Slc26a2 | | 1.528 | | 0.00005 | |
| ENSRNOG00000006853 | Elavl2 | | 1.526 | | 0.00055 | |
| ENSRNOG00000060041 | AABR07002677.2 | | 1.525 | | 0.00005 | |
| ENSRNOG00000036598 | LOC685933 | | 1.519 | | 0.0007 | |
| ENSRNOG00000051456 | AABR07013729.1 | | 1.508 | | 0.0013 | |
| ENSRNOG00000060061 | Sowaha | | 1.502 | | 0.00005 | |
| ENSRNOG00000030246 | Btla | | 1.494 | | 0.0069 | |
| ENSRNOG00000001746 | Ncbp2 | | 1.483 | | 0.00005 | |
| ENSRNOG00000047734 | Chst2 | | 1.478 | | 0.00005 | |
| ENSRNOG00000000274 | Phyhipl | | 1.467 | | 0.01325 | |
| ENSRNOG00000004505 | Nfic | | 1.463 | | 0.00005 | |
| ENSRNOG00000027742 | Adamtsl2 | | 1.459 | | 0.00005 | |
| ENSRNOG00000052761 | AC095390.1 | | 1.453 | | 0.04535 | |
| ENSRNOG00000014678 | Fzd5 | | 1.450 | | 0.00005 | |
| ENSRNOG00000004744 | Fam84b | | 1.450 | | 0.00005 | |
| ENSRNOG00000046500 | Irgq | | 1.450 | | 0.00005 | |
| ENSRNOG00000033588 | Siglec15 | | 1.449 | | 0.00005 | |
| ENSRNOG00000053728 | AABR07006724.1 | | 1.446 | | 0.0079 | |
| ENSRNOG00000050395 | Ms4a6e | | 1.421 | | 0.0001 | |
| ENSRNOG00000032522 | RGD1560784 | | 1.419 | | 0.04715 | |
| ENSRNOG00000025735 | Wdr86 | | 1.417 | | 0.01215 | |
| ENSRNOG00000045840 | Hist1h4m | | 1.417 | | 0.0212 | |
| ENSRNOG00000050211 | Rn50_15_0538.1 | | 1.412 | | 0.02385 | |
| ENSRNOG00000014266 | RGD1560730 | | 1.412 | | 0.0001 | |
| ENSRNOG00000059605 | Ddn | | 1.409 | | 0.00005 | |
| ENSRNOG00000048358 | LOC100911107 | | 1.407 | | 0.01745 | |
| ENSRNOG00000029115 | RGD1564883 | | 1.399 | | 0.00005 | |
| ENSRNOG00000011320 | Igfbpl1 | | 1.399 | | 0.00005 | |
| ENSRNOG00000050855 | LOC100911994 | | 1.389 | | 0.00005 | |
| ENSRNOG00000019869 | Lrfn1 | | 1.382 | | 0.00005 | |
| ENSRNOG00000022635 | Dlg2 | | 1.381 | | 0.0005 | |
| ENSRNOG00000017193 | Lingo1 | | 1.380 | | 0.00005 | |
| ENSRNOG00000014723 | Cbfa2t3 | | 1.377 | | 0.00005 | |
| ENSRNOG00000050108 | LOC100911319 | | 1.377 | | 0.00005 | |
| ENSRNOG00000048337 | LOC102551606 | | 1.368 | | 0.01145 | |
| ENSRNOG00000047516 | Map3k7 | | 1.361 | | 0.0272 | |
| ENSRNOG00000029707 | Mt-nd4 | | 1.352 | | 0.00105 | |
| ENSRNOG00000042717 | Ciart | | 1.345 | | 0.00005 | |
| ENSRNOG00000048118 | LOC100910438 | | 1.334 | | 0.00005 | |
| ENSRNOG00000032575 | Ocel1 | | 1.333 | | 0.00005 | |
| ENSRNOG00000004155 | Samd14 | | 1.329 | | 0.00105 | |
| ENSRNOG00000002953 | P2ry4 | | 1.324 | | 0.00005 | |
| ENSRNOG00000048897 | Vmac | | 1.314 | | 0.00005 | |
| ENSRNOG00000013439 | Cpn1 | | 1.313 | | 0.0011 | |
| ENSRNOG00000059720 | Syp | | 1.309 | | 0.00005 | |
| ENSRNOG00000004578 | Cthrc1 | | 1.309 | | 0.00005 | |
| ENSRNOG00000050509 | Ebi3 | | 1.308 | | 0.00105 | |
| ENSRNOG00000006620 | Xcr1 | | 1.306 | | 0.00005 | |
| ENSRNOG00000031778 | Mef2d | | 1.303 | | 0.00005 | |
| ENSRNOG00000056248 | Marf1 | | 1.302 | | 0.00005 | |
| ENSRNOG00000048651 | Nrtn | | 1.300 | | 0.00005 | |
| ENSRNOG00000057943 | AABR07058884.2 | | 1.296 | | 0.03685 | |
| ENSRNOG00000025261 | LOC691254 | | 1.290 | | 0.0045 | |
| ENSRNOG00000017463 | Bloc1s3 | | 1.289 | | 0.00005 | |
| ENSRNOG00000058515 | AABR07054460.4 | | 1.287 | | 0.0007 | |
| ENSRNOG00000001868 | LOC100911248 | | 1.286 | | 0.00005 | |
| ENSRNOG00000018582 | Exosc6 | | 1.286 | | 0.0001 | |
| ENSRNOG00000004757 | Tmem158 | | 1.284 | | 0.00005 | |
| ENSRNOG00000036964 | Ralgapa2 | | 1.282 | | 0.00005 | |
| ENSRNOG00000028266 | Lrrc55 | | 1.282 | | 0.00035 | |
| ENSRNOG00000033741 | Ankrd34a | | 1.281 | | 0.00005 | |
| ENSRNOG00000031599 | Sh2d1b | | 1.276 | | 0.03315 | |
| ENSRNOG00000052080 | Camk2b | | 1.275 | | 0.00005 | |
| ENSRNOG00000009329 | Nr1d1 | | 1.275 | | 0.00005 | |
| ENSRNOG00000018226 | Zcchc14 | | 1.274 | | 0.00005 | |
| ENSRNOG00000018384 | Adam12 | | 1.270 | | 0.00005 | |
| ENSRNOG00000009470 | Flnb | | 1.266 | | 0.00005 | |
| ENSRNOG00000052395 | AABR07071395.1 | | 1.265 | | 0.00065 | |
| ENSRNOG00000032639 | Foxo6 | | 1.261 | | 0.00005 | |
| ENSRNOG00000021891 | Zdhhc8 | | 1.257 | | 0.00005 | |
| ENSRNOG00000023411 | Vsig10l | | 1.254 | | 0.00005 | |
| ENSRNOG00000011423 | Pitx1 | | 1.251 | | 0.00005 | |
| ENSRNOG00000001792 | Slc12a8 | | 1.247 | | 0.00005 | |
| ENSRNOG00000015366 | Neurl3 | | 1.245 | | 0.00015 | |
| ENSRNOG00000042455 | Tlr12 | | 1.244 | | 0.00005 | |
| ENSRNOG00000024065 | Zfp575 | | 1.240 | | 0.0001 | |
| ENSRNOG00000049028 | C5ar2 | | 1.239 | | 0.00005 | |
| ENSRNOG00000053240 | Soga1 | | 1.238 | | 0.00005 | |
| ENSRNOG00000049132 | Map2k3 | | 1.235 | | 0.00005 | |
| ENSRNOG00000006283 | LOC100362400 | | 1.234 | | 0.00005 | |
| ENSRNOG00000048146 | Hoxa9 | | 1.230 | | 0.00005 | |
| ENSRNOG00000036631 | Ptgdr2 | | 1.227 | | 0.00005 | |
| ENSRNOG00000022934 | RGD1562399 | | 1.223 | | 0.00005 | |
| ENSRNOG00000043331 | Ankrd65 | | 1.221 | | 0.0244 | |
| ENSRNOG00000051690 | Clec9a | | 1.216 | | 0.00005 | |
| ENSRNOG00000018867 | Klhdc7a | | 1.216 | | 0.00005 | |
| ENSRNOG00000051160 | Fendrr | | 1.215 | | 0.00005 | |
| ENSRNOG00000019181 | Synpo | | 1.213 | | 0.00005 | |
| ENSRNOG00000010600 | Cysrt1 | | 1.211 | | 0.00005 | |
| ENSRNOG00000049700 | Cda | | 1.210 | | 0.0044 | |
| ENSRNOG00000021174 | Macrod1 | | 1.209 | | 0.00075 | |
| ENSRNOG00000021063 | Grin2d | | 1.208 | | 0.00005 | |
| ENSRNOG00000021021 | Ffar2 | | 1.205 | | 0.028 | |
| ENSRNOG00000001860 | Ccdc116 | | 1.202 | | 0.0006 | |
| ENSRNOG00000032018 | Tmem200b | | 1.198 | | 0.00005 | |
| ENSRNOG00000011856 | LOC108348154 | | 1.195 | | 0.0002 | |
| ENSRNOG00000027489 | Mn1 | | 1.194 | | 0.00005 | |
| ENSRNOG00000033921 | Als2cl | | 1.192 | | 0.00005 | |
| ENSRNOG00000052415 | Gstt2 | | 1.188 | | 0.00005 | |
| ENSRNOG00000002462 | LOC108348055 | | 1.184 | | 0.00005 | |
| ENSRNOG00000045615 | LOC688286 | | 1.178 | | 0.0002 | |
| ENSRNOG00000023972 | Col4a2 | | 1.174 | | 0.00005 | |
| ENSRNOG00000053232 | Ror2 | | 1.174 | | 0.00005 | |
| ENSRNOG00000059500 | Cdkn1c | | 1.173 | | 0.00005 | |
| ENSRNOG00000007657 | Col27a1 | | 1.172 | | 0.00005 | |
| ENSRNOG00000055745 | AABR07043748.2 | | 1.171 | | 0.00005 | |
| ENSRNOG00000028390 | Hhipl1 | | 1.165 | | 0.00005 | |
| ENSRNOG00000001656 | Kcnj15 | | 1.165 | | 0.00775 | |
| ENSRNOG00000039587 | Wdr13 | | 1.162 | | 0.00005 | |
| ENSRNOG00000032026 | AABR07058914.1 | | 1.159 | | 0.00015 | |
| ENSRNOG00000042182 | Mroh2a | | 1.157 | | 0.00005 | |
| ENSRNOG00000007078 | Wisp1 | | 1.156 | | 0.0002 | |
| ENSRNOG00000027606 | Neurl1b | | 1.156 | | 0.0001 | |
| ENSRNOG00000020839 | Lrfn3 | | 1.155 | | 0.00005 | |
| ENSRNOG00000008056 | Ankrd9 | | 1.155 | | 0.00005 | |
| ENSRNOG00000010688 | Nrgn | | 1.153 | | 0.0002 | |
| ENSRNOG00000017531 | Akr1c3 | | 1.153 | | 0.00705 | |
| ENSRNOG00000009173 | Smad6 | | 1.153 | | 0.00005 | |
| ENSRNOG00000000860 | Vwa7 | | 1.152 | | 0.0005 | |
| ENSRNOG00000020399 | Ltb4r | | 1.148 | | 0.001 | |
| ENSRNOG00000048907 | AABR07010563.1 | | 1.147 | | 0.00005 | |
| ENSRNOG00000014375 | Adgrb2 | | 1.146 | | 0.00005 | |
| ENSRNOG00000015664 | Tmem8b | | 1.139 | | 0.00005 | |
| ENSRNOG00000042062 | AC105662.1 | | 1.139 | | 0.0441 | |
| ENSRNOG00000000964 | AABR07036087.1 | | 1.137 | | 0.00005 | |
| ENSRNOG00000010517 | Fam126a | | 1.135 | | 0.00005 | |
| ENSRNOG00000050573 | Tgfbr3l | | 1.134 | | 0.00005 | |
| ENSRNOG00000048004 | Garem2 | | 1.134 | | 0.00035 | |
| ENSRNOG00000007514 | Sox12 | | 1.133 | | 0.00005 | |
| ENSRNOG00000023643 | Mmp17 | | 1.131 | | 0.00005 | |
| ENSRNOG00000009113 | Marcksl1 | | 1.129 | | 0.00005 | |
| ENSRNOG00000048161 | Tlr9 | | 1.129 | | 0.0005 | |
| ENSRNOG00000057978 | AABR07028997.1 | | 1.129 | | 0.00005 | |
| ENSRNOG00000030830 | Mex3d | | 1.127 | | 0.00005 | |
| ENSRNOG00000007387 | Per1 | | 1.126 | | 0.00005 | |
| ENSRNOG00000020784 | Kcnk7 | | 1.124 | | 0.00005 | |
| ENSRNOG00000006204 | Slc30a3 | | 1.124 | | 0.0002 | |
| ENSRNOG00000025350 | Ppp1r13l | | 1.121 | | 0.00005 | |
| ENSRNOG00000013474 | Casz1 | | 1.120 | | 0.00005 | |
| ENSRNOG00000007927 | Mettl7b | | 1.119 | | 0.0449 | |
| ENSRNOG00000016980 | Qprt | | 1.119 | | 0.0123 | |
| ENSRNOG00000061205 | SCARNA17 | | 1.118 | | 0.0129 | |
| ENSRNOG00000008749 | Col5a1 | | 1.118 | | 0.00005 | |
| ENSRNOG00000006450 | Erbb2 | | 1.115 | | 0.00005 | |
| ENSRNOG00000012175 | Nfatc2 | | 1.115 | | 0.00005 | |
| ENSRNOG00000000288 | Scarf2 | | 1.111 | | 0.00005 | |
| ENSRNOG00000001094 | Zfp316 | | 1.109 | | 0.00005 | |
| ENSRNOG00000051948 | Hcfc1 | | 1.109 | | 0.00005 | |
| ENSRNOG00000051915 | Spred3 | | 1.106 | | 0.00005 | |
| ENSRNOG00000057235 | AABR07067355.1 | | 1.105 | | 0.00005 | |
| ENSRNOG00000002419 | Plp1 | | 1.099 | | 0.00005 | |
| ENSRNOG00000021039 | Fam83e | | 1.096 | | 0.00005 | |
| ENSRNOG00000042414 | Nat14 | | 1.096 | | 0.00015 | |
| ENSRNOG00000003463 | Srebf1 | | 1.096 | | 0.00005 | |
| ENSRNOG00000060528 | Cacna1g | | 1.095 | | 0.00005 | |
| ENSRNOG00000023968 | Fancf | | 1.094 | | 0.00005 | |
| ENSRNOG00000039197 | LOC108348074 | | 1.092 | | 0.00005 | |
| ENSRNOG00000022143 | Dusp23 | | 1.090 | | 0.0001 | |
| ENSRNOG00000012280 | Ptx3 | | 1.089 | | 0.0336 | |
| ENSRNOG00000032368 | Tlr11 | | 1.089 | | 0.0003 | |
| ENSRNOG00000043102 | Bahcc1 | | 1.088 | | 0.00005 | |
| ENSRNOG00000016617 | Wwtr1 | | 1.087 | | 0.00005 | |
| ENSRNOG00000012843 | Aspg | | 1.085 | | 0.00005 | |
| ENSRNOG00000001309 | Camkk2 | | 1.085 | | 0.00005 | |
| ENSRNOG00000019570 | Gng3 | | 1.085 | | 0.01995 | |
| ENSRNOG00000010649 | Ctnnd2 | | 1.084 | | 0.00005 | |
| ENSRNOG00000005008 | Angpt4 | | 1.083 | | 0.0008 | |
| ENSRNOG00000060916 | AC126134.1 | | 1.082 | | 0.0006 | |
| ENSRNOG00000006916 | Sardh | | 1.081 | | 0.00005 | |
| ENSRNOG00000016258 | Zfp516 | | 1.078 | | 0.00005 | |
| ENSRNOG00000013829 | Chrna3 | | 1.077 | | 0.00155 | |
| ENSRNOG00000018326 | Pgls | | 1.076 | | 0.00005 | |
| ENSRNOG00000017489 | Gse1 | | 1.076 | | 0.00005 | |
| ENSRNOG00000048273 | Apod | | 1.075 | | 0.00005 | |
| ENSRNOG00000027295 | LOC108348064 | | 1.073 | | 0.00015 | |
| ENSRNOG00000058202 | Ppp2r2c | | 1.072 | | 0.00035 | |
| ENSRNOG00000034075 | Ube2ql1 | | 1.071 | | 0.00045 | |
| ENSRNOG00000022421 | Crtc1 | | 1.071 | | 0.00005 | |
| ENSRNOG00000060010 | Ss18l1 | | 1.070 | | 0.0002 | |
| ENSRNOG00000019418 | Lrrc4b | | 1.070 | | 0.00005 | |
| ENSRNOG00000028350 | Arse | | 1.069 | | 0.00005 | |
| ENSRNOG00000032908 | Acaa1b | | 1.068 | | 0.00005 | |
| ENSRNOG00000046968 | Nol8 | | 1.067 | | 0.00005 | |
| ENSRNOG00000032232 | Snrpg | | 1.065 | | 0.00025 | |
| ENSRNOG00000023931 | Ggn | | 1.063 | | 0.00005 | |
| ENSRNOG00000029264 | Kcnn1 | | 1.063 | | 0.00025 | |
| ENSRNOG00000033791 | Apc2 | | 1.062 | | 0.0043 | |
| ENSRNOG00000050024 | Ms4a4a | | 1.061 | | 0.00145 | |
| ENSRNOG00000048168 | Gtpbp6 | | 1.060 | | 0.0022 | |
| ENSRNOG00000029594 | AABR07064000.1 | | 1.059 | | 0.00005 | |
| ENSRNOG00000059618 | Scarf1 | | 1.059 | | 0.00005 | |
| ENSRNOG00000034200 | Atp8a1 | | 1.059 | | 0.00055 | |
| ENSRNOG00000022110 | Gcsam | | 1.058 | | 0.00015 | |
| ENSRNOG00000046159 | Gns | | 1.058 | | 0.00005 | |
| ENSRNOG00000030689 | Ms4a6bl | | 1.057 | | 0.035 | |
| ENSRNOG00000055340 | Ski | | 1.057 | | 0.00005 | |
| ENSRNOG00000013416 | Arid3c | | 1.057 | | 0.00005 | |
| ENSRNOG00000006784 | Sema4f | | 1.057 | | 0.00005 | |
| ENSRNOG00000014987 | Mdfi | | 1.055 | | 0.00005 | |
| ENSRNOG00000050669 | LOC100911515 | | 1.055 | | 0.00005 | |
| ENSRNOG00000024657 | Mfsd4 | | 1.055 | | 0.00055 | |
| ENSRNOG00000003645 | LOC103689931 | | 1.053 | | 0.0003 | |
| ENSRNOG00000010758 | Lpar2 | | 1.052 | | 0.0078 | |
| ENSRNOG00000004819 | Porcn | | 1.051 | | 0.00005 | |
| ENSRNOG00000004753 | Napb | | 1.050 | | 0.03455 | |
| ENSRNOG00000050714 | Islr2 | | 1.049 | | 0.00035 | |
| ENSRNOG00000028624 | Kif26b | | 1.048 | | 0.00005 | |
| ENSRNOG00000039207 | Ccdc71l | | 1.047 | | 0.0018 | |
| ENSRNOG00000029012 | Shisa6 | | 1.045 | | 0.00005 | |
| ENSRNOG00000039668 | Col8a1 | | 1.044 | | 0.00005 | |
| ENSRNOG00000004346 | Notch3 | | 1.041 | | 0.00005 | |
| ENSRNOG00000010650 | Plekhh1 | | 1.041 | | 0.00005 | |
| ENSRNOG00000010665 | Ccr7 | | 1.041 | | 0.02045 | |
| ENSRNOG00000049330 | NEWGENE_1308196 | | 1.036 | | 0.0091 | |
| ENSRNOG00000043300 | Enho | | 1.036 | | 0.00005 | |
| ENSRNOG00000010718 | Gpr153 | | 1.035 | | 0.00005 | |
| ENSRNOG00000033496 | Igdcc4 | | 1.034 | | 0.00015 | |
| ENSRNOG00000001729 | Xxylt1 | | 1.033 | | 0.00005 | |
| ENSRNOG00000015304 | Tmem160 | | 1.031 | | 0.00005 | |
| ENSRNOG00000037327 | F8a1 | | 1.031 | | 0.00005 | |
| ENSRNOG00000021067 | Fxyd7 | | 1.031 | | 0.0055 | |
| ENSRNOG00000008296 | Tpgs1 | | 1.030 | | 0.00005 | |
| ENSRNOG00000029450 | Bai1 | | 1.030 | | 0.0067 | |
| ENSRNOG00000009446 | Rxra | | 1.024 | | 0.00005 | |
| ENSRNOG00000049034 | Ptrh1 | | 1.022 | | 0.03525 | |
| ENSRNOG00000015858 | Hyal1 | | 1.021 | | 0.00005 | |
| ENSRNOG00000011026 | Irf2bpl | | 1.021 | | 0.00005 | |
| ENSRNOG00000049970 | RGD1566085 | | 1.019 | | 0.0001 | |
| ENSRNOG00000010107 | Palld | | 1.019 | | 0.00005 | |
| ENSRNOG00000012442 | Cemip | | 1.018 | | 0.00005 | |
| ENSRNOG00000047113 | LOC100911548 | | 1.016 | | 0.0261 | |
| ENSRNOG00000012420 | Bcl9l | | 1.015 | | 0.00005 | |
| ENSRNOG00000003348 | Rasd1 | | 1.015 | | 0.00005 | |
| ENSRNOG00000013460 | Zfp319 | | 1.015 | | 0.00005 | |
| ENSRNOG00000016419 | Pdlim5 | | 1.012 | | 0.00005 | |
| ENSRNOG00000007426 | Tmem64 | | 1.011 | | 0.00005 | |
| ENSRNOG00000017075 | Slc35e2b | | 1.011 | | 0.0003 | |
| ENSRNOG00000018824 | Slc7a5 | | 1.009 | | 0.00005 | |
| ENSRNOG00000030721 | Fbrsl1 | | 1.007 | | 0.00005 | |
| ENSRNOG00000029141 | Trabd2b | | 1.006 | | 0.0003 | |
| ENSRNOG00000004733 | Wdr25 | | 1.005 | | 0.00005 | |
| ENSRNOG00000003195 | Caskin1 | | 1.004 | | 0.00005 | |
| ENSRNOG00000031474 | Rps19l1 | | 1.003 | | 0.0053 | |
| ENSRNOG00000012818 | Ksr1 | | 1.003 | | 0.00005 | |
| ENSRNOG00000003073 | Gpr161 | | 1.002 | | 0.00145 | |
| ENSRNOG00000054314 | Kcng1 | | 1.001 | | 0.00005 | |
| ENSRNOG00000006049 | Rfx1 | | 1.001 | | 0.00005 | |
| ENSRNOG00000004476 | Wif1 | | 1.000 | | 0.0001 | |

Supplementary Table 2. List of significantly downregulated genes in the HCL-induced model compared to the control group

| Gene id | | Gene name | | Log_2_(Fold Change) | | p_Value |
| --- | --- | --- | --- | --- | --- | --- |
| ENSRNOG00000035103 | 5S_rRNA | | -23.188 | | 0.00135 | |
| ENSRNOG00000054722 | U1 | | -19.970 | | 0.02285 | |
| ENSRNOG00000048073 | LOC103690821 | | -19.868 | | 0.00135 | |
| ENSRNOG00000055311 | U1 | | -19.700 | | 0.0077 | |
| ENSRNOG00000051920 | U1 | | -19.409 | | 0.0036 | |
| ENSRNOG00000043400 | LOC100910678 | | -19.144 | | 0.0012 | |
| ENSRNOG00000059703 | AC123425.1 | | -18.500 | | 0.00015 | |
| ENSRNOG00000052706 | AABR07072841.1 | | -18.132 | | 0.0058 | |
| ENSRNOG00000028311 | Cnpy1 | | -17.673 | | 0.0481 | |
| ENSRNOG00000043480 | Timm8a1 | | -17.365 | | 0.02285 | |
| ENSRNOG00000061507 | AABR07032328.1 | | -16.982 | | 0.02285 | |
| ENSRNOG00000057895 | AABR07033745.1 | | -16.764 | | 0.02285 | |
| ENSRNOG00000038735 | AABR07064702.1 | | -16.699 | | 0.0001 | |
| ENSRNOG00000048585 | AABR07018244.1 | | -16.427 | | 0.00135 | |
| ENSRNOG00000057266 | Rnase1 | | -16.327 | | 0.0001 | |
| ENSRNOG00000059962 | AABR07025787.1 | | -16.152 | | 0.00135 | |
| ENSRNOG00000054216 | AABR07056858.1 | | -16.008 | | 0.0001 | |
| ENSRNOG00000051321 | Rn50_X_0711.1 | | -15.987 | | 0.0001 | |
| ENSRNOG00000045790 | LOC100910130 | | -4.582 | | 0.00005 | |
| ENSRNOG00000004247 | Nhp2 | | -4.570 | | 0.04575 | |
| ENSRNOG00000020781 | LOC103690005 | | -4.204 | | 0.00005 | |
| ENSRNOG00000053109 | Mrpl53 | | -4.055 | | 0.00265 | |
| ENSRNOG00000021555 | Mis18a | | -3.994 | | 0.04575 | |
| ENSRNOG00000051591 | LOC100911422 | | -3.792 | | 0.00005 | |
| ENSRNOG00000046120 | RGD1561252 | | -3.500 | | 0.03225 | |
| ENSRNOG00000006589 | LOC103694877 | | -3.324 | | 0.04885 | |
| ENSRNOG00000010640 | Agtr1b | | -3.245 | | 0.04815 | |
| ENSRNOG00000010603 | Gzma | | -3.199 | | 0.00395 | |
| ENSRNOG00000057515 | Arf3 | | -3.194 | | 0.00545 | |
| ENSRNOG00000013141 | Eno2 | | -3.128 | | 0.00005 | |
| ENSRNOG00000003136 | Fcrla | | -2.975 | | 0.00005 | |
| ENSRNOG00000027079 | Impad1 | | -2.907 | | 0.0429 | |
| ENSRNOG00000055809 | LOC100911769 | | -2.882 | | 0.00005 | |
| ENSRNOG00000061096 | Rn7sl1 | | -2.860 | | 0.00005 | |
| ENSRNOG00000000201 | Gsta5 | | -2.702 | | 0.00005 | |
| ENSRNOG00000052664 | Tnks2 | | -2.671 | | 0.00005 | |
| ENSRNOG00000046548 | Rhox5 | | -2.657 | | 0.00005 | |
| ENSRNOG00000059883 | LOC100912578 | | -2.632 | | 0.00005 | |
| ENSRNOG00000048882 | LOC100363914 | | -2.565 | | 0.0178 | |
| ENSRNOG00000058424 | AABR07057150.1 | | -2.439 | | 0.00405 | |
| ENSRNOG00000049743 | NEWGENE_620381 | | -2.422 | | 0.0031 | |
| ENSRNOG00000053147 | AC141521.1 | | -2.408 | | 0.0001 | |
| ENSRNOG00000004994 | Agr3 | | -2.259 | | 0.00005 | |
| ENSRNOG00000049814 | LOC100910882 | | -2.248 | | 0.00035 | |
| ENSRNOG00000023622 | LOC100910021 | | -2.233 | | 0.00565 | |
| ENSRNOG00000017619 | Aldh1a1 | | -2.215 | | 0.00005 | |
| ENSRNOG00000000582 | Ddo | | -2.205 | | 0.00215 | |
| ENSRNOG00000008282 | Elf5 | | -2.168 | | 0.0005 | |
| ENSRNOG00000005023 | Agr2 | | -2.128 | | 0.00005 | |
| ENSRNOG00000052651 | LOC102556085 | | -2.111 | | 0.02095 | |
| ENSRNOG00000017412 | AABR07001512.1 | | -2.086 | | 0.00005 | |
| ENSRNOG00000051341 | Rn50_X_0635.2 | | -2.057 | | 0.02575 | |
| ENSRNOG00000054855 | AABR07014855.1 | | -2.036 | | 0.0334 | |
| ENSRNOG00000025957 | Ooep | | -1.987 | | 0.0152 | |
| ENSRNOG00000005247 | Surf1 | | -1.972 | | 0.04215 | |
| ENSRNOG00000034087 | Krt42 | | -1.965 | | 0.00085 | |
| ENSRNOG00000033748 | AABR07060610.1 | | -1.964 | | 0.03575 | |
| ENSRNOG00000000390 | LOC100910755 | | -1.904 | | 0.00005 | |
| ENSRNOG00000016301 | Dmrt2 | | -1.848 | | 0.00005 | |
| ENSRNOG00000033970 | Moap1 | | -1.847 | | 0.01735 | |
| ENSRNOG00000013953 | Ntrk1 | | -1.823 | | 0.0411 | |
| ENSRNOG00000042851 | AC109942.1 | | -1.791 | | 0.0482 | |
| ENSRNOG00000046950 | Ewsr1 | | -1.787 | | 0.00005 | |
| ENSRNOG00000000478 | AA926063 | | -1.754 | | 0.0048 | |
| ENSRNOG00000046832 | Rn50_X_0745.4 | | -1.737 | | 0.00005 | |
| ENSRNOG00000054340 | AABR07011013.1 | | -1.719 | | 0.0053 | |
| ENSRNOG00000036802 | Snhg11 | | -1.693 | | 0.00005 | |
| ENSRNOG00000004706 | Vit | | -1.658 | | 0.00005 | |
| ENSRNOG00000015308 | Pbk | | -1.649 | | 0.0074 | |
| ENSRNOG00000037409 | Scimp | | -1.624 | | 0.02845 | |
| ENSRNOG00000018505 | Cidea | | -1.621 | | 0.0296 | |
| ENSRNOG00000009785 | Cdkn3 | | -1.610 | | 0.0059 | |
| ENSRNOG00000022957 | Ctxn3 | | -1.607 | | 0.00125 | |
| ENSRNOG00000023182 | RGD1565317 | | -1.601 | | 0.00505 | |
| ENSRNOG00000061904 | LOC103694864 | | -1.594 | | 0.00005 | |
| ENSRNOG00000053812 | Selenbp1 | | -1.570 | | 0.00005 | |
| ENSRNOG00000049590 | RT1-M2 | | -1.560 | | 0.01365 | |
| ENSRNOG00000003745 | Atf3 | | -1.545 | | 0.00005 | |
| ENSRNOG00000053047 | Top2a | | -1.533 | | 0.00005 | |
| ENSRNOG00000015519 | Ces1d | | -1.499 | | 0.00005 | |
| ENSRNOG00000055451 | Gcnt4 | | -1.483 | | 0.00285 | |
| ENSRNOG00000015751 | Cyp4f18 | | -1.472 | | 0.00005 | |
| ENSRNOG00000029408 | Mageb16 | | -1.471 | | 0.00005 | |
| ENSRNOG00000012714 | RGD1564149 | | -1.467 | | 0.00035 | |
| ENSRNOG00000047854 | LOC108348072 | | -1.453 | | 0.00005 | |
| ENSRNOG00000060602 | AABR07014275.1 | | -1.440 | | 0.00005 | |
| ENSRNOG00000051672 | AABR07008030.1 | | -1.439 | | 0.0213 | |
| ENSRNOG00000026112 | Tmem202 | | -1.429 | | 0.0206 | |
| ENSRNOG00000049995 | Slc40a1 | | -1.405 | | 0.00735 | |
| ENSRNOG00000018131 | Slc16a4 | | -1.403 | | 0.00265 | |
| ENSRNOG00000002031 | Naa11 | | -1.399 | | 0.00005 | |
| ENSRNOG00000011400 | Ralyl | | -1.393 | | 0.00055 | |
| ENSRNOG00000004635 | Kera | | -1.373 | | 0.00205 | |
| ENSRNOG00000026979 | RGD1561444 | | -1.365 | | 0.0049 | |
| ENSRNOG00000048898 | Wasf3 | | -1.361 | | 0.01375 | |
| ENSRNOG00000060837 | AC132752.2 | | -1.343 | | 0.0444 | |
| ENSRNOG00000008873 | Ino80b | | -1.340 | | 0.00005 | |
| ENSRNOG00000022946 | Slc22a3 | | -1.336 | | 0.00035 | |
| ENSRNOG00000031855 | Actr3b | | -1.327 | | 0.00525 | |
| ENSRNOG00000002158 | Ibsp | | -1.323 | | 0.0004 | |
| ENSRNOG00000011398 | LOC103692719 | | -1.309 | | 0.04775 | |
| ENSRNOG00000045747 | Capns1 | | -1.308 | | 0.00005 | |
| ENSRNOG00000009371 | Abl1 | | -1.300 | | 0.00005 | |
| ENSRNOG00000061630 | AABR07009834.1 | | -1.296 | | 0.01575 | |
| ENSRNOG00000014518 | Adam28 | | -1.282 | | 0.00005 | |
| ENSRNOG00000001967 | Sult1b1 | | -1.282 | | 0.00005 | |
| ENSRNOG00000053400 | Cfi | | -1.272 | | 0.0028 | |
| ENSRNOG00000001893 | LOC100362453 | | -1.267 | | 0.0001 | |
| ENSRNOG00000046663 | LOC100911825 | | -1.265 | | 0.00005 | |
| ENSRNOG00000021355 | Car6 | | -1.262 | | 0.00005 | |
| ENSRNOG00000027139 | Krt20 | | -1.249 | | 0.00005 | |
| ENSRNOG00000025640 | Olr1637 | | -1.247 | | 0.0413 | |
| ENSRNOG00000056722 | AABR07016578.3 | | -1.247 | | 0.0243 | |
| ENSRNOG00000021015 | Sbsn | | -1.247 | | 0.00005 | |
| ENSRNOG00000013312 | Kcnt2 | | -1.246 | | 0.0003 | |
| ENSRNOG00000047911 | Miip | | -1.238 | | 0.0019 | |
| ENSRNOG00000002555 | Serpinb7 | | -1.235 | | 0.00005 | |
| ENSRNOG00000059776 | Tnks2 | | -1.233 | | 0.00005 | |
| ENSRNOG00000031769 | Chchd7 | | -1.210 | | 0.043 | |
| ENSRNOG00000018690 | Rgs17 | | -1.205 | | 0.01115 | |
| ENSRNOG00000002754 | Areg | | -1.198 | | 0.00005 | |
| ENSRNOG00000009530 | Uchl3 | | -1.198 | | 0.00005 | |
| ENSRNOG00000011623 | Rab3c | | -1.191 | | 0.0034 | |
| ENSRNOG00000050006 | Agtr2 | | -1.175 | | 0.01965 | |
| ENSRNOG00000029841 | Cdh19 | | -1.171 | | 0.0004 | |
| ENSRNOG00000038106 | Map1lc3b2 | | -1.171 | | 0.03115 | |
| ENSRNOG00000048088 | Mest | | -1.169 | | 0.00005 | |
| ENSRNOG00000060508 | AABR07028523.1 | | -1.166 | | 0.03235 | |
| ENSRNOG00000002711 | Nuf2 | | -1.162 | | 0.0005 | |
| ENSRNOG00000056863 | RGD1562725 | | -1.152 | | 0.017 | |
| ENSRNOG00000028137 | Mki67 | | -1.150 | | 0.00005 | |
| ENSRNOG00000029911 | Cilp | | -1.144 | | 0.00005 | |
| ENSRNOG00000001821 | Adipoq | | -1.139 | | 0.00005 | |
| ENSRNOG00000019776 | Sh3gl3 | | -1.137 | | 0.00015 | |
| ENSRNOG00000005670 | Art4 | | -1.136 | | 0.00005 | |
| ENSRNOG00000021619 | Cela3b | | -1.136 | | 0.0356 | |
| ENSRNOG00000058920 | Apopt1 | | -1.129 | | 0.00015 | |
| ENSRNOG00000026217 | Armc2 | | -1.117 | | 0.00005 | |
| ENSRNOG00000030069 | Faim | | -1.110 | | 0.00025 | |
| ENSRNOG00000003480 | Aim2 | | -1.104 | | 0.0124 | |
| ENSRNOG00000045779 | Rn50_X_0729.6 | | -1.101 | | 0.00005 | |
| ENSRNOG00000047647 | Il2ra | | -1.099 | | 0.0002 | |
| ENSRNOG00000048608 | Rsl1d1l1 | | -1.093 | | 0.00155 | |
| ENSRNOG00000045797 | Lep | | -1.090 | | 0.0001 | |
| ENSRNOG00000002428 | Lpar4 | | -1.087 | | 0.00005 | |
| ENSRNOG00000001578 | Hoxd4 | | -1.082 | | 0.00005 | |
| ENSRNOG00000021424 | Cd300lf | | -1.080 | | 0.00045 | |
| ENSRNOG00000016100 | Tbca | | -1.076 | | 0.0441 | |
| ENSRNOG00000062232 | Mcc | | -1.076 | | 0.04175 | |
| ENSRNOG00000014603 | Sgcg | | -1.071 | | 0.00205 | |
| ENSRNOG00000056493 | Mybpc1 | | -1.071 | | 0.00005 | |
| ENSRNOG00000015036 | Ctgf | | -1.067 | | 0.00005 | |
| ENSRNOG00000024120 | Rxfp1 | | -1.065 | | 0.00005 | |
| ENSRNOG00000062158 | Rn60_1_2212.4 | | -1.065 | | 0.00005 | |
| ENSRNOG00000013222 | Cd207 | | -1.062 | | 0.0197 | |
| ENSRNOG00000015088 | Prune2 | | -1.060 | | 0.00145 | |
| ENSRNOG00000003209 | Pcp4l1 | | -1.056 | | 0.0303 | |
| ENSRNOG00000016571 | Ngf | | -1.055 | | 0.00905 | |
| ENSRNOG00000005176 | Map7d2 | | -1.048 | | 0.00005 | |
| ENSRNOG00000062133 | Rn60_9_0652.3 | | -1.048 | | 0.04185 | |
| ENSRNOG00000031367 | LOC103689983 | | -1.046 | | 0.00005 | |
| ENSRNOG00000033855 | AABR07062466.1 | | -1.044 | | 0.0484 | |
| ENSRNOG00000012752 | Cklf | | -1.033 | | 0.001 | |
| ENSRNOG00000030106 | RGD1564698 | | -1.031 | | 0.00005 | |
| ENSRNOG00000046848 | PCOLCE2 | | -1.021 | | 0.00005 | |
| ENSRNOG00000010685 | Tbx18 | | -1.020 | | 0.00005 | |
| ENSRNOG00000059010 | Rnf212 | | -1.020 | | 0.0032 | |
| ENSRNOG00000005718 | AABR07065406.1 | | -1.019 | | 0.0015 | |
| ENSRNOG00000006930 | Casq1 | | -1.015 | | 0.00415 | |
| ENSRNOG00000043203 | Krt75 | | -1.015 | | 0.00195 | |
| ENSRNOG00000059894 | Hmmr | | -1.013 | | 0.00055 | |
| ENSRNOG00000005792 | Ankef1 | | -1.011 | | 0.0042 | |
| ENSRNOG00000016391 | LOC100912163 | | -1.011 | | 0.0114 | |
| ENSRNOG00000034191 | Fmo1 | | -1.010 | | 0.00005 | |
| ENSRNOG00000053717 | Metazoa_SRP | | -1.006 | | 0.00045 | |
| ENSRNOG00000004662 | RGD1560687 | | -1.005 | | 0.00225 | |

Supplementary Table 3. List of significantly upregulated genes in the AA-induced model compared to the control group

| Gene id | | Gene name | | Log_2_(Fold Change) | | p_Value |
| --- | --- | --- | --- | --- | --- | --- |
| ENSRNOG00000056555 | AABR07054983.1 | | 19.646 | | 0.00135 | |
| ENSRNOG00000059770 | RT1-CE16 | | 18.868 | | 0.04285 | |
| ENSRNOG00000056094 | AC125873.1 | | 18.756 | | 0.0044 | |
| ENSRNOG00000014118 | Klkb1 | | 17.423 | | 0.00005 | |
| ENSRNOG00000051360 | RGD1563270 | | 16.903 | | 0.00005 | |
| ENSRNOG00000047971 | LOC100910528 | | 16.553 | | 0.01435 | |
| ENSRNOG00000046414 | LOC108348048 | | 5.323 | | 0.00265 | |
| ENSRNOG00000059348 | Ube2b | | 4.313 | | 0.00005 | |
| ENSRNOG00000045655 | Tbcb | | 3.839 | | 0.00025 | |
| ENSRNOG00000060898 | LOC100910418 | | 3.619 | | 0.00005 | |
| ENSRNOG00000053703 | Rn50_7_1411.1 | | 3.469 | | 0.00015 | |
| ENSRNOG00000061814 | Dgcr2 | | 3.447 | | 0.00005 | |
| ENSRNOG00000019206 | Nupr1 | | 3.398 | | 0.00005 | |
| ENSRNOG00000055992 | LOC103690095 | | 3.292 | | 0.00005 | |
| ENSRNOG00000050877 | Cetn3 | | 3.124 | | 0.00005 | |
| ENSRNOG00000011729 | Nxf2 | | 3.117 | | 0.00005 | |
| ENSRNOG00000050193 | LOC100912262 | | 2.835 | | 0.0492 | |
| ENSRNOG00000061813 | Ly6c | | 2.768 | | 0.00005 | |
| ENSRNOG00000047112 | LOC100912537 | | 2.580 | | 0.00005 | |
| ENSRNOG00000048365 | LOC100911625 | | 2.479 | | 0.0001 | |
| ENSRNOG00000047396 | Rmnd5b | | 2.430 | | 0.00005 | |
| ENSRNOG00000060990 | AABR07000382.1 | | 2.372 | | 0.00015 | |
| ENSRNOG00000001747 | Pak2 | | 2.349 | | 0.00005 | |
| ENSRNOG00000030222 | Tcaf2 | | 2.326 | | 0.00005 | |
| ENSRNOG00000028356 | LOC103694872 | | 2.311 | | 0.00005 | |
| ENSRNOG00000059050 | - | | 2.274 | | 0.00005 | |
| ENSRNOG00000011955 | AABR07026997.1 | | 2.056 | | 0.0243 | |
| ENSRNOG00000051621 | Tex40 | | 1.993 | | 0.00005 | |
| ENSRNOG00000058589 | AABR07046778.1 | | 1.990 | | 0.00005 | |
| ENSRNOG00000017897 | Adam8 | | 1.983 | | 0.00005 | |
| ENSRNOG00000046973 | Tmco3 | | 1.938 | | 0.00035 | |
| ENSRNOG00000047581 | LOC100912293 | | 1.935 | | 0.01915 | |
| ENSRNOG00000047368 | Acer1 | | 1.931 | | 0.00005 | |
| ENSRNOG00000058364 | AABR07051515.1 | | 1.915 | | 0.03815 | |
| ENSRNOG00000021902 | Rmi2 | | 1.913 | | 0.04735 | |
| ENSRNOG00000047907 | Mapk7 | | 1.872 | | 0.00535 | |
| ENSRNOG00000027880 | Nbeal2 | | 1.848 | | 0.00005 | |
| ENSRNOG00000058904 | Tex13b | | 1.803 | | 0.00005 | |
| ENSRNOG00000011445 | Nkain1 | | 1.767 | | 0.02055 | |
| ENSRNOG00000042111 | Sult1c2a | | 1.767 | | 0.01065 | |
| ENSRNOG00000060594 | LOC100911672 | | 1.759 | | 0.00005 | |
| ENSRNOG00000030776 | Sytl2 | | 1.755 | | 0.00005 | |
| ENSRNOG00000029843 | LOC100359563 | | 1.751 | | 0.0115 | |
| ENSRNOG00000058862 | AABR07051308.1 | | 1.750 | | 0.00005 | |
| ENSRNOG00000017531 | Akr1c3 | | 1.700 | | 0.00005 | |
| ENSRNOG00000017459 | C1ql3 | | 1.696 | | 0.0032 | |
| ENSRNOG00000049298 | LOC100909595 | | 1.667 | | 0.00595 | |
| ENSRNOG00000049598 | LOC100912571 | | 1.663 | | 0.0046 | |
| ENSRNOG00000007354 | Trpa1 | | 1.641 | | 0.00005 | |
| ENSRNOG00000021140 | Kcnk4 | | 1.636 | | 0.00005 | |
| ENSRNOG00000016444 | AABR07067506.1 | | 1.630 | | 0.00005 | |
| ENSRNOG00000050395 | Ms4a6e | | 1.624 | | 0.00005 | |
| ENSRNOG00000015428 | Mff | | 1.620 | | 0.00005 | |
| ENSRNOG00000050407 | NEWGENE_1305281 | | 1.599 | | 0.00005 | |
| ENSRNOG00000049593 | Wbp11 | | 1.596 | | 0.00005 | |
| ENSRNOG00000050855 | LOC100911994 | | 1.532 | | 0.00005 | |
| ENSRNOG00000042182 | Mroh2a | | 1.514 | | 0.00005 | |
| ENSRNOG00000046968 | Nol8 | | 1.468 | | 0.00005 | |
| ENSRNOG00000020025 | Slc29a2 | | 1.450 | | 0.02945 | |
| ENSRNOG00000029594 | AABR07064000.1 | | 1.447 | | 0.00005 | |
| ENSRNOG00000047516 | Map3k7 | | 1.419 | | 0.01705 | |
| ENSRNOG00000052761 | AC095390.1 | | 1.406 | | 0.0479 | |
| ENSRNOG00000001367 | Gpc2 | | 1.404 | | 0.00005 | |
| ENSRNOG00000051612 | AABR07044570.1 | | 1.396 | | 0.00745 | |
| ENSRNOG00000025946 | Igf2bp2 | | 1.394 | | 0.00005 | |
| ENSRNOG00000008849 | Guca2a | | 1.392 | | 0.00005 | |
| ENSRNOG00000013209 | Barhl1 | | 1.391 | | 0.0006 | |
| ENSRNOG00000008478 | Mmp13 | | 1.390 | | 0.00005 | |
| ENSRNOG00000056435 | LOC102546764 | | 1.383 | | 0.0119 | |
| ENSRNOG00000048357 | LOC103690164 | | 1.359 | | 0.0112 | |
| ENSRNOG00000059500 | Cdkn1c | | 1.358 | | 0.00005 | |
| ENSRNOG00000028436 | Rprml | | 1.328 | | 0.02895 | |
| ENSRNOG00000061432 | AABR07030914.1 | | 1.312 | | 0.02515 | |
| ENSRNOG00000017002 | Adrb1 | | 1.302 | | 0.00005 | |
| ENSRNOG00000012049 | Sox7 | | 1.300 | | 0.00005 | |
| ENSRNOG00000020380 | Lgals7 | | 1.297 | | 0.00595 | |
| ENSRNOG00000012891 | Mdh1b | | 1.275 | | 0.00865 | |
| ENSRNOG00000058439 | Fam50a | | 1.246 | | 0.00295 | |
| ENSRNOG00000021039 | Fam83e | | 1.238 | | 0.00005 | |
| ENSRNOG00000060514 | Wdr54 | | 1.234 | | 0.0058 | |
| ENSRNOG00000022155 | RGD1560289 | | 1.229 | | 0.00005 | |
| ENSRNOG00000001031 | Ocm2 | | 1.225 | | 0.00005 | |
| ENSRNOG00000050509 | Ebi3 | | 1.225 | | 0.0026 | |
| ENSRNOG00000002953 | P2ry4 | | 1.205 | | 0.00005 | |
| ENSRNOG00000021260 | Prnd | | 1.204 | | 0.0431 | |
| ENSRNOG00000058436 | LOC108348215 | | 1.192 | | 0.00005 | |
| ENSRNOG00000037172 | RGD1564138 | | 1.189 | | 0.04385 | |
| ENSRNOG00000047880 | LOC103689975 | | 1.179 | | 0.0001 | |
| ENSRNOG00000020132 | Ttll10 | | 1.172 | | 0.00005 | |
| ENSRNOG00000029792 | NEWGENE_1308171 | | 1.144 | | 0.00005 | |
| ENSRNOG00000020295 | Plekhn1 | | 1.143 | | 0.00005 | |
| ENSRNOG00000012067 | Fam111a | | 1.132 | | 0.00005 | |
| ENSRNOG00000006943 | Zcchc10 | | 1.120 | | 0.0026 | |
| ENSRNOG00000061358 | AC129365.1 | | 1.119 | | 0.0015 | |
| ENSRNOG00000047261 | Mapre2 | | 1.103 | | 0.00055 | |
| ENSRNOG00000016108 | Phlpp2 | | 1.102 | | 0.00005 | |
| ENSRNOG00000016622 | Ankra2 | | 1.099 | | 0.00095 | |
| ENSRNOG00000050573 | Tgfbr3l | | 1.089 | | 0.00005 | |
| ENSRNOG00000036964 | Ralgapa2 | | 1.085 | | 0.00005 | |
| ENSRNOG00000047244 | Nup214 | | 1.083 | | 0.0008 | |
| ENSRNOG00000010625 | Dnmt3b | | 1.080 | | 0.00015 | |
| ENSRNOG00000008059 | Rnf17 | | 1.078 | | 0.00015 | |
| ENSRNOG00000023557 | RGD1563692 | | 1.070 | | 0.00005 | |
| ENSRNOG00000054371 | Prkcg | | 1.061 | | 0.00005 | |
| ENSRNOG00000056767 | Ankrd36 | | 1.047 | | 0.00715 | |
| ENSRNOG00000003643 | Tab3 | | 1.039 | | 0.00595 | |
| ENSRNOG00000021619 | Cela3b | | 1.031 | | 0.002 | |
| ENSRNOG00000013578 | Trem2 | | 1.026 | | 0.0011 | |
| ENSRNOG00000027582 | Dpysl4 | | 1.019 | | 0.00075 | |
| ENSRNOG00000017157 | Trim69 | | 1.017 | | 0.0002 | |
| ENSRNOG00000004242 | Pole2 | | 1.004 | | 0.00265 | |
| ENSRNOG00000013416 | Arid3c | | 1.002 | | 0.00005 | |

Supplementary Table 4. List of significantly downregulated genes in the AA-induced model compared to the control group

| Gene id | | Gene name | | Log_2_(Fold Change) | | p_Value |
| --- | --- | --- | --- | --- | --- | --- |
| ENSRNOG00000035103 | 5S_rRNA | | -23.188 | | 0.00145 | |
| ENSRNOG00000048176 | AABR07024593.2 | | -21.260 | | 0.00235 | |
| ENSRNOG00000054722 | U1 | | -19.970 | | 0.02395 | |
| ENSRNOG00000048073 | LOC103690821 | | -19.868 | | 0.00145 | |
| ENSRNOG00000055311 | U1 | | -19.700 | | 0.0083 | |
| ENSRNOG00000051920 | U1 | | -19.409 | | 0.00395 | |
| ENSRNOG00000032847 | RGD1561636 | | -18.434 | | 0.02395 | |
| ENSRNOG00000053409 | AABR07068852.1 | | -18.222 | | 0.00395 | |
| ENSRNOG00000028311 | Cnpy1 | | -17.673 | | 0.04905 | |
| ENSRNOG00000050226 | RGD1565894 | | -17.224 | | 0.00005 | |
| ENSRNOG00000006051 | AABR07016841.1 | | -17.206 | | 0.02395 | |
| ENSRNOG00000061507 | AABR07032328.1 | | -16.982 | | 0.02395 | |
| ENSRNOG00000032300 | Rpl39l | | -16.633 | | 0.00005 | |
| ENSRNOG00000059962 | AABR07025787.1 | | -16.152 | | 0.00145 | |
| ENSRNOG00000045790 | LOC100910130 | | -4.852 | | 0.00005 | |
| ENSRNOG00000045747 | Capns1 | | -4.097 | | 0.00005 | |
| ENSRNOG00000013141 | Eno2 | | -4.074 | | 0.00005 | |
| ENSRNOG00000051591 | LOC100911422 | | -3.974 | | 0.00005 | |
| ENSRNOG00000002462 | LOC108348055 | | -3.891 | | 0.0002 | |
| ENSRNOG00000000201 | Gsta5 | | -3.684 | | 0.00005 | |
| ENSRNOG00000008720 | LOC103692173 | | -3.622 | | 0.00005 | |
| ENSRNOG00000050492 | LOC100911807 | | -3.608 | | 0.0282 | |
| ENSRNOG00000003136 | Fcrla | | -3.342 | | 0.00005 | |
| ENSRNOG00000020781 | LOC103690005 | | -3.226 | | 0.00005 | |
| ENSRNOG00000053109 | Mrpl53 | | -3.151 | | 0.0052 | |
| ENSRNOG00000046548 | Rhox5 | | -3.097 | | 0.00005 | |
| ENSRNOG00000057515 | Arf3 | | -2.913 | | 0.0106 | |
| ENSRNOG00000028310 | S100vp | | -2.864 | | 0.0085 | |
| ENSRNOG00000001746 | Ncbp2 | | -2.748 | | 0.00005 | |
| ENSRNOG00000021555 | Mis18a | | -2.741 | | 0.00255 | |
| ENSRNOG00000042317 | Cd209f | | -2.710 | | 0.0026 | |
| ENSRNOG00000029862 | Spc24 | | -2.670 | | 0.03925 | |
| ENSRNOG00000053147 | AC141521.1 | | -2.660 | | 0.00065 | |
| ENSRNOG00000025957 | Ooep | | -2.558 | | 0.0152 | |
| ENSRNOG00000048882 | LOC100363914 | | -2.540 | | 0.00355 | |
| ENSRNOG00000003135 | Fcrlb | | -2.480 | | 0.0039 | |
| ENSRNOG00000017619 | Aldh1a1 | | -2.292 | | 0.00005 | |
| ENSRNOG00000036802 | Snhg11 | | -2.290 | | 0.00005 | |
| ENSRNOG00000004635 | Kera | | -2.260 | | 0.0006 | |
| ENSRNOG00000058424 | AABR07057150.1 | | -2.258 | | 0.0006 | |
| ENSRNOG00000029408 | Mageb16 | | -2.203 | | 0.00005 | |
| ENSRNOG00000020628 | LOC100361913 | | -2.173 | | 0.00005 | |
| ENSRNOG00000015397 | Cpne7 | | -2.141 | | 0.00005 | |
| ENSRNOG00000045778 | AC119603.1 | | -2.140 | | 0.02495 | |
| ENSRNOG00000002802 | Cxcl1 | | -2.118 | | 0.01515 | |
| ENSRNOG00000008282 | Elf5 | | -2.104 | | 0.0004 | |
| ENSRNOG00000011623 | Rab3c | | -2.100 | | 0.0003 | |
| ENSRNOG00000047647 | Il2ra | | -2.031 | | 0.00005 | |
| ENSRNOG00000049229 | LOC100911238 | | -2.021 | | 0.00005 | |
| ENSRNOG00000059883 | LOC100912578 | | -2.006 | | 0.00005 | |
| ENSRNOG00000012677 | AABR07065598.1 | | -1.989 | | 0.00825 | |
| ENSRNOG00000048898 | Wasf3 | | -1.981 | | 0.02105 | |
| ENSRNOG00000009785 | Cdkn3 | | -1.979 | | 0.0051 | |
| ENSRNOG00000055197 | AABR07027872.1 | | -1.968 | | 0.00005 | |
| ENSRNOG00000056629 | AABR07024500.1 | | -1.967 | | 0.04405 | |
| ENSRNOG00000046053 | Nudt10 | | -1.902 | | 0.00005 | |
| ENSRNOG00000028648 | Olig1 | | -1.890 | | 0.00005 | |
| ENSRNOG00000003745 | Atf3 | | -1.840 | | 0.00005 | |
| ENSRNOG00000016164 | Fcrl2 | | -1.830 | | 0.0016 | |
| ENSRNOG00000057009 | LOC103693189 | | -1.823 | | 0.0014 | |
| ENSRNOG00000010655 | Mttp | | -1.813 | | 0.00005 | |
| ENSRNOG00000023458 | Dcaf12l1 | | -1.810 | | 0.00005 | |
| ENSRNOG00000011400 | Ralyl | | -1.805 | | 0.0004 | |
| ENSRNOG00000046120 | RGD1561252 | | -1.788 | | 0.0021 | |
| ENSRNOG00000019584 | Dlk1 | | -1.750 | | 0.00225 | |
| ENSRNOG00000027139 | Krt20 | | -1.749 | | 0.00005 | |
| ENSRNOG00000010640 | Agtr1b | | -1.742 | | 0.0014 | |
| ENSRNOG00000059793 | Rab37 | | -1.741 | | 0.00005 | |
| ENSRNOG00000054340 | AABR07011013.1 | | -1.734 | | 0.0431 | |
| ENSRNOG00000060508 | AABR07028523.1 | | -1.709 | | 0.0048 | |
| ENSRNOG00000019736 | Nfs1 | | -1.709 | | 0.00005 | |
| ENSRNOG00000000582 | Ddo | | -1.698 | | 0.00225 | |
| ENSRNOG00000009530 | Uchl3 | | -1.679 | | 0.00005 | |
| ENSRNOG00000046484 | Ccdc160 | | -1.671 | | 0.0104 | |
| ENSRNOG00000021478 | Tpd52l1 | | -1.669 | | 0.01325 | |
| ENSRNOG00000054855 | AABR07014855.1 | | -1.667 | | 0.0255 | |
| ENSRNOG00000054319 | LOC103690128 | | -1.666 | | 0.0245 | |
| ENSRNOG00000003977 | Dusp1 | | -1.658 | | 0.00005 | |
| ENSRNOG00000002753 | Adam11 | | -1.658 | | 0.0186 | |
| ENSRNOG00000049743 | NEWGENE_620381 | | -1.637 | | 0.0017 | |
| ENSRNOG00000003221 | Myoc | | -1.632 | | 0.00005 | |
| ENSRNOG00000028841 | Mt1m | | -1.626 | | 0.0452 | |
| ENSRNOG00000000844 | Ly6g6d | | -1.606 | | 0.01345 | |
| ENSRNOG00000060629 | LOC103694855 | | -1.591 | | 0.00005 | |
| ENSRNOG00000010311 | Dzip1 | | -1.583 | | 0.02555 | |
| ENSRNOG00000003244 | Ltc4s | | -1.569 | | 0.00365 | |
| ENSRNOG00000029911 | Cilp | | -1.550 | | 0.00005 | |
| ENSRNOG00000018505 | Cidea | | -1.526 | | 0.018 | |
| ENSRNOG00000060105 | Astn2 | | -1.522 | | 0.03745 | |
| ENSRNOG00000002754 | Areg | | -1.502 | | 0.00005 | |
| ENSRNOG00000006867 | Etv1 | | -1.495 | | 0.00005 | |
| ENSRNOG00000011705 | Stmn2 | | -1.479 | | 0.00005 | |
| ENSRNOG00000000167 | Alas2 | | -1.478 | | 0.00015 | |
| ENSRNOG00000023182 | RGD1565317 | | -1.469 | | 0.0039 | |
| ENSRNOG00000049814 | LOC100910882 | | -1.460 | | 0.0004 | |
| ENSRNOG00000023896 | Dusp6 | | -1.458 | | 0.00005 | |
| ENSRNOG00000000969 | Cyp3a18 | | -1.456 | | 0.00005 | |
| ENSRNOG00000056219 | Olr1 | | -1.448 | | 0.00005 | |
| ENSRNOG00000046950 | Ewsr1 | | -1.442 | | 0.00005 | |
| ENSRNOG00000048771 | RGD1559482 | | -1.442 | | 0.00325 | |
| ENSRNOG00000010045 | Clec4a | | -1.426 | | 0.00095 | |
| ENSRNOG00000019776 | Sh3gl3 | | -1.405 | | 0.00005 | |
| ENSRNOG00000003756 | Lancl3 | | -1.402 | | 0.00675 | |
| ENSRNOG00000046434 | Hist1h2ail1 | | -1.396 | | 0.0223 | |
| ENSRNOG00000014940 | Sfrp5 | | -1.395 | | 0.00005 | |
| ENSRNOG00000011672 | Tph1 | | -1.395 | | 0.00005 | |
| ENSRNOG00000046858 | Arxes2 | | -1.391 | | 0.0058 | |
| ENSRNOG00000049700 | Cda | | -1.383 | | 0.02245 | |
| ENSRNOG00000048608 | Rsl1d1l1 | | -1.369 | | 0.0002 | |
| ENSRNOG00000022358 | Rn60_16_0588.3 | | -1.368 | | 0.0034 | |
| ENSRNOG00000012714 | RGD1564149 | | -1.367 | | 0.0003 | |
| ENSRNOG00000042795 | RGD1564571 | | -1.366 | | 0.0045 | |
| ENSRNOG00000049893 | LOC100910934 | | -1.364 | | 0.0001 | |
| ENSRNOG00000015498 | Il17rb | | -1.358 | | 0.0004 | |
| ENSRNOG00000015036 | Ctgf | | -1.349 | | 0.00005 | |
| ENSRNOG00000056722 | AABR07016578.3 | | -1.343 | | 0.014 | |
| ENSRNOG00000009906 | Slfnl1 | | -1.341 | | 0.00105 | |
| ENSRNOG00000002397 | Slc30a10 | | -1.340 | | 0.00005 | |
| ENSRNOG00000017019 | Lmx1b | | -1.337 | | 0.0027 | |
| ENSRNOG00000002331 | Aldh3a1 | | -1.322 | | 0.00005 | |
| ENSRNOG00000011320 | Igfbpl1 | | -1.322 | | 0.00045 | |
| ENSRNOG00000024120 | Rxfp1 | | -1.315 | | 0.00005 | |
| ENSRNOG00000000964 | AABR07036087.1 | | -1.315 | | 0.0001 | |
| ENSRNOG00000029841 | Cdh19 | | -1.301 | | 0.00005 | |
| ENSRNOG00000014378 | Il1r2 | | -1.290 | | 0.0098 | |
| ENSRNOG00000023386 | LOC103690018 | | -1.289 | | 0.00005 | |
| ENSRNOG00000011854 | RGD1563986 | | -1.279 | | 0.0021 | |
| ENSRNOG00000008890 | Slc18a2 | | -1.275 | | 0.00005 | |
| ENSRNOG00000003546 | Tnfrsf12a | | -1.274 | | 0.00005 | |
| ENSRNOG00000021424 | Cd300lf | | -1.269 | | 0.00005 | |
| ENSRNOG00000026672 | MGC94199 | | -1.259 | | 0.0017 | |
| ENSRNOG00000042524 | Milr1 | | -1.253 | | 0.00155 | |
| ENSRNOG00000011986 | Krt12 | | -1.243 | | 0.00005 | |
| ENSRNOG00000050869 | Cebpd | | -1.238 | | 0.00005 | |
| ENSRNOG00000023528 | Hrasls5 | | -1.228 | | 0.00675 | |
| ENSRNOG00000005592 | Brinp2 | | -1.220 | | 0.00005 | |
| ENSRNOG00000015088 | Prune2 | | -1.217 | | 0.00085 | |
| ENSRNOG00000011039 | Gch1 | | -1.217 | | 0.0001 | |
| ENSRNOG00000057433 | AABR07027581.1 | | -1.215 | | 0.0021 | |
| ENSRNOG00000061096 | Rn7sl1 | | -1.213 | | 0.00005 | |
| ENSRNOG00000010610 | Hpgd | | -1.209 | | 0.00005 | |
| ENSRNOG00000052899 | AABR07049886.2 | | -1.201 | | 0.0437 | |
| ENSRNOG00000000386 | Pbld1 | | -1.200 | | 0.00005 | |
| ENSRNOG00000010447 | Tmod2 | | -1.193 | | 0.00005 | |
| ENSRNOG00000002555 | Serpinb7 | | -1.193 | | 0.00005 | |
| ENSRNOG00000021945 | Ccdc184 | | -1.185 | | 0.0003 | |
| ENSRNOG00000049995 | Slc40a1 | | -1.179 | | 0.0036 | |
| ENSRNOG00000037456 | P2rx2 | | -1.176 | | 0.0006 | |
| ENSRNOG00000034191 | Fmo1 | | -1.170 | | 0.00005 | |
| ENSRNOG00000051341 | Rn50_X_0635.2 | | -1.170 | | 0.04935 | |
| ENSRNOG00000053687 | Znf660 | | -1.161 | | 0.00525 | |
| ENSRNOG00000014021 | Matn4 | | -1.159 | | 0.04035 | |
| ENSRNOG00000060602 | AABR07014275.1 | | -1.156 | | 0.00005 | |
| ENSRNOG00000004994 | Agr3 | | -1.155 | | 0.0004 | |
| ENSRNOG00000004441 | Fcmr | | -1.155 | | 0.00005 | |
| ENSRNOG00000031716 | Ecm2 | | -1.151 | | 0.00005 | |
| ENSRNOG00000060831 | Clca1 | | -1.147 | | 0.0001 | |
| ENSRNOG00000025670 | Shisa3 | | -1.136 | | 0.0001 | |
| ENSRNOG00000045989 | Hba-a3 | | -1.136 | | 0.00015 | |
| ENSRNOG00000050767 | Nrn1 | | -1.128 | | 0.0005 | |
| ENSRNOG00000011649 | Epb42 | | -1.126 | | 0.00005 | |
| ENSRNOG00000042727 | RGD1563159 | | -1.121 | | 0.02465 | |
| ENSRNOG00000009177 | Fcer1a | | -1.119 | | 0.00175 | |
| ENSRNOG00000023576 | RGD1305645 | | -1.115 | | 0.0001 | |
| ENSRNOG00000014350 | Cyr61 | | -1.112 | | 0.00005 | |
| ENSRNOG00000014227 | Mrgprx3 | | -1.107 | | 0.03025 | |
| ENSRNOG00000006231 | Ptpro | | -1.104 | | 0.00005 | |
| ENSRNOG00000053884 | Hoxa11 | | -1.103 | | 0.02165 | |
| ENSRNOG00000010063 | Htr2a | | -1.103 | | 0.0002 | |
| ENSRNOG00000016377 | Cep55 | | -1.100 | | 0.0003 | |
| ENSRNOG00000031515 | Klra2 | | -1.099 | | 0.00005 | |
| ENSRNOG00000018839 | Ntrk2 | | -1.099 | | 0.00005 | |
| ENSRNOG00000008168 | Wnt5b | | -1.097 | | 0.00005 | |
| ENSRNOG00000013312 | Kcnt2 | | -1.096 | | 0.0003 | |
| ENSRNOG00000022767 | Elfn1 | | -1.092 | | 0.00005 | |
| ENSRNOG00000037339 | Siglec10 | | -1.081 | | 0.00005 | |
| ENSRNOG00000039336 | Hrct1 | | -1.081 | | 0.0029 | |
| ENSRNOG00000008956 | Cdkn2c | | -1.081 | | 0.0003 | |
| ENSRNOG00000061883 | Aqp9 | | -1.080 | | 0.03615 | |
| ENSRNOG00000059870 | Hoxa11 | | -1.080 | | 0.0184 | |
| ENSRNOG00000000850 | Apom | | -1.080 | | 0.0046 | |
| ENSRNOG00000045760 | Ebna1bp2 | | -1.074 | | 0.00005 | |
| ENSRNOG00000003094 | Cmah | | -1.073 | | 0.00005 | |
| ENSRNOG00000054418 | AABR07005779.2 | | -1.071 | | 0.0004 | |
| ENSRNOG00000005700 | Nsg1 | | -1.069 | | 0.00005 | |
| ENSRNOG00000039759 | Gpr34 | | -1.066 | | 0.0232 | |
| ENSRNOG00000002092 | Hunk | | -1.061 | | 0.0006 | |
| ENSRNOG00000020980 | AABR07006278.1 | | -1.059 | | 0.0002 | |
| ENSRNOG00000007542 | Scg5 | | -1.058 | | 0.0007 | |
| ENSRNOG00000017557 | LOC100362216 | | -1.054 | | 0.0037 | |
| ENSRNOG00000039025 | AABR07051947.1 | | -1.054 | | 0.0132 | |
| ENSRNOG00000002524 | Gpr37 | | -1.049 | | 0.00085 | |
| ENSRNOG00000004706 | Vit | | -1.044 | | 0.0002 | |
| ENSRNOG00000017063 | Fcna | | -1.042 | | 0.00005 | |
| ENSRNOG00000015519 | Ces1d | | -1.042 | | 0.0064 | |
| ENSRNOG00000011984 | Cxcl14 | | -1.039 | | 0.00005 | |
| ENSRNOG00000055966 | Clec2d | | -1.038 | | 0.02835 | |
| ENSRNOG00000042889 | Alppl2 | | -1.037 | | 0.0009 | |
| ENSRNOG00000003872 | Slc40a1 | | -1.026 | | 0.00005 | |
| ENSRNOG00000021355 | Car6 | | -1.025 | | 0.00045 | |
| ENSRNOG00000028137 | Mki67 | | -1.024 | | 0.00005 | |
| ENSRNOG00000026295 | Rbpjl | | -1.023 | | 0.0228 | |
| ENSRNOG00000028768 | Gbp4 | | -1.019 | | 0.00165 | |
| ENSRNOG00000003218 | Zfp286a | | -1.017 | | 0.0002 | |
| ENSRNOG00000002031 | Naa11 | | -1.014 | | 0.00045 | |
| ENSRNOG00000004147 | Abca8a | | -1.014 | | 0.00005 | |
| ENSRNOG00000042070 | Ticam2 | | -1.010 | | 0.00005 | |
| ENSRNOG00000017686 | Pi15 | | -1.009 | | 0.00005 | |
| ENSRNOG00000004708 | Aard | | -1.009 | | 0.0034 | |
| ENSRNOG00000052407 | Btk | | -1.000 | | 0.00035 | |

Supplementary Table 5. List of significantly upregulated genes in the PS+LPS-induced model compared to the control group

| Gene id | | Gene name | | Log_2_(Fold Change) | | p_Value |
| --- | --- | --- | --- | --- | --- | --- |
| ENSRNOG00000055067 | 5S_rRNA | | 23.473 | | 0.00145 | |
| ENSRNOG00000060522 | Metazoa_SRP | | 22.800 | | 0.00005 | |
| ENSRNOG00000051831 | 5_8S_rRNA | | 19.662 | | 0.00145 | |
| ENSRNOG00000011557 | S100a8 | | 17.488 | | 0.00005 | |
| ENSRNOG00000060292 | NEWGENE_1587253 | | 17.463 | | 0.00005 | |
| ENSRNOG00000049123 | AABR07066510.1 | | 17.228 | | 0.02135 | |
| ENSRNOG00000054804 | AABR07017783.3 | | 16.361 | | 0.02395 | |
| ENSRNOG00000047706 | RT1-CE16 | | 6.487 | | 0.00005 | |
| ENSRNOG00000046414 | LOC108348048 | | 5.369 | | 0.0026 | |
| ENSRNOG00000054959 | Mmp11 | | 4.934 | | 0.0026 | |
| ENSRNOG00000023387 | LOC679739 | | 4.903 | | 0.00005 | |
| ENSRNOG00000054334 | Abhd10 | | 4.123 | | 0.00015 | |
| ENSRNOG00000050450 | LOC100911951 | | 3.991 | | 0.00275 | |
| ENSRNOG00000059348 | Ube2b | | 3.960 | | 0.00005 | |
| ENSRNOG00000000461 | Brd2 | | 3.740 | | 0.00005 | |
| ENSRNOG00000058083 | Metazoa_SRP | | 3.624 | | 0.00005 | |
| ENSRNOG00000061814 | Dgcr2 | | 3.407 | | 0.00005 | |
| ENSRNOG00000048016 | LOC102556337 | | 3.401 | | 0.00005 | |
| ENSRNOG00000019206 | Nupr1 | | 3.322 | | 0.00005 | |
| ENSRNOG00000045683 | LOC102553715 | | 3.316 | | 0.00005 | |
| ENSRNOG00000061096 | Rn7sl1 | | 3.238 | | 0.00005 | |
| ENSRNOG00000046379 | LOC100912604 | | 3.155 | | 0.00005 | |
| ENSRNOG00000056342 | U1 | | 3.129 | | 0.0097 | |
| ENSRNOG00000052099 | LOC103694869 | | 2.886 | | 0.00905 | |
| ENSRNOG00000050877 | Cetn3 | | 2.770 | | 0.00005 | |
| ENSRNOG00000053717 | Metazoa_SRP | | 2.730 | | 0.00005 | |
| ENSRNOG00000001167 | Ubash3a | | 2.667 | | 0.00005 | |
| ENSRNOG00000049598 | LOC100912571 | | 2.650 | | 0.00005 | |
| ENSRNOG00000009508 | Casp6 | | 2.629 | | 0.00005 | |
| ENSRNOG00000061813 | Ly6c | | 2.598 | | 0.00035 | |
| ENSRNOG00000047737 | Rpl7a | | 2.491 | | 0.00005 | |
| ENSRNOG00000001747 | Pak2 | | 2.383 | | 0.00005 | |
| ENSRNOG00000049422 | LOC108348047 | | 2.378 | | 0.00395 | |
| ENSRNOG00000046192 | Nsun4 | | 2.357 | | 0.00035 | |
| ENSRNOG00000060990 | AABR07000382.1 | | 2.343 | | 0.0004 | |
| ENSRNOG00000057527 | RNase_MRP | | 2.259 | | 0.0014 | |
| ENSRNOG00000060374 | Metazoa_SRP | | 2.194 | | 0.00005 | |
| ENSRNOG00000050509 | Ebi3 | | 2.149 | | 0.00005 | |
| ENSRNOG00000046600 | AABR07015066.1 | | 2.119 | | 0.00005 | |
| ENSRNOG00000007778 | Alox15b | | 2.007 | | 0.00015 | |
| ENSRNOG00000006620 | Xcr1 | | 1.999 | | 0.00005 | |
| ENSRNOG00000010478 | LOC500712 | | 1.990 | | 0.00005 | |
| ENSRNOG00000026310 | March1 | | 1.965 | | 0.0083 | |
| ENSRNOG00000024149 | Prr18 | | 1.936 | | 0.02685 | |
| ENSRNOG00000050000 | LOC100361706 | | 1.905 | | 0.04445 | |
| ENSRNOG00000053739 | U1 | | 1.899 | | 0.0008 | |
| ENSRNOG00000008059 | Rnf17 | | 1.851 | | 0.00005 | |
| ENSRNOG00000030246 | Btla | | 1.823 | | 0.00225 | |
| ENSRNOG00000061358 | AC129365.1 | | 1.822 | | 0.00005 | |
| ENSRNOG00000047112 | LOC100912537 | | 1.819 | | 0.0006 | |
| ENSRNOG00000060896 | AABR07063424.1 | | 1.818 | | 0.0009 | |
| ENSRNOG00000022701 | Ceacam18 | | 1.811 | | 0.0192 | |
| ENSRNOG00000021183 | LOC102552640 | | 1.808 | | 0.00005 | |
| ENSRNOG00000049575 | Atn1 | | 1.789 | | 0.00005 | |
| ENSRNOG00000051964 | LOC103691699 | | 1.785 | | 0.0012 | |
| ENSRNOG00000016444 | AABR07067506.1 | | 1.772 | | 0.00005 | |
| ENSRNOG00000059789 | Metazoa_SRP | | 1.724 | | 0.00005 | |
| ENSRNOG00000054690 | Metazoa_SRP | | 1.719 | | 0.00005 | |
| ENSRNOG00000046546 | LOC103689954 | | 1.715 | | 0.00005 | |
| ENSRNOG00000061297 | Metazoa_SRP | | 1.711 | | 0.0142 | |
| ENSRNOG00000013209 | Barhl1 | | 1.709 | | 0.00005 | |
| ENSRNOG00000016535 | Ccl22 | | 1.704 | | 0.00085 | |
| ENSRNOG00000006037 | Snap25 | | 1.688 | | 0.00005 | |
| ENSRNOG00000013578 | Trem2 | | 1.686 | | 0.00005 | |
| ENSRNOG00000047396 | Rmnd5b | | 1.674 | | 0.00005 | |
| ENSRNOG00000050395 | Ms4a6e | | 1.659 | | 0.00005 | |
| ENSRNOG00000058904 | Tex13b | | 1.643 | | 0.00005 | |
| ENSRNOG00000000239 | Ccl7 | | 1.642 | | 0.00005 | |
| ENSRNOG00000000455 | Tap2 | | 1.633 | | 0.00005 | |
| ENSRNOG00000060241 | LOC497796 | | 1.629 | | 0.00155 | |
| ENSRNOG00000006471 | Pvalb | | 1.627 | | 0.03775 | |
| ENSRNOG00000014081 | Nmur2 | | 1.597 | | 0.00005 | |
| ENSRNOG00000024310 | Kcnf1 | | 1.597 | | 0.00005 | |
| ENSRNOG00000060441 | U1 | | 1.590 | | 0.0052 | |
| ENSRNOG00000051002 | RT1-DMb | | 1.590 | | 0.0025 | |
| ENSRNOG00000017459 | C1ql3 | | 1.577 | | 0.00635 | |
| ENSRNOG00000019422 | Egr1 | | 1.575 | | 0.00005 | |
| ENSRNOG00000028436 | Rprml | | 1.558 | | 0.01535 | |
| ENSRNOG00000014350 | Cyr61 | | 1.554 | | 0.00005 | |
| ENSRNOG00000050211 | Rn50_15_0538.1 | | 1.547 | | 0.0146 | |
| ENSRNOG00000040005 | LOC108348144 | | 1.540 | | 0.00185 | |
| ENSRNOG00000052761 | AC095390.1 | | 1.539 | | 0.03655 | |
| ENSRNOG00000046246 | Spon2 | | 1.539 | | 0.01155 | |
| ENSRNOG00000058436 | LOC108348215 | | 1.535 | | 0.00005 | |
| ENSRNOG00000049878 | Sytl2 | | 1.510 | | 0.00005 | |
| ENSRNOG00000058555 | 7SK | | 1.509 | | 0.00005 | |
| ENSRNOG00000048357 | LOC103690164 | | 1.499 | | 0.00045 | |
| ENSRNOG00000015403 | Cd52 | | 1.490 | | 0.03925 | |
| ENSRNOG00000000640 | Egr2 | | 1.450 | | 0.00005 | |
| ENSRNOG00000042062 | AC105662.1 | | 1.449 | | 0.0163 | |
| ENSRNOG00000046699 | Slpi | | 1.430 | | 0.00005 | |
| ENSRNOG00000011271 | Mcc | | 1.420 | | 0.00005 | |
| ENSRNOG00000050024 | Ms4a4a | | 1.415 | | 0.00005 | |
| ENSRNOG00000049054 | Ntsr2 | | 1.407 | | 0.00005 | |
| ENSRNOG00000027582 | Dpysl4 | | 1.407 | | 0.00005 | |
| ENSRNOG00000013657 | Stmn3 | | 1.405 | | 0.02815 | |
| ENSRNOG00000048321 | Tnfsf8 | | 1.397 | | 0.00005 | |
| ENSRNOG00000046834 | C3 | | 1.379 | | 0.00005 | |
| ENSRNOG00000029394 | Dusp8 | | 1.366 | | 0.00005 | |
| ENSRNOG00000049593 | Wbp11 | | 1.365 | | 0.00005 | |
| ENSRNOG00000048161 | Tlr9 | | 1.347 | | 0.00005 | |
| ENSRNOG00000056585 | Fscn1 | | 1.312 | | 0.00005 | |
| ENSRNOG00000003348 | Rasd1 | | 1.306 | | 0.00005 | |
| ENSRNOG00000013610 | Chrna5 | | 1.297 | | 0.00005 | |
| ENSRNOG00000021731 | LOC103689949 | | 1.295 | | 0.00005 | |
| ENSRNOG00000001963 | Mx2 | | 1.290 | | 0.00005 | |
| ENSRNOG00000049491 | RT1-DMb | | 1.282 | | 0.00105 | |
| ENSRNOG00000002134 | Mpa2l | | 1.281 | | 0.00005 | |
| ENSRNOG00000032368 | Tlr11 | | 1.280 | | 0.00005 | |
| ENSRNOG00000029707 | Mt-nd4 | | 1.277 | | 0.0018 | |
| ENSRNOG00000017002 | Adrb1 | | 1.276 | | 0.00005 | |
| ENSRNOG00000024294 | AABR07019083.1 | | 1.270 | | 0.0001 | |
| ENSRNOG00000014007 | Gfod1 | | 1.269 | | 0.00005 | |
| ENSRNOG00000019661 | Gdf15 | | 1.264 | | 0.00005 | |
| ENSRNOG00000058515 | AABR07054460.4 | | 1.264 | | 0.00075 | |
| ENSRNOG00000023643 | Mmp17 | | 1.253 | | 0.00005 | |
| ENSRNOG00000008738 | Tp53i11 | | 1.234 | | 0.00005 | |
| ENSRNOG00000006853 | Elavl2 | | 1.224 | | 0.0043 | |
| ENSRNOG00000027811 | Lilrb4 | | 1.217 | | 0.0004 | |
| ENSRNOG00000001115 | Slc29a4 | | 1.216 | | 0.00055 | |
| ENSRNOG00000054141 | LOC100910915 | | 1.214 | | 0.02175 | |
| ENSRNOG00000001187 | Oasl | | 1.205 | | 0.00005 | |
| ENSRNOG00000023497 | Foxe1 | | 1.202 | | 0.00005 | |
| ENSRNOG00000043465 | Arc | | 1.199 | | 0.00005 | |
| ENSRNOG00000008475 | Fut9 | | 1.196 | | 0.00005 | |
| ENSRNOG00000022071 | Itga2b | | 1.195 | | 0.00005 | |
| ENSRNOG00000020837 | Cd300lg | | 1.192 | | 0.00125 | |
| ENSRNOG00000017786 | Acta1 | | 1.191 | | 0.00005 | |
| ENSRNOG00000051456 | AABR07013729.1 | | 1.178 | | 0.00875 | |
| ENSRNOG00000057834 | LOC102553018 | | 1.176 | | 0.0005 | |
| ENSRNOG00000027880 | Nbeal2 | | 1.172 | | 0.00005 | |
| ENSRNOG00000005370 | S1pr4 | | 1.170 | | 0.0008 | |
| ENSRNOG00000036762 | Itpripl2 | | 1.168 | | 0.00005 | |
| ENSRNOG00000010617 | Scube1 | | 1.163 | | 0.00005 | |
| ENSRNOG00000049132 | Map2k3 | | 1.163 | | 0.00005 | |
| ENSRNOG00000053699 | AC096809.1 | | 1.151 | | 0.00945 | |
| ENSRNOG00000006204 | Slc30a3 | | 1.146 | | 0.00015 | |
| ENSRNOG00000060237 | Inhbb | | 1.145 | | 0.00005 | |
| ENSRNOG00000005806 | Oxtr | | 1.143 | | 0.00375 | |
| ENSRNOG00000010906 | Ccl5 | | 1.136 | | 0.0003 | |
| ENSRNOG00000013661 | Kif26a | | 1.136 | | 0.00005 | |
| ENSRNOG00000050108 | LOC100911319 | | 1.126 | | 0.00005 | |
| ENSRNOG00000048193 | Hnrnph3 | | 1.122 | | 0.00005 | |
| ENSRNOG00000047261 | Mapre2 | | 1.118 | | 0.00055 | |
| ENSRNOG00000007159 | Ccl2 | | 1.117 | | 0.0002 | |
| ENSRNOG00000020105 | Klhl30 | | 1.116 | | 0.0001 | |
| ENSRNOG00000042838 | Junb | | 1.109 | | 0.00005 | |
| ENSRNOG00000039379 | RGD1562963 | | 1.105 | | 0.0269 | |
| ENSRNOG00000010688 | Nrgn | | 1.104 | | 0.00025 | |
| ENSRNOG00000012049 | Sox7 | | 1.101 | | 0.00005 | |
| ENSRNOG00000014231 | Pnoc | | 1.098 | | 0.00005 | |
| ENSRNOG00000030475 | Mum1l1 | | 1.096 | | 0.00005 | |
| ENSRNOG00000032639 | Foxo6 | | 1.095 | | 0.0004 | |
| ENSRNOG00000003386 | Rbfox3 | | 1.088 | | 0.00005 | |
| ENSRNOG00000047880 | LOC103689975 | | 1.086 | | 0.00025 | |
| ENSRNOG00000015668 | Ccl19 | | 1.084 | | 0.044 | |
| ENSRNOG00000006010 | RGD1566368 | | 1.083 | | 0.00015 | |
| ENSRNOG00000004578 | Cthrc1 | | 1.080 | | 0.00005 | |
| ENSRNOG00000052725 | RGD1566029 | | 1.079 | | 0.00005 | |
| ENSRNOG00000001746 | Ncbp2 | | 1.079 | | 0.00005 | |
| ENSRNOG00000017209 | Tubb3 | | 1.074 | | 0.00005 | |
| ENSRNOG00000029792 | NEWGENE_1308171 | | 1.074 | | 0.00005 | |
| ENSRNOG00000004744 | Fam84b | | 1.074 | | 0.0002 | |
| ENSRNOG00000007407 | Ndufa12 | | 1.072 | | 0.00005 | |
| ENSRNOG00000029662 | Wdfy4 | | 1.071 | | 0.00005 | |
| ENSRNOG00000047368 | Acer1 | | 1.068 | | 0.0039 | |
| ENSRNOG00000042455 | Tlr12 | | 1.063 | | 0.0003 | |
| ENSRNOG00000028440 | Apcdd1l | | 1.061 | | 0.00005 | |
| ENSRNOG00000054314 | Kcng1 | | 1.057 | | 0.00005 | |
| ENSRNOG00000058388 | Zfp36 | | 1.053 | | 0.00005 | |
| ENSRNOG00000020706 | Kcnn3 | | 1.052 | | 0.00095 | |
| ENSRNOG00000016021 | Lims2 | | 1.051 | | 0.00005 | |
| ENSRNOG00000053232 | Ror2 | | 1.050 | | 0.00005 | |
| ENSRNOG00000015366 | Neurl3 | | 1.046 | | 0.0012 | |
| ENSRNOG00000018122 | Tspan17 | | 1.044 | | 0.00005 | |
| ENSRNOG00000001892 | Tbx1 | | 1.040 | | 0.00005 | |
| ENSRNOG00000051612 | AABR07044570.1 | | 1.038 | | 0.03825 | |
| ENSRNOG00000014125 | Evi2b | | 1.037 | | 0.00165 | |
| ENSRNOG00000042414 | Nat14 | | 1.036 | | 0.00045 | |
| ENSRNOG00000001369 | Oas1a | | 1.035 | | 0.00015 | |
| ENSRNOG00000009329 | Nr1d1 | | 1.030 | | 0.00005 | |
| ENSRNOG00000006970 | Ntn3 | | 1.027 | | 0.0057 | |
| ENSRNOG00000047734 | Chst2 | | 1.024 | | 0.00005 | |
| ENSRNOG00000015554 | Ankdd1a | | 1.023 | | 0.00005 | |
| ENSRNOG00000017828 | Egr3 | | 1.023 | | 0.0051 | |
| ENSRNOG00000017899 | Akr7a3 | | 1.019 | | 0.0031 | |
| ENSRNOG00000026293 | Jun | | 1.012 | | 0.00005 | |
| ENSRNOG00000021437 | AABR07073181.1 | | 1.012 | | 0.00005 | |
| ENSRNOG00000047244 | Nup214 | | 1.011 | | 0.0012 | |
| ENSRNOG00000017531 | Akr1c3 | | 1.010 | | 0.0129 | |
| ENSRNOG00000051690 | Clec9a | | 1.007 | | 0.00015 | |
| ENSRNOG00000012477 | Eef1a2 | | 1.006 | | 0.00085 | |
| ENSRNOG00000023536 | Adgrd1 | | 1.005 | | 0.00005 | |
| ENSRNOG00000021243 | Siglec1 | | 1.003 | | 0.00005 | |

Supplementary Table 6. List of significantly downregulated genes in the PS+LPS-induced model compared to the control group

| Gene id | | Gene name | | Log_2_(Fold Change) | | p_Value |
| --- | --- | --- | --- | --- | --- | --- |
| ENSRNOG00000048176 | AABR07024593.2 | | -21.260 | | 0.00215 | |
| ENSRNOG00000048073 | LOC103690821 | | -19.868 | | 0.00135 | |
| ENSRNOG00000053109 | Mrpl53 | | -19.564 | | 0.00005 | |
| ENSRNOG00000043400 | LOC100910678 | | -19.144 | | 0.0012 | |
| ENSRNOG00000057273 | AABR07062157.1 | | -18.213 | | 0.02285 | |
| ENSRNOG00000052706 | AABR07072841.1 | | -18.132 | | 0.0058 | |
| ENSRNOG00000028311 | Cnpy1 | | -17.673 | | 0.0481 | |
| ENSRNOG00000057895 | AABR07033745.1 | | -16.764 | | 0.02285 | |
| ENSRNOG00000058218 | AC131806.3 | | -16.104 | | 0.00135 | |
| ENSRNOG00000045790 | LOC100910130 | | -5.932 | | 0.00265 | |
| ENSRNOG00000047914 | Gng11 | | -4.675 | | 0.00755 | |
| ENSRNOG00000049743 | NEWGENE_620381 | | -4.157 | | 0.01385 | |
| ENSRNOG00000020781 | LOC103690005 | | -3.962 | | 0.0002 | |
| ENSRNOG00000013953 | Ntrk1 | | -3.833 | | 0.00345 | |
| ENSRNOG00000055809 | LOC100911769 | | -3.652 | | 0.00005 | |
| ENSRNOG00000057515 | Arf3 | | -3.550 | | 0.0464 | |
| ENSRNOG00000006589 | LOC103694877 | | -3.387 | | 0.0483 | |
| ENSRNOG00000054855 | AABR07014855.1 | | -3.285 | | 0.0096 | |
| ENSRNOG00000002462 | LOC108348055 | | -3.258 | | 0.00025 | |
| ENSRNOG00000034087 | Krt42 | | -3.173 | | 0.007 | |
| ENSRNOG00000048882 | LOC100363914 | | -2.988 | | 0.00655 | |
| ENSRNOG00000052899 | AABR07049886.2 | | -2.897 | | 0.0356 | |
| ENSRNOG00000017412 | AABR07001512.1 | | -2.574 | | 0.00005 | |
| ENSRNOG00000049964 | LOC103690044 | | -2.353 | | 0.0055 | |
| ENSRNOG00000046548 | Rhox5 | | -2.343 | | 0.00005 | |
| ENSRNOG00000050855 | LOC100911994 | | -2.335 | | 0.00005 | |
| ENSRNOG00000019736 | Nfs1 | | -2.309 | | 0.00005 | |
| ENSRNOG00000000201 | Gsta5 | | -2.198 | | 0.00005 | |
| ENSRNOG00000061359 | Psme1-ps1 | | -2.186 | | 0.00005 | |
| ENSRNOG00000028803 | Cd209e | | -2.157 | | 0.02105 | |
| ENSRNOG00000052977 | LOC103690033 | | -2.090 | | 0.00005 | |
| ENSRNOG00000003136 | Fcrla | | -2.012 | | 0.00005 | |
| ENSRNOG00000062158 | Rn60_1_2212.4 | | -2.012 | | 0.00005 | |
| ENSRNOG00000008282 | Elf5 | | -1.989 | | 0.00015 | |
| ENSRNOG00000052664 | Tnks2 | | -1.984 | | 0.00005 | |
| ENSRNOG00000020749 | AC095278.1 | | -1.942 | | 0.0403 | |
| ENSRNOG00000053893 | Opn4 | | -1.936 | | 0.0152 | |
| ENSRNOG00000051672 | AABR07008030.1 | | -1.862 | | 0.0189 | |
| ENSRNOG00000048898 | Wasf3 | | -1.840 | | 0.01365 | |
| ENSRNOG00000016622 | Ankra2 | | -1.759 | | 0.00325 | |
| ENSRNOG00000048608 | Rsl1d1l1 | | -1.747 | | 0.00005 | |
| ENSRNOG00000028310 | S100vp | | -1.746 | | 0.00095 | |
| ENSRNOG00000019848 | Rbm39 | | -1.732 | | 0.00005 | |
| ENSRNOG00000037984 | Sccpdh | | -1.689 | | 0.00005 | |
| ENSRNOG00000000582 | Ddo | | -1.688 | | 0.0019 | |
| ENSRNOG00000009371 | Abl1 | | -1.681 | | 0.00005 | |
| ENSRNOG00000017820 | Nqo2 | | -1.638 | | 0.00005 | |
| ENSRNOG00000015447 | Calcoco1 | | -1.620 | | 0.00005 | |
| ENSRNOG00000009314 | Ivl | | -1.553 | | 0.00005 | |
| ENSRNOG00000047516 | Map3k7 | | -1.543 | | 0.0068 | |
| ENSRNOG00000047854 | LOC108348072 | | -1.529 | | 0.00005 | |
| ENSRNOG00000058920 | Apopt1 | | -1.506 | | 0.00005 | |
| ENSRNOG00000010730 | Efcab10 | | -1.497 | | 0.03375 | |
| ENSRNOG00000029686 | LOC102555727 | | -1.479 | | 0.00095 | |
| ENSRNOG00000002158 | Ibsp | | -1.478 | | 0.0001 | |
| ENSRNOG00000062232 | Mcc | | -1.459 | | 0.0133 | |
| ENSRNOG00000009785 | Cdkn3 | | -1.457 | | 0.0057 | |
| ENSRNOG00000042851 | AC109942.1 | | -1.454 | | 0.03585 | |
| ENSRNOG00000002754 | Areg | | -1.453 | | 0.00005 | |
| ENSRNOG00000001893 | LOC100362453 | | -1.445 | | 0.00005 | |
| ENSRNOG00000020628 | LOC100361913 | | -1.399 | | 0.00005 | |
| ENSRNOG00000045760 | Ebna1bp2 | | -1.393 | | 0.00005 | |
| ENSRNOG00000051341 | Rn50_X_0635.2 | | -1.357 | | 0.0138 | |
| ENSRNOG00000051627 | AABR07053580.1 | | -1.351 | | 0.00835 | |
| ENSRNOG00000014039 | Sucnr1 | | -1.343 | | 0.0427 | |
| ENSRNOG00000023465 | LOC500300 | | -1.341 | | 0.03025 | |
| ENSRNOG00000032350 | Kcnip4 | | -1.316 | | 0.0101 | |
| ENSRNOG00000023182 | RGD1565317 | | -1.310 | | 0.0043 | |
| ENSRNOG00000013727 | Ndc80 | | -1.309 | | 0.00055 | |
| ENSRNOG00000054806 | LOC108348323 | | -1.301 | | 0.00265 | |
| ENSRNOG00000014518 | Adam28 | | -1.282 | | 0.00005 | |
| ENSRNOG00000024365 | Ect2 | | -1.255 | | 0.002 | |
| ENSRNOG00000006365 | Asb15 | | -1.255 | | 0.0001 | |
| ENSRNOG00000003745 | Atf3 | | -1.244 | | 0.00005 | |
| ENSRNOG00000016686 | Tmem167a | | -1.243 | | 0.0425 | |
| ENSRNOG00000011892 | Slc36a2 | | -1.235 | | 0.00095 | |
| ENSRNOG00000037865 | Hormad2 | | -1.207 | | 0.00505 | |
| ENSRNOG00000038720 | Cdkl1 | | -1.182 | | 0.00005 | |
| ENSRNOG00000059776 | Tnks2 | | -1.178 | | 0.00005 | |
| ENSRNOG00000004112 | RGD1308775 | | -1.174 | | 0.00275 | |
| ENSRNOG00000033244 | LOC688473 | | -1.163 | | 0.01285 | |
| ENSRNOG00000056219 | Olr1 | | -1.162 | | 0.00005 | |
| ENSRNOG00000060831 | Clca1 | | -1.155 | | 0.0002 | |
| ENSRNOG00000003480 | Aim2 | | -1.143 | | 0.00845 | |
| ENSRNOG00000014378 | Il1r2 | | -1.140 | | 0.015 | |
| ENSRNOG00000025925 | Txk | | -1.139 | | 0.00015 | |
| ENSRNOG00000012344 | Slc7a9 | | -1.133 | | 0.0019 | |
| ENSRNOG00000004149 | Mgat4c | | -1.131 | | 0.0054 | |
| ENSRNOG00000003606 | AABR07014550.1 | | -1.125 | | 0.00005 | |
| ENSRNOG00000011649 | Epb42 | | -1.116 | | 0.00005 | |
| ENSRNOG00000060863 | AABR07017145.1 | | -1.115 | | 0.0002 | |
| ENSRNOG00000029861 | Gsta2 | | -1.111 | | 0.0229 | |
| ENSRNOG00000054665 | AABR07066861.1 | | -1.104 | | 0.00275 | |
| ENSRNOG00000056863 | RGD1562725 | | -1.094 | | 0.01705 | |
| ENSRNOG00000020641 | LOC688473 | | -1.092 | | 0.0182 | |
| ENSRNOG00000049229 | LOC100911238 | | -1.085 | | 0.00005 | |
| ENSRNOG00000005023 | Agr2 | | -1.083 | | 0.00005 | |
| ENSRNOG00000032626 | Mmp3 | | -1.081 | | 0.00005 | |
| ENSRNOG00000026112 | Tmem202 | | -1.079 | | 0.03715 | |
| ENSRNOG00000060837 | AC132752.2 | | -1.069 | | 0.02635 | |
| ENSRNOG00000054277 | Capsl | | -1.067 | | 0.00025 | |
| ENSRNOG00000013971 | Psat1 | | -1.062 | | 0.00005 | |
| ENSRNOG00000012311 | Slc35d3 | | -1.058 | | 0.00115 | |
| ENSRNOG00000042628 | RGD1561145 | | -1.058 | | 0.0017 | |
| ENSRNOG00000048088 | Mest | | -1.050 | | 0.00005 | |
| ENSRNOG00000016377 | Cep55 | | -1.037 | | 0.00075 | |
| ENSRNOG00000002970 | Zswim7 | | -1.036 | | 0.0148 | |
| ENSRNOG00000053047 | Top2a | | -1.031 | | 0.00005 | |
| ENSRNOG00000004921 | Nusap1 | | -1.024 | | 0.00145 | |
| ENSRNOG00000060629 | LOC103694855 | | -1.022 | | 0.00005 | |
| ENSRNOG00000031769 | Chchd7 | | -1.022 | | 0.0443 | |
| ENSRNOG00000031716 | Ecm2 | | -1.006 | | 0.016 | |
| ENSRNOG00000008873 | Ino80b | | -1.005 | | 0.00065 | |

Supplementary Table 7. List of significantly upregulated genes in the CYP-induced model compared to the control group

| Gene id | | Gene Name | | Log_2_(Fold Change) | | P_Value |
| --- | --- | --- | --- | --- | --- | --- |
| ENSRNOG00000050567 | LOC100911674 | | 18.560 | | 0.00005 | |
| ENSRNOG00000011557 | S100a8 | | 17.927 | | 0.00005 | |
| ENSRNOG00000050257 | AABR07003273.1 | | 17.493 | | 0.00145 | |
| ENSRNOG00000049123 | AABR07066510.1 | | 17.281 | | 0.02365 | |
| ENSRNOG00000051891 | RGD1562378 | | 17.166 | | 0.00145 | |
| ENSRNOG00000060181 | AABR07050321.2 | | 17.003 | | 0.00005 | |
| ENSRNOG00000028114 | Hbq1b | | 16.986 | | 0.00005 | |
| ENSRNOG00000016278 | Ccl17 | | 16.555 | | 0.00145 | |
| ENSRNOG00000026133 | Ly6d | | 16.028 | | 0.00005 | |
| ENSRNOG00000016361 | Plcd4 | | 5.201 | | 0.03105 | |
| ENSRNOG00000046379 | LOC100912604 | | 5.001 | | 0.00005 | |
| ENSRNOG00000048365 | LOC100911625 | | 4.491 | | 0.00005 | |
| ENSRNOG00000045683 | LOC102553715 | | 4.346 | | 0.00005 | |
| ENSRNOG00000020951 | Slc4a1 | | 4.299 | | 0.00005 | |
| ENSRNOG00000050407 | NEWGENE_1305281 | | 4.191 | | 0.00005 | |
| ENSRNOG00000045877 | Rn50_20_0060.5 | | 4.140 | | 0.00005 | |
| ENSRNOG00000003984 | Apln | | 3.993 | | 0.00005 | |
| ENSRNOG00000047396 | Rmnd5b | | 3.967 | | 0.00005 | |
| ENSRNOG00000053703 | Rn50_7_1411.1 | | 3.895 | | 0.00035 | |
| ENSRNOG00000001963 | Mx2 | | 3.799 | | 0.00005 | |
| ENSRNOG00000057527 | RNase_MRP | | 3.721 | | 0.00005 | |
| ENSRNOG00000021802 | Isg15 | | 3.719 | | 0.00005 | |
| ENSRNOG00000021018 | Batf2 | | 3.716 | | 0.00265 | |
| ENSRNOG00000009508 | Casp6 | | 3.623 | | 0.00005 | |
| ENSRNOG00000049282 | Oas2 | | 3.584 | | 0.00005 | |
| ENSRNOG00000031189 | Ido1 | | 3.336 | | 0.00005 | |
| ENSRNOG00000005277 | Ptprv | | 3.253 | | 0.00005 | |
| ENSRNOG00000001959 | Mx1 | | 3.244 | | 0.00005 | |
| ENSRNOG00000049491 | RT1-DMb | | 3.217 | | 0.00005 | |
| ENSRNOG00000047737 | Rpl7a | | 3.202 | | 0.00005 | |
| ENSRNOG00000057092 | Slfn4 | | 3.171 | | 0.00005 | |
| ENSRNOG00000001369 | Oas1a | | 3.159 | | 0.00005 | |
| ENSRNOG00000001187 | Oasl | | 3.152 | | 0.00005 | |
| ENSRNOG00000009436 | Hemgn | | 3.081 | | 0.0008 | |
| ENSRNOG00000010556 | Intu | | 3.071 | | 0.00005 | |
| ENSRNOG00000059207 | Oas3 | | 3.032 | | 0.00005 | |
| ENSRNOG00000059050 | - | | 2.953 | | 0.00005 | |
| ENSRNOG00000001747 | Pak2 | | 2.949 | | 0.00005 | |
| ENSRNOG00000007539 | Rsad2 | | 2.941 | | 0.00005 | |
| ENSRNOG00000017130 | Rhd | | 2.941 | | 0.01145 | |
| ENSRNOG00000037198 | Usp18 | | 2.928 | | 0.00005 | |
| ENSRNOG00000056585 | Fscn1 | | 2.916 | | 0.00005 | |
| ENSRNOG00000022256 | Cxcl10 | | 2.886 | | 0.00005 | |
| ENSRNOG00000012557 | Lgals5 | | 2.865 | | 0.00005 | |
| ENSRNOG00000038957 | RGD1305184 | | 2.864 | | 0.00005 | |
| ENSRNOG00000022839 | Ifit3 | | 2.786 | | 0.00005 | |
| ENSRNOG00000001527 | Cd80 | | 2.773 | | 0.0001 | |
| ENSRNOG00000002831 | Wfikkn2 | | 2.767 | | 0.00005 | |
| ENSRNOG00000059097 | Ddx60 | | 2.745 | | 0.00005 | |
| ENSRNOG00000046007 | Cldn3 | | 2.707 | | 0.0027 | |
| ENSRNOG00000024149 | Prr18 | | 2.703 | | 0.0096 | |
| ENSRNOG00000046192 | Nsun4 | | 2.674 | | 0.00015 | |
| ENSRNOG00000021256 | Adra1d | | 2.653 | | 0.00005 | |
| ENSRNOG00000017414 | Irf7 | | 2.643 | | 0.00005 | |
| ENSRNOG00000021855 | Kcp | | 2.604 | | 0.00005 | |
| ENSRNOG00000023497 | Foxe1 | | 2.539 | | 0.00005 | |
| ENSRNOG00000054334 | Abhd10 | | 2.511 | | 0.0043 | |
| ENSRNOG00000025156 | Timm17b | | 2.508 | | 0.00005 | |
| ENSRNOG00000008648 | LOC103692171 | | 2.503 | | 0.00005 | |
| ENSRNOG00000033220 | Oas1b | | 2.466 | | 0.00005 | |
| ENSRNOG00000003054 | Cask | | 2.466 | | 0.01715 | |
| ENSRNOG00000022242 | Cxcl9 | | 2.435 | | 0.00005 | |
| ENSRNOG00000061813 | Ly6c | | 2.426 | | 0.0008 | |
| ENSRNOG00000002134 | Mpa2l | | 2.420 | | 0.00005 | |
| ENSRNOG00000038894 | Timd4 | | 2.394 | | 0.00135 | |
| ENSRNOG00000015403 | Cd52 | | 2.386 | | 0.00475 | |
| ENSRNOG00000026605 | Ifi27l2b | | 2.380 | | 0.00005 | |
| ENSRNOG00000017786 | Acta1 | | 2.345 | | 0.00005 | |
| ENSRNOG00000015397 | Cpne7 | | 2.341 | | 0.00005 | |
| ENSRNOG00000054582 | LOC100134871 | | 2.326 | | 0.00005 | |
| ENSRNOG00000047076 | Oas1g | | 2.325 | | 0.00005 | |
| ENSRNOG00000007805 | Mybl2 | | 2.321 | | 0.00005 | |
| ENSRNOG00000050509 | Ebi3 | | 2.306 | | 0.00005 | |
| ENSRNOG00000048187 | LOC691153 | | 2.256 | | 0.0018 | |
| ENSRNOG00000003645 | LOC103689931 | | 2.241 | | 0.00005 | |
| ENSRNOG00000023614 | Hsh2d | | 2.241 | | 0.00005 | |
| ENSRNOG00000011445 | Nkain1 | | 2.240 | | 0.00765 | |
| ENSRNOG00000047321 | Hba-a2 | | 2.216 | | 0.00005 | |
| ENSRNOG00000028895 | Rtp4 | | 2.194 | | 0.00005 | |
| ENSRNOG00000019183 | Alox15 | | 2.189 | | 0.00005 | |
| ENSRNOG00000018247 | Dhx58 | | 2.167 | | 0.00005 | |
| ENSRNOG00000059348 | Ube2b | | 2.166 | | 0.00005 | |
| ENSRNOG00000049054 | Ntsr2 | | 2.148 | | 0.00005 | |
| ENSRNOG00000051621 | Tex40 | | 2.101 | | 0.00005 | |
| ENSRNOG00000043451 | Spp1 | | 2.099 | | 0.00005 | |
| ENSRNOG00000050453 | Fbxo27 | | 2.076 | | 0.0113 | |
| ENSRNOG00000001006 | Nptx2 | | 2.047 | | 0.00005 | |
| ENSRNOG00000049700 | Cda | | 2.044 | | 0.00005 | |
| ENSRNOG00000017897 | Adam8 | | 2.040 | | 0.00005 | |
| ENSRNOG00000045924 | RT1-T24-3 | | 2.037 | | 0.00005 | |
| ENSRNOG00000000167 | Alas2 | | 2.033 | | 0.00005 | |
| ENSRNOG00000013578 | Trem2 | | 2.020 | | 0.00005 | |
| ENSRNOG00000000777 | RT1-S3 | | 2.018 | | 0.00005 | |
| ENSRNOG00000032133 | Rnase2 | | 2.004 | | 0.01295 | |
| ENSRNOG00000011483 | S100a9 | | 2.002 | | 0.00005 | |
| ENSRNOG00000027742 | Adamtsl2 | | 2.002 | | 0.00005 | |
| ENSRNOG00000000787 | RT1-T24-2 | | 2.001 | | 0.00005 | |
| ENSRNOG00000047112 | LOC100912537 | | 2.000 | | 0.00025 | |
| ENSRNOG00000028814 | Oasl2 | | 1.992 | | 0.00005 | |
| ENSRNOG00000059225 | AABR07020987.1 | | 1.989 | | 0.0001 | |
| ENSRNOG00000022537 | E2f8 | | 1.983 | | 0.00005 | |
| ENSRNOG00000021902 | Rmi2 | | 1.964 | | 0.04975 | |
| ENSRNOG00000028436 | Rprml | | 1.946 | | 0.0043 | |
| ENSRNOG00000029886 | Hba-a2 | | 1.932 | | 0.00005 | |
| ENSRNOG00000060990 | AABR07000382.1 | | 1.923 | | 0.00195 | |
| ENSRNOG00000007778 | Alox15b | | 1.917 | | 0.0003 | |
| ENSRNOG00000021260 | Prnd | | 1.903 | | 0.00625 | |
| ENSRNOG00000026310 | March1 | | 1.893 | | 0.009 | |
| ENSRNOG00000030222 | Tcaf2 | | 1.888 | | 0.00005 | |
| ENSRNOG00000004578 | Cthrc1 | | 1.878 | | 0.00005 | |
| ENSRNOG00000013973 | Lcn2 | | 1.870 | | 0.00005 | |
| ENSRNOG00000005174 | Tmem121 | | 1.863 | | 0.00485 | |
| ENSRNOG00000008591 | Colec10 | | 1.862 | | 0.00005 | |
| ENSRNOG00000021140 | Kcnk4 | | 1.861 | | 0.00005 | |
| ENSRNOG00000018384 | Adam12 | | 1.855 | | 0.00005 | |
| ENSRNOG00000017002 | Adrb1 | | 1.853 | | 0.00005 | |
| ENSRNOG00000012686 | Pomc | | 1.852 | | 0.00675 | |
| ENSRNOG00000008759 | Csf3r | | 1.852 | | 0.00005 | |
| ENSRNOG00000058105 | Hbb | | 1.851 | | 0.00005 | |
| ENSRNOG00000000640 | Egr2 | | 1.849 | | 0.00005 | |
| ENSRNOG00000022326 | LOC103692170 | | 1.847 | | 0.00005 | |
| ENSRNOG00000047734 | Chst2 | | 1.842 | | 0.00005 | |
| ENSRNOG00000022146 | Hist1h2af | | 1.839 | | 0.002 | |
| ENSRNOG00000046246 | Spon2 | | 1.837 | | 0.0053 | |
| ENSRNOG00000005505 | Wbp11l1 | | 1.835 | | 0.00005 | |
| ENSRNOG00000009427 | Tbx21 | | 1.832 | | 0.00005 | |
| ENSRNOG00000026870 | Clic6 | | 1.829 | | 0.00005 | |
| ENSRNOG00000008225 | AABR07064349.1 | | 1.829 | | 0.04305 | |
| ENSRNOG00000024310 | Kcnf1 | | 1.829 | | 0.00005 | |
| ENSRNOG00000008012 | Abcb1a | | 1.825 | | 0.00005 | |
| ENSRNOG00000045840 | Hist1h4m | | 1.823 | | 0.00595 | |
| ENSRNOG00000006420 | Rbm38 | | 1.819 | | 0.00005 | |
| ENSRNOG00000019050 | Ifit1 | | 1.809 | | 0.00005 | |
| ENSRNOG00000059479 | Adcy1 | | 1.807 | | 0.00005 | |
| ENSRNOG00000009263 | Ifi27 | | 1.806 | | 0.00005 | |
| ENSRNOG00000030729 | C4b | | 1.805 | | 0.00005 | |
| ENSRNOG00000021437 | AABR07073181.1 | | 1.802 | | 0.00005 | |
| ENSRNOG00000013661 | Kif26a | | 1.793 | | 0.00005 | |
| ENSRNOG00000021243 | Siglec1 | | 1.773 | | 0.00005 | |
| ENSRNOG00000008040 | Fam64a | | 1.772 | | 0.0024 | |
| ENSRNOG00000057753 | Nup62cl | | 1.769 | | 0.0154 | |
| ENSRNOG00000019074 | Alox12e | | 1.768 | | 0.00005 | |
| ENSRNOG00000010649 | Ctnnd2 | | 1.765 | | 0.00005 | |
| ENSRNOG00000050211 | Rn50_15_0538.1 | | 1.764 | | 0.0085 | |
| ENSRNOG00000048295 | Dmrtc1a | | 1.761 | | 0.00795 | |
| ENSRNOG00000020025 | Slc29a2 | | 1.759 | | 0.01275 | |
| ENSRNOG00000026053 | Grem1 | | 1.746 | | 0.00005 | |
| ENSRNOG00000042062 | AC105662.1 | | 1.745 | | 0.00765 | |
| ENSRNOG00000025074 | Fgg | | 1.743 | | 0.00005 | |
| ENSRNOG00000045989 | Hba-a3 | | 1.740 | | 0.00005 | |
| ENSRNOG00000008170 | Jph2 | | 1.739 | | 0.00005 | |
| ENSRNOG00000019587 | Ptprn | | 1.723 | | 0.00005 | |
| ENSRNOG00000016148 | Gtse1 | | 1.721 | | 0.00005 | |
| ENSRNOG00000032596 | RT1-T24-1 | | 1.719 | | 0.00005 | |
| ENSRNOG00000020105 | Klhl30 | | 1.712 | | 0.00005 | |
| ENSRNOG00000060341 | AABR07031399.1 | | 1.709 | | 0.00085 | |
| ENSRNOG00000007795 | AABR07058410.1 | | 1.708 | | 0.0001 | |
| ENSRNOG00000015366 | Neurl3 | | 1.705 | | 0.00005 | |
| ENSRNOG00000049198 | Hist2h3c2 | | 1.703 | | 0.0013 | |
| ENSRNOG00000010478 | LOC500712 | | 1.702 | | 0.00005 | |
| ENSRNOG00000024846 | Ier5l | | 1.697 | | 0.00005 | |
| ENSRNOG00000053045 | Wscd2 | | 1.694 | | 0.00005 | |
| ENSRNOG00000006970 | Ntn3 | | 1.692 | | 0.00005 | |
| ENSRNOG00000015410 | Aspn | | 1.688 | | 0.00005 | |
| ENSRNOG00000016684 | Wnk2 | | 1.679 | | 0.00005 | |
| ENSRNOG00000046834 | C3 | | 1.674 | | 0.00005 | |
| ENSRNOG00000011271 | Mcc | | 1.668 | | 0.00005 | |
| ENSRNOG00000032026 | AABR07058914.1 | | 1.666 | | 0.00005 | |
| ENSRNOG00000028273 | Tmem150b | | 1.660 | | 0.0026 | |
| ENSRNOG00000053317 | Efcab11 | | 1.654 | | 0.0131 | |
| ENSRNOG00000017918 | Iglon5 | | 1.649 | | 0.0001 | |
| ENSRNOG00000042905 | RT1-T24-4 | | 1.641 | | 0.00005 | |
| ENSRNOG00000059660 | AABR07065531.5 | | 1.639 | | 0.00005 | |
| ENSRNOG00000028768 | Gbp4 | | 1.639 | | 0.00005 | |
| ENSRNOG00000052044 | AABR07054979.1 | | 1.634 | | 0.0292 | |
| ENSRNOG00000011423 | Pitx1 | | 1.629 | | 0.00005 | |
| ENSRNOG00000007514 | Sox12 | | 1.619 | | 0.00005 | |
| ENSRNOG00000023972 | Col4a2 | | 1.618 | | 0.00005 | |
| ENSRNOG00000061989 | Nkrf | | 1.615 | | 0.01255 | |
| ENSRNOG00000026508 | Vmo1 | | 1.601 | | 0.03035 | |
| ENSRNOG00000057834 | LOC102553018 | | 1.588 | | 0.00005 | |
| ENSRNOG00000023410 | Apol9a | | 1.579 | | 0.00005 | |
| ENSRNOG00000024382 | Fcgr3a | | 1.575 | | 0.0152 | |
| ENSRNOG00000006620 | Xcr1 | | 1.574 | | 0.00005 | |
| ENSRNOG00000005659 | Aurkb | | 1.572 | | 0.00005 | |
| ENSRNOG00000029141 | Trabd2b | | 1.571 | | 0.00005 | |
| ENSRNOG00000046216 | RGD1561778 | | 1.565 | | 0.04145 | |
| ENSRNOG00000007682 | Gria3 | | 1.565 | | 0.00005 | |
| ENSRNOG00000010797 | Esm1 | | 1.564 | | 0.00005 | |
| ENSRNOG00000016980 | Qprt | | 1.563 | | 0.00125 | |
| ENSRNOG00000058248 | Rn60_6_0648.1 | | 1.563 | | 0.01575 | |
| ENSRNOG00000061358 | AC129365.1 | | 1.561 | | 0.00005 | |
| ENSRNOG00000047125 | Foxl1 | | 1.556 | | 0.00005 | |
| ENSRNOG00000029394 | Dusp8 | | 1.554 | | 0.00005 | |
| ENSRNOG00000018582 | Exosc6 | | 1.551 | | 0.00005 | |
| ENSRNOG00000027894 | Iqgap3 | | 1.550 | | 0.00005 | |
| ENSRNOG00000013209 | Barhl1 | | 1.544 | | 0.0002 | |
| ENSRNOG00000053640 | Kcng2 | | 1.543 | | 0.007 | |
| ENSRNOG00000037097 | Wfdc18 | | 1.539 | | 0.0014 | |
| ENSRNOG00000008680 | Loxl1 | | 1.537 | | 0.00005 | |
| ENSRNOG00000038445 | Rnf225 | | 1.536 | | 0.00005 | |
| ENSRNOG00000022071 | Itga2b | | 1.532 | | 0.00005 | |
| ENSRNOG00000015529 | Cdca3 | | 1.531 | | 0.00015 | |
| ENSRNOG00000018822 | Slc5a5 | | 1.531 | | 0.00005 | |
| ENSRNOG00000016444 | AABR07067506.1 | | 1.526 | | 0.00005 | |
| ENSRNOG00000047880 | LOC103689975 | | 1.523 | | 0.00005 | |
| ENSRNOG00000054314 | Kcng1 | | 1.521 | | 0.00005 | |
| ENSRNOG00000047368 | Acer1 | | 1.520 | | 0.00005 | |
| ENSRNOG00000050841 | Ache | | 1.517 | | 0.00005 | |
| ENSRNOG00000036837 | Nfe2 | | 1.515 | | 0.00075 | |
| ENSRNOG00000049298 | LOC100909595 | | 1.509 | | 0.0095 | |
| ENSRNOG00000025735 | Wdr86 | | 1.506 | | 0.0093 | |
| ENSRNOG00000014956 | Slc11a1 | | 1.499 | | 0.00015 | |
| ENSRNOG00000004757 | Tmem158 | | 1.491 | | 0.00005 | |
| ENSRNOG00000022640 | Siglec8 | | 1.490 | | 0.00005 | |
| ENSRNOG00000027880 | Nbeal2 | | 1.490 | | 0.00005 | |
| ENSRNOG00000019100 | Kif2c | | 1.490 | | 0.00005 | |
| ENSRNOG00000009342 | Fcnb | | 1.489 | | 0.00115 | |
| ENSRNOG00000016281 | Col4a1 | | 1.485 | | 0.00005 | |
| ENSRNOG00000020399 | Ltb4r | | 1.484 | | 0.00005 | |
| ENSRNOG00000060514 | Wdr54 | | 1.482 | | 0.00095 | |
| ENSRNOG00000048161 | Tlr9 | | 1.479 | | 0.00005 | |
| ENSRNOG00000024294 | AABR07019083.1 | | 1.478 | | 0.00005 | |
| ENSRNOG00000001115 | Slc29a4 | | 1.472 | | 0.00005 | |
| ENSRNOG00000021713 | Kif18b | | 1.464 | | 0.0029 | |
| ENSRNOG00000021183 | LOC102552640 | | 1.464 | | 0.00105 | |
| ENSRNOG00000013862 | Dusp2 | | 1.459 | | 0.0009 | |
| ENSRNOG00000019661 | Gdf15 | | 1.459 | | 0.00005 | |
| ENSRNOG00000053232 | Ror2 | | 1.454 | | 0.00005 | |
| ENSRNOG00000032240 | Gbp5 | | 1.454 | | 0.00005 | |
| ENSRNOG00000007765 | Frzb | | 1.453 | | 0.00005 | |
| ENSRNOG00000053272 | Chi3l1 | | 1.452 | | 0.00005 | |
| ENSRNOG00000008738 | Tp53i11 | | 1.451 | | 0.00005 | |
| ENSRNOG00000023837 | Mtx3 | | 1.447 | | 0.00645 | |
| ENSRNOG00000060769 | Hist1h2bd | | 1.447 | | 0.0492 | |
| ENSRNOG00000053026 | Shcbp1 | | 1.445 | | 0.0024 | |
| ENSRNOG00000024712 | Insc | | 1.444 | | 0.0002 | |
| ENSRNOG00000007281 | Flnc | | 1.443 | | 0.00005 | |
| ENSRNOG00000017209 | Tubb3 | | 1.442 | | 0.00005 | |
| ENSRNOG00000060381 | Col15a1 | | 1.439 | | 0.00005 | |
| ENSRNOG00000032018 | Tmem200b | | 1.438 | | 0.00005 | |
| ENSRNOG00000004505 | Nfic | | 1.437 | | 0.00005 | |
| ENSRNOG00000006314 | Zbp1 | | 1.434 | | 0.00005 | |
| ENSRNOG00000053699 | AC096809.1 | | 1.433 | | 0.0025 | |
| ENSRNOG00000043300 | Enho | | 1.430 | | 0.00005 | |
| ENSRNOG00000037080 | Adamts17 | | 1.428 | | 0.00005 | |
| ENSRNOG00000008536 | Actc1 | | 1.424 | | 0.00005 | |
| ENSRNOG00000017899 | Akr7a3 | | 1.424 | | 0.0002 | |
| ENSRNOG00000051002 | RT1-DMb | | 1.421 | | 0.00555 | |
| ENSRNOG00000017463 | Bloc1s3 | | 1.412 | | 0.00005 | |
| ENSRNOG00000018458 | Ncr1 | | 1.410 | | 0.00045 | |
| ENSRNOG00000042960 | Rgcc | | 1.410 | | 0.00005 | |
| ENSRNOG00000013548 | Selenow | | 1.408 | | 0.00005 | |
| ENSRNOG00000048636 | Il2rb | | 1.407 | | 0.00005 | |
| ENSRNOG00000010402 | Hspb2 | | 1.406 | | 0.02525 | |
| ENSRNOG00000027489 | Mn1 | | 1.406 | | 0.00005 | |
| ENSRNOG00000033893 | Cacna1h | | 1.404 | | 0.00005 | |
| ENSRNOG00000059260 | AABR07053509.2 | | 1.403 | | 0.00005 | |
| ENSRNOG00000055426 | Rab42 | | 1.403 | | 0.0001 | |
| ENSRNOG00000019958 | Tmem151b | | 1.402 | | 0.00175 | |
| ENSRNOG00000021021 | Ffar2 | | 1.401 | | 0.015 | |
| ENSRNOG00000010600 | Cysrt1 | | 1.399 | | 0.00005 | |
| ENSRNOG00000046699 | Slpi | | 1.392 | | 0.00005 | |
| ENSRNOG00000051372 | Mycn | | 1.392 | | 0.0001 | |
| ENSRNOG00000050108 | LOC100911319 | | 1.391 | | 0.00005 | |
| ENSRNOG00000008296 | Tpgs1 | | 1.390 | | 0.00005 | |
| ENSRNOG00000036677 | Slc16a3 | | 1.389 | | 0.00005 | |
| ENSRNOG00000025075 | Relt | | 1.384 | | 0.00035 | |
| ENSRNOG00000000500 | Scube3 | | 1.378 | | 0.00005 | |
| ENSRNOG00000014182 | Tns1 | | 1.372 | | 0.00005 | |
| ENSRNOG00000016164 | Fcrl2 | | 1.372 | | 0.01285 | |
| ENSRNOG00000052725 | RGD1566029 | | 1.369 | | 0.00005 | |
| ENSRNOG00000057505 | AABR07015015.2 | | 1.367 | | 0.043 | |
| ENSRNOG00000007607 | Nr4a1 | | 1.366 | | 0.00005 | |
| ENSRNOG00000015858 | Hyal1 | | 1.365 | | 0.00005 | |
| ENSRNOG00000017684 | Fbxl22 | | 1.362 | | 0.0396 | |
| ENSRNOG00000013267 | Helz2 | | 1.356 | | 0.00005 | |
| ENSRNOG00000053502 | Arhgef17 | | 1.355 | | 0.00005 | |
| ENSRNOG00000008570 | Cadps | | 1.353 | | 0.00005 | |
| ENSRNOG00000034102 | Plk5 | | 1.351 | | 0.00005 | |
| ENSRNOG00000027582 | Dpysl4 | | 1.350 | | 0.00005 | |
| ENSRNOG00000048357 | LOC103690164 | | 1.349 | | 0.0314 | |
| ENSRNOG00000016141 | Hoxc11 | | 1.347 | | 0.03485 | |
| ENSRNOG00000034200 | Atp8a1 | | 1.346 | | 0.00005 | |
| ENSRNOG00000048053 | Rn50_10_0701.2 | | 1.345 | | 0.00005 | |
| ENSRNOG00000050714 | Islr2 | | 1.344 | | 0.00005 | |
| ENSRNOG00000018824 | Slc7a5 | | 1.344 | | 0.00005 | |
| ENSRNOG00000059373 | M6pr | | 1.342 | | 0.00005 | |
| ENSRNOG00000015668 | Ccl19 | | 1.341 | | 0.01565 | |
| ENSRNOG00000002568 | Socs1 | | 1.340 | | 0.00005 | |
| ENSRNOG00000014987 | Mdfi | | 1.339 | | 0.00005 | |
| ENSRNOG00000060041 | AABR07002677.2 | | 1.339 | | 0.00005 | |
| ENSRNOG00000054286 | Rrm2 | | 1.338 | | 0.00145 | |
| ENSRNOG00000029401 | Actg2 | | 1.337 | | 0.00005 | |
| ENSRNOG00000002582 | Hand1 | | 1.336 | | 0.0077 | |
| ENSRNOG00000017980 | Itgal | | 1.335 | | 0.00005 | |
| ENSRNOG00000061299 | LOC103694857 | | 1.333 | | 0.00005 | |
| ENSRNOG00000007354 | Trpa1 | | 1.328 | | 0.00005 | |
| ENSRNOG00000016274 | Zfp580 | | 1.328 | | 0.00005 | |
| ENSRNOG00000053675 | Dhh | | 1.328 | | 0.0004 | |
| ENSRNOG00000050024 | Ms4a4a | | 1.326 | | 0.00005 | |
| ENSRNOG00000000457 | Tap1 | | 1.324 | | 0.00005 | |
| ENSRNOG00000009173 | Smad6 | | 1.322 | | 0.00005 | |
| ENSRNOG00000023411 | Vsig10l | | 1.322 | | 0.00005 | |
| ENSRNOG00000017531 | Akr1c3 | | 1.320 | | 0.00205 | |
| ENSRNOG00000018326 | Pgls | | 1.320 | | 0.00005 | |
| ENSRNOG00000007811 | Klrb1c | | 1.314 | | 0.02 | |
| ENSRNOG00000048411 | Uhrf1 | | 1.312 | | 0.00005 | |
| ENSRNOG00000017365 | Klhl35 | | 1.310 | | 0.00115 | |
| ENSRNOG00000012681 | Lgals9 | | 1.309 | | 0.00005 | |
| ENSRNOG00000008625 | Rimkla | | 1.305 | | 0.01155 | |
| ENSRNOG00000012318 | Aspm | | 1.304 | | 0.00005 | |
| ENSRNOG00000048907 | AABR07010563.1 | | 1.300 | | 0.00005 | |
| ENSRNOG00000003386 | Rbfox3 | | 1.300 | | 0.00005 | |
| ENSRNOG00000046295 | Ubald1 | | 1.299 | | 0.00005 | |
| ENSRNOG00000012991 | Adgra2 | | 1.299 | | 0.00005 | |
| ENSRNOG00000045843 | Rusc2 | | 1.298 | | 0.00005 | |
| ENSRNOG00000048897 | Vmac | | 1.295 | | 0.00005 | |
| ENSRNOG00000046500 | Irgq | | 1.293 | | 0.00005 | |
| ENSRNOG00000014847 | Rassf10 | | 1.289 | | 0.00015 | |
| ENSRNOG00000021063 | Grin2d | | 1.288 | | 0.00005 | |
| ENSRNOG00000012843 | Aspg | | 1.286 | | 0.00005 | |
| ENSRNOG00000011063 | Dennd1b | | 1.281 | | 0.0015 | |
| ENSRNOG00000029843 | LOC100359563 | | 1.281 | | 0.04755 | |
| ENSRNOG00000000455 | Tap2 | | 1.278 | | 0.0011 | |
| ENSRNOG00000019207 | Shank1 | | 1.278 | | 0.0227 | |
| ENSRNOG00000048651 | Nrtn | | 1.276 | | 0.00005 | |
| ENSRNOG00000042182 | Mroh2a | | 1.274 | | 0.00005 | |
| ENSRNOG00000020676 | Ppp1r14a | | 1.271 | | 0.00005 | |
| ENSRNOG00000011381 | Acsbg1 | | 1.271 | | 0.00005 | |
| ENSRNOG00000005569 | Phospho1 | | 1.271 | | 0.00005 | |
| ENSRNOG00000046546 | LOC103689954 | | 1.270 | | 0.0003 | |
| ENSRNOG00000014008 | Mfsd2a | | 1.264 | | 0.0034 | |
| ENSRNOG00000006198 | Prr11 | | 1.264 | | 0.00155 | |
| ENSRNOG00000053240 | Soga1 | | 1.263 | | 0.00005 | |
| ENSRNOG00000047261 | Mapre2 | | 1.263 | | 0.00015 | |
| ENSRNOG00000048264 | LOC684828 | | 1.262 | | 0.00015 | |
| ENSRNOG00000052256 | Dact3 | | 1.262 | | 0.00005 | |
| ENSRNOG00000042455 | Tlr12 | | 1.260 | | 0.00005 | |
| ENSRNOG00000039858 | Mfsd12 | | 1.260 | | 0.00005 | |
| ENSRNOG00000012286 | Il20ra | | 1.259 | | 0.00005 | |
| ENSRNOG00000020009 | Npas4 | | 1.259 | | 0.0001 | |
| ENSRNOG00000036598 | LOC685933 | | 1.258 | | 0.00325 | |
| ENSRNOG00000039587 | Wdr13 | | 1.255 | | 0.00005 | |
| ENSRNOG00000030689 | Ms4a6bl | | 1.254 | | 0.01135 | |
| ENSRNOG00000061895 | Ly49si1 | | 1.254 | | 0.00005 | |
| ENSRNOG00000031743 | Gbp2 | | 1.251 | | 0.00005 | |
| ENSRNOG00000036762 | Itpripl2 | | 1.251 | | 0.00005 | |
| ENSRNOG00000032575 | Ocel1 | | 1.251 | | 0.00005 | |
| ENSRNOG00000010107 | Palld | | 1.250 | | 0.00005 | |
| ENSRNOG00000016156 | Nptxr | | 1.250 | | 0.00005 | |
| ENSRNOG00000049661 | Inafm1 | | 1.249 | | 0.00005 | |
| ENSRNOG00000028390 | Hhipl1 | | 1.249 | | 0.00005 | |
| ENSRNOG00000020839 | Lrfn3 | | 1.242 | | 0.00005 | |
| ENSRNOG00000018509 | Cx3cr1 | | 1.240 | | 0.00005 | |
| ENSRNOG00000022934 | RGD1562399 | | 1.239 | | 0.00005 | |
| ENSRNOG00000015308 | Pbk | | 1.238 | | 0.0002 | |
| ENSRNOG00000030830 | Mex3d | | 1.238 | | 0.00005 | |
| ENSRNOG00000011263 | Plac9 | | 1.237 | | 0.00005 | |
| ENSRNOG00000014170 | Dbn1 | | 1.237 | | 0.00005 | |
| ENSRNOG00000009113 | Marcksl1 | | 1.236 | | 0.00005 | |
| ENSRNOG00000000443 | C4a | | 1.233 | | 0.0027 | |
| ENSRNOG00000023969 | Herc6 | | 1.232 | | 0.00005 | |
| ENSRNOG00000000528 | Fgd2 | | 1.231 | | 0.00005 | |
| ENSRNOG00000011927 | Sdc3 | | 1.230 | | 0.00005 | |
| ENSRNOG00000021039 | Fam83e | | 1.229 | | 0.00005 | |
| ENSRNOG00000012067 | Fam111a | | 1.228 | | 0.00005 | |
| ENSRNOG00000021891 | Zdhhc8 | | 1.227 | | 0.00005 | |
| ENSRNOG00000055447 | AC111292.2 | | 1.224 | | 0.04555 | |
| ENSRNOG00000019810 | Des | | 1.223 | | 0.00005 | |
| ENSRNOG00000019418 | Lrrc4b | | 1.223 | | 0.00005 | |
| ENSRNOG00000058436 | LOC108348215 | | 1.222 | | 0.00005 | |
| ENSRNOG00000017020 | Inpp5d | | 1.222 | | 0.00005 | |
| ENSRNOG00000032908 | Acaa1b | | 1.221 | | 0.00005 | |
| ENSRNOG00000056767 | Ankrd36 | | 1.220 | | 0.0014 | |
| ENSRNOG00000046660 | Gpc6 | | 1.219 | | 0.00005 | |
| ENSRNOG00000001229 | Col18a1 | | 1.216 | | 0.00005 | |
| ENSRNOG00000031778 | Mef2d | | 1.215 | | 0.00005 | |
| ENSRNOG00000009872 | Kcnh2 | | 1.215 | | 0.00005 | |
| ENSRNOG00000054560 | Sh2d3c | | 1.214 | | 0.00005 | |
| ENSRNOG00000008450 | LOC100359539 | | 1.212 | | 0.00005 | |
| ENSRNOG00000002229 | Adcy5 | | 1.212 | | 0.00005 | |
| ENSRNOG00000001311 | Rab36 | | 1.212 | | 0.00745 | |
| ENSRNOG00000017459 | C1ql3 | | 1.211 | | 0.02145 | |
| ENSRNOG00000049880 | Tmem200c | | 1.211 | | 0.00005 | |
| ENSRNOG00000014672 | Hs3st6 | | 1.210 | | 0.00005 | |
| ENSRNOG00000020165 | Ahsp | | 1.209 | | 0.0058 | |
| ENSRNOG00000048004 | Garem2 | | 1.208 | | 0.00025 | |
| ENSRNOG00000006037 | Snap25 | | 1.208 | | 0.00005 | |
| ENSRNOG00000006527 | Slc6a1 | | 1.208 | | 0.00025 | |
| ENSRNOG00000008182 | Htra3 | | 1.207 | | 0.00005 | |
| ENSRNOG00000015304 | Tmem160 | | 1.204 | | 0.00005 | |
| ENSRNOG00000014461 | Galns | | 1.202 | | 0.00005 | |
| ENSRNOG00000055936 | Trnp1 | | 1.202 | | 0.02365 | |
| ENSRNOG00000004861 | Itga4 | | 1.199 | | 0.0002 | |
| ENSRNOG00000056153 | Fam46b | | 1.199 | | 0.00005 | |
| ENSRNOG00000023931 | Ggn | | 1.197 | | 0.00005 | |
| ENSRNOG00000056678 | Nckap5l | | 1.196 | | 0.00005 | |
| ENSRNOG00000037476 | Galnt9 | | 1.194 | | 0.0014 | |
| ENSRNOG00000018338 | Vwa1 | | 1.193 | | 0.00005 | |
| ENSRNOG00000014007 | Gfod1 | | 1.189 | | 0.00005 | |
| ENSRNOG00000050485 | Gas1 | | 1.189 | | 0.00005 | |
| ENSRNOG00000016021 | Lims2 | | 1.189 | | 0.00005 | |
| ENSRNOG00000007426 | Tmem64 | | 1.188 | | 0.00005 | |
| ENSRNOG00000008246 | Emilin1 | | 1.187 | | 0.00005 | |
| ENSRNOG00000026163 | Cpt1c | | 1.186 | | 0.00005 | |
| ENSRNOG00000055241 | AABR07053500.1 | | 1.186 | | 0.01335 | |
| ENSRNOG00000000033 | Tmcc2 | | 1.184 | | 0.00005 | |
| ENSRNOG00000016622 | Ankra2 | | 1.183 | | 0.0005 | |
| ENSRNOG00000006731 | Spc25 | | 1.182 | | 0.00685 | |
| ENSRNOG00000032639 | Foxo6 | | 1.181 | | 0.0002 | |
| ENSRNOG00000001302 | Adora2a | | 1.181 | | 0.00005 | |
| ENSRNOG00000000563 | Adamts14 | | 1.178 | | 0.00005 | |
| ENSRNOG00000045543 | Frat2 | | 1.177 | | 0.01815 | |
| ENSRNOG00000060898 | LOC100910418 | | 1.177 | | 0.0164 | |
| ENSRNOG00000050964 | LOC100911068 | | 1.177 | | 0.00005 | |
| ENSRNOG00000060435 | Kcnq4 | | 1.173 | | 0.00005 | |
| ENSRNOG00000042041 | Gal3st1 | | 1.173 | | 0.00125 | |
| ENSRNOG00000002950 | Lyl1 | | 1.172 | | 0.00005 | |
| ENSRNOG00000025350 | Ppp1r13l | | 1.169 | | 0.00005 | |
| ENSRNOG00000018815 | Plk1 | | 1.169 | | 0.00005 | |
| ENSRNOG00000039284 | Haus4 | | 1.168 | | 0.00005 | |
| ENSRNOG00000009790 | Kcnk3 | | 1.168 | | 0.00005 | |
| ENSRNOG00000017606 | P2rx1 | | 1.166 | | 0.00005 | |
| ENSRNOG00000046560 | AC109096.1 | | 1.164 | | 0.01925 | |
| ENSRNOG00000059618 | Scarf1 | | 1.164 | | 0.00005 | |
| ENSRNOG00000051456 | AABR07013729.1 | | 1.162 | | 0.0098 | |
| ENSRNOG00000032231 | Mroh6 | | 1.160 | | 0.00005 | |
| ENSRNOG00000059500 | Cdkn1c | | 1.159 | | 0.00005 | |
| ENSRNOG00000060241 | LOC497796 | | 1.159 | | 0.01625 | |
| ENSRNOG00000059900 | Bst2 | | 1.158 | | 0.00005 | |
| ENSRNOG00000008215 | Trim47 | | 1.158 | | 0.00005 | |
| ENSRNOG00000006548 | Mrc2 | | 1.157 | | 0.00005 | |
| ENSRNOG00000010906 | Ccl5 | | 1.155 | | 0.0003 | |
| ENSRNOG00000033736 | Diras3 | | 1.154 | | 0.00005 | |
| ENSRNOG00000047714 | Tmem37 | | 1.150 | | 0.0001 | |
| ENSRNOG00000027736 | Cnn1 | | 1.150 | | 0.00005 | |
| ENSRNOG00000022030 | Aunip | | 1.149 | | 0.0147 | |
| ENSRNOG00000029191 | LOC685067 | | 1.147 | | 0.00005 | |
| ENSRNOG00000001492 | Slc8a2 | | 1.147 | | 0.00005 | |
| ENSRNOG00000010718 | Gpr153 | | 1.147 | | 0.00005 | |
| ENSRNOG00000057335 | Clec1b | | 1.147 | | 0.04925 | |
| ENSRNOG00000006278 | Tspyl5 | | 1.146 | | 0.0004 | |
| ENSRNOG00000015421 | Slc27a3 | | 1.145 | | 0.00005 | |
| ENSRNOG00000008056 | Ankrd9 | | 1.145 | | 0.00005 | |
| ENSRNOG00000039339 | Mslnl | | 1.144 | | 0.0008 | |
| ENSRNOG00000018226 | Zcchc14 | | 1.144 | | 0.00005 | |
| ENSRNOG00000051915 | Spred3 | | 1.143 | | 0.00005 | |
| ENSRNOG00000028440 | Apcdd1l | | 1.142 | | 0.00005 | |
| ENSRNOG00000029862 | Spc24 | | 1.140 | | 0.00765 | |
| ENSRNOG00000012280 | Ptx3 | | 1.140 | | 0.02925 | |
| ENSRNOG00000010325 | Ptger3 | | 1.137 | | 0.00005 | |
| ENSRNOG00000000288 | Scarf2 | | 1.137 | | 0.00005 | |
| ENSRNOG00000008749 | Col5a1 | | 1.136 | | 0.00005 | |
| ENSRNOG00000006952 | Prex1 | | 1.134 | | 0.00005 | |
| ENSRNOG00000003947 | Ntn1 | | 1.134 | | 0.00005 | |
| ENSRNOG00000020281 | Kif22 | | 1.134 | | 0.00005 | |
| ENSRNOG00000053828 | Ppp1r12c | | 1.131 | | 0.00005 | |
| ENSRNOG00000016671 | Dtna | | 1.130 | | 0.00005 | |
| ENSRNOG00000032368 | Tlr11 | | 1.129 | | 0.0001 | |
| ENSRNOG00000003298 | Cd247 | | 1.128 | | 0.00275 | |
| ENSRNOG00000019584 | Dlk1 | | 1.128 | | 0.00065 | |
| ENSRNOG00000000239 | Ccl7 | | 1.127 | | 0.00095 | |
| ENSRNOG00000019869 | Lrfn1 | | 1.127 | | 0.0002 | |
| ENSRNOG00000021174 | Macrod1 | | 1.127 | | 0.0018 | |
| ENSRNOG00000003388 | Cenpf | | 1.127 | | 0.00005 | |
| ENSRNOG00000027811 | Lilrb4 | | 1.125 | | 0.0009 | |
| ENSRNOG00000000639 | Ado | | 1.124 | | 0.00005 | |
| ENSRNOG00000021663 | RGD1561849 | | 1.123 | | 0.00005 | |
| ENSRNOG00000010617 | Scube1 | | 1.123 | | 0.00005 | |
| ENSRNOG00000048222 | Nlrc5 | | 1.123 | | 0.00005 | |
| ENSRNOG00000009787 | Efcc1 | | 1.122 | | 0.0001 | |
| ENSRNOG00000007118 | Eva1a | | 1.121 | | 0.00005 | |
| ENSRNOG00000030486 | Prdm6 | | 1.121 | | 0.00005 | |
| ENSRNOG00000007964 | Tp53inp1 | | 1.121 | | 0.00005 | |
| ENSRNOG00000001729 | Xxylt1 | | 1.121 | | 0.00005 | |
| ENSRNOG00000048248 | LOC100910446 | | 1.120 | | 0.02395 | |
| ENSRNOG00000019568 | Jund | | 1.119 | | 0.00005 | |
| ENSRNOG00000047367 | Card14 | | 1.119 | | 0.00005 | |
| ENSRNOG00000008586 | Aldh1l2 | | 1.119 | | 0.00005 | |
| ENSRNOG00000062252 | Hmcn2 | | 1.118 | | 0.00005 | |
| ENSRNOG00000020038 | Chpf | | 1.116 | | 0.00005 | |
| ENSRNOG00000046377 | Btnl9 | | 1.116 | | 0.0001 | |
| ENSRNOG00000002456 | Hlf | | 1.116 | | 0.00585 | |
| ENSRNOG00000021338 | Tmem132a | | 1.115 | | 0.00005 | |
| ENSRNOG00000021003 | Sac3d1 | | 1.115 | | 0.00005 | |
| ENSRNOG00000007091 | Ly6e | | 1.115 | | 0.00005 | |
| ENSRNOG00000023536 | Adgrd1 | | 1.114 | | 0.00005 | |
| ENSRNOG00000017560 | Mdk | | 1.114 | | 0.00005 | |
| ENSRNOG00000020922 | Hspb6 | | 1.113 | | 0.00005 | |
| ENSRNOG00000016617 | Wwtr1 | | 1.112 | | 0.00005 | |
| ENSRNOG00000053691 | Lama5 | | 1.111 | | 0.00005 | |
| ENSRNOG00000016221 | Scn2b | | 1.111 | | 0.00005 | |
| ENSRNOG00000001892 | Tbx1 | | 1.111 | | 0.00005 | |
| ENSRNOG00000020455 | Cst6 | | 1.107 | | 0.00005 | |
| ENSRNOG00000014230 | Map1a | | 1.107 | | 0.00005 | |
| ENSRNOG00000030530 | Gzmm | | 1.107 | | 0.0007 | |
| ENSRNOG00000052444 | RGD1563091 | | 1.106 | | 0.00005 | |
| ENSRNOG00000003280 | Grin2c | | 1.106 | | 0.00005 | |
| ENSRNOG00000015896 | Rbpms2 | | 1.104 | | 0.00005 | |
| ENSRNOG00000060703 | Troap | | 1.104 | | 0.00145 | |
| ENSRNOG00000020353 | Sh3pxd2a | | 1.104 | | 0.00005 | |
| ENSRNOG00000025037 | Ankk1 | | 1.103 | | 0.00005 | |
| ENSRNOG00000011718 | C1rl | | 1.102 | | 0.00005 | |
| ENSRNOG00000016108 | Phlpp2 | | 1.101 | | 0.00005 | |
| ENSRNOG00000020784 | Kcnk7 | | 1.101 | | 0.00005 | |
| ENSRNOG00000001031 | Ocm2 | | 1.100 | | 0.00015 | |
| ENSRNOG00000010489 | Samd4a | | 1.099 | | 0.00005 | |
| ENSRNOG00000019698 | Ssbp4 | | 1.099 | | 0.00005 | |
| ENSRNOG00000030920 | Rtn4r | | 1.098 | | 0.00005 | |
| ENSRNOG00000003657 | Pkmyt1 | | 1.097 | | 0.00035 | |
| ENSRNOG00000055650 | Pou2f2 | | 1.095 | | 0.0002 | |
| ENSRNOG00000023643 | Mmp17 | | 1.092 | | 0.0001 | |
| ENSRNOG00000033588 | Siglec15 | | 1.091 | | 0.0001 | |
| ENSRNOG00000022421 | Crtc1 | | 1.089 | | 0.00005 | |
| ENSRNOG00000000521 | Cdkn1a | | 1.089 | | 0.00005 | |
| ENSRNOG00000026518 | Tmem156 | | 1.089 | | 0.00585 | |
| ENSRNOG00000013663 | Tmem86a | | 1.088 | | 0.00005 | |
| ENSRNOG00000002922 | Adora2b | | 1.086 | | 0.00005 | |
| ENSRNOG00000020867 | Numbl | | 1.085 | | 0.00005 | |
| ENSRNOG00000003066 | Wnt9a | | 1.082 | | 0.00055 | |
| ENSRNOG00000021244 | Hspa12b | | 1.082 | | 0.00005 | |
| ENSRNOG00000039079 | Ces2h | | 1.082 | | 0.00005 | |
| ENSRNOG00000004155 | Samd14 | | 1.081 | | 0.0042 | |
| ENSRNOG00000049828 | Crlf2 | | 1.080 | | 0.00005 | |
| ENSRNOG00000059140 | Myo1g | | 1.078 | | 0.00005 | |
| ENSRNOG00000017084 | Hsd11b2 | | 1.077 | | 0.00005 | |
| ENSRNOG00000010119 | Zmat3 | | 1.077 | | 0.0001 | |
| ENSRNOG00000010880 | Gpr27 | | 1.077 | | 0.0001 | |
| ENSRNOG00000001057 | Ctxn1 | | 1.077 | | 0.00005 | |
| ENSRNOG00000014375 | Adgrb2 | | 1.077 | | 0.00005 | |
| ENSRNOG00000012303 | Apobec2 | | 1.076 | | 0.00005 | |
| ENSRNOG00000028350 | Arse | | 1.073 | | 0.00005 | |
| ENSRNOG00000004405 | Pigr | | 1.073 | | 0.00005 | |
| ENSRNOG00000033741 | Ankrd34a | | 1.071 | | 0.00065 | |
| ENSRNOG00000003895 | Rgs1 | | 1.069 | | 0.00005 | |
| ENSRNOG00000049575 | Atn1 | | 1.069 | | 0.00005 | |
| ENSRNOG00000029195 | Uba7 | | 1.067 | | 0.00005 | |
| ENSRNOG00000050430 | Vav1 | | 1.064 | | 0.00005 | |
| ENSRNOG00000020339 | Neurl1 | | 1.064 | | 0.00095 | |
| ENSRNOG00000019478 | Irf9 | | 1.063 | | 0.00005 | |
| ENSRNOG00000003217 | Lgals3bp | | 1.063 | | 0.00005 | |
| ENSRNOG00000012442 | Cemip | | 1.063 | | 0.00005 | |
| ENSRNOG00000000536 | Mdga1 | | 1.062 | | 0.00055 | |
| ENSRNOG00000014328 | Frs3 | | 1.062 | | 0.00005 | |
| ENSRNOG00000050979 | Ubap1l | | 1.062 | | 0.01095 | |
| ENSRNOG00000013218 | Sap30 | | 1.061 | | 0.00235 | |
| ENSRNOG00000040005 | LOC108348144 | | 1.061 | | 0.0209 | |
| ENSRNOG00000049287 | MGC94199 | | 1.060 | | 0.0105 | |
| ENSRNOG00000024641 | LOC100910838 | | 1.060 | | 0.00005 | |
| ENSRNOG00000021540 | RGD1563441 | | 1.060 | | 0.0007 | |
| ENSRNOG00000007483 | Ccnf | | 1.060 | | 0.00005 | |
| ENSRNOG00000004744 | Fam84b | | 1.060 | | 0.0002 | |
| ENSRNOG00000004169 | Fzr1 | | 1.058 | | 0.00005 | |
| ENSRNOG00000051285 | Rn50_8_0651.1 | | 1.058 | | 0.00005 | |
| ENSRNOG00000042915 | Mxra7 | | 1.057 | | 0.00005 | |
| ENSRNOG00000005535 | Ikzf4 | | 1.057 | | 0.00015 | |
| ENSRNOG00000031167 | Srxn1 | | 1.057 | | 0.00025 | |
| ENSRNOG00000004703 | Muc15 | | 1.057 | | 0.0047 | |
| ENSRNOG00000004022 | Klhdc9 | | 1.053 | | 0.01425 | |
| ENSRNOG00000004476 | Wif1 | | 1.052 | | 0.00005 | |
| ENSRNOG00000036604 | Ifit2 | | 1.051 | | 0.00005 | |
| ENSRNOG00000015085 | Dmpk | | 1.051 | | 0.00005 | |
| ENSRNOG00000018122 | Tspan17 | | 1.051 | | 0.00005 | |
| ENSRNOG00000019987 | Scand1 | | 1.049 | | 0.00005 | |
| ENSRNOG00000047247 | Ptprs | | 1.049 | | 0.00005 | |
| ENSRNOG00000009468 | Il12a | | 1.049 | | 0.0001 | |
| ENSRNOG00000042059 | Rccd1 | | 1.048 | | 0.0004 | |
| ENSRNOG00000010802 | Ube3d | | 1.048 | | 0.00005 | |
| ENSRNOG00000033921 | Als2cl | | 1.048 | | 0.00005 | |
| ENSRNOG00000019692 | Metrn | | 1.047 | | 0.00025 | |
| ENSRNOG00000010688 | Nrgn | | 1.046 | | 0.0003 | |
| ENSRNOG00000026408 | Rnf169 | | 1.045 | | 0.00025 | |
| ENSRNOG00000003305 | Cxcr3 | | 1.045 | | 0.02035 | |
| ENSRNOG00000023828 | LOC680875 | | 1.045 | | 0.00005 | |
| ENSRNOG00000015664 | Tmem8b | | 1.044 | | 0.00005 | |
| ENSRNOG00000036680 | Notum | | 1.043 | | 0.00005 | |
| ENSRNOG00000020706 | Kcnn3 | | 1.042 | | 0.00095 | |
| ENSRNOG00000014786 | Ccne1 | | 1.041 | | 0.00005 | |
| ENSRNOG00000033496 | Igdcc4 | | 1.041 | | 0.0001 | |
| ENSRNOG00000004094 | Ptger1 | | 1.041 | | 0.00005 | |
| ENSRNOG00000001425 | Sh2b2 | | 1.041 | | 0.00005 | |
| ENSRNOG00000016535 | Ccl22 | | 1.040 | | 0.02625 | |
| ENSRNOG00000060010 | Ss18l1 | | 1.037 | | 0.00025 | |
| ENSRNOG00000043445 | Them5 | | 1.036 | | 0.00005 | |
| ENSRNOG00000023968 | Fancf | | 1.036 | | 0.00005 | |
| ENSRNOG00000014723 | Cbfa2t3 | | 1.036 | | 0.00005 | |
| ENSRNOG00000010311 | Dzip1 | | 1.035 | | 0.01885 | |
| ENSRNOG00000007078 | Wisp1 | | 1.035 | | 0.0008 | |
| ENSRNOG00000043225 | Zfp771 | | 1.035 | | 0.00005 | |
| ENSRNOG00000013653 | Pdlim7 | | 1.035 | | 0.00005 | |
| ENSRNOG00000048297 | LOC108348175 | | 1.035 | | 0.00045 | |
| ENSRNOG00000027008 | Igtp | | 1.035 | | 0.00005 | |
| ENSRNOG00000031475 | Col16a1 | | 1.034 | | 0.00005 | |
| ENSRNOG00000030187 | Mmp12 | | 1.034 | | 0.00005 | |
| ENSRNOG00000033262 | Reep6 | | 1.034 | | 0.00005 | |
| ENSRNOG00000018867 | Klhdc7a | | 1.033 | | 0.00005 | |
| ENSRNOG00000050312 | Ism2 | | 1.033 | | 0.0056 | |
| ENSRNOG00000028650 | Inf2 | | 1.033 | | 0.00005 | |
| ENSRNOG00000013694 | Ntng2 | | 1.033 | | 0.00005 | |
| ENSRNOG00000020164 | Ifitm10 | | 1.032 | | 0.00005 | |
| ENSRNOG00000055745 | AABR07043748.2 | | 1.031 | | 0.00005 | |
| ENSRNOG00000030721 | Fbrsl1 | | 1.031 | | 0.00005 | |
| ENSRNOG00000013046 | Tram2 | | 1.029 | | 0.00005 | |
| ENSRNOG00000016058 | Kazald1 | | 1.028 | | 0.00005 | |
| ENSRNOG00000060896 | AABR07063424.1 | | 1.028 | | 0.03635 | |
| ENSRNOG00000054709 | AABR07061382.2 | | 1.027 | | 0.0002 | |
| ENSRNOG00000004444 | Ikzf1 | | 1.026 | | 0.00005 | |
| ENSRNOG00000050819 | Birc5 | | 1.025 | | 0.0025 | |
| ENSRNOG00000036833 | Zfp385a | | 1.022 | | 0.00005 | |
| ENSRNOG00000057451 | Itga5 | | 1.021 | | 0.00005 | |
| ENSRNOG00000018371 | Tubb6 | | 1.020 | | 0.00005 | |
| ENSRNOG00000029707 | Mt-nd4 | | 1.020 | | 0.0259 | |
| ENSRNOG00000016695 | Mmp2 | | 1.019 | | 0.00005 | |
| ENSRNOG00000012830 | Paqr8 | | 1.019 | | 0.00005 | |
| ENSRNOG00000018358 | Nt5dc2 | | 1.018 | | 0.00005 | |
| ENSRNOG00000046382 | LOC100911365 | | 1.018 | | 0.00005 | |
| ENSRNOG00000037327 | F8a1 | | 1.018 | | 0.00005 | |
| ENSRNOG00000000695 | Ssh1 | | 1.016 | | 0.00005 | |
| ENSRNOG00000019382 | Zbtb47 | | 1.015 | | 0.00005 | |
| ENSRNOG00000028415 | Cdc20 | | 1.015 | | 0.00005 | |
| ENSRNOG00000006365 | Asb15 | | 1.014 | | 0.00005 | |
| ENSRNOG00000049270 | Ppp2r3b | | 1.014 | | 0.00005 | |
| ENSRNOG00000011967 | Nphp4 | | 1.014 | | 0.00005 | |
| ENSRNOG00000020587 | Efemp2 | | 1.013 | | 0.00005 | |
| ENSRNOG00000002659 | Ciita | | 1.013 | | 0.00005 | |
| ENSRNOG00000055042 | Gprin2 | | 1.013 | | 0.011 | |
| ENSRNOG00000004168 | Slc35e4 | | 1.012 | | 0.00005 | |
| ENSRNOG00000019440 | Kcnn4 | | 1.012 | | 0.00005 | |
| ENSRNOG00000051507 | Azin2 | | 1.011 | | 0.00185 | |
| ENSRNOG00000017857 | LOC108348250 | | 1.010 | | 0.00005 | |
| ENSRNOG00000054890 | Flna | | 1.009 | | 0.00005 | |
| ENSRNOG00000021084 | AABR07006310.1 | | 1.009 | | 0.00005 | |
| ENSRNOG00000007090 | Cacna1c | | 1.009 | | 0.00005 | |
| ENSRNOG00000014125 | Evi2b | | 1.008 | | 0.00195 | |
| ENSRNOG00000033787 | Adamtsl5 | | 1.008 | | 0.00005 | |
| ENSRNOG00000025261 | LOC691254 | | 1.007 | | 0.0176 | |
| ENSRNOG00000001868 | LOC100911248 | | 1.007 | | 0.00005 | |
| ENSRNOG00000003897 | Col1a1 | | 1.006 | | 0.0057 | |
| ENSRNOG00000019181 | Synpo | | 1.004 | | 0.00005 | |
| ENSRNOG00000016258 | Zfp516 | | 1.003 | | 0.00005 | |
| ENSRNOG00000010217 | Prrc2b | | 1.003 | | 0.00005 | |
| ENSRNOG00000015406 | Pgm5 | | 1.003 | | 0.00005 | |
| ENSRNOG00000027722 | H1fx | | 1.002 | | 0.00005 | |
| ENSRNOG00000000054 | Abhd8 | | 1.002 | | 0.00005 | |
| ENSRNOG00000006384 | Ddx58 | | 1.001 | | 0.00005 | |
| ENSRNOG00000006916 | Sardh | | 1.001 | | 0.00005 | |

Supplementary Table 8. List of significantly downregulated genes in the CYP-induced model compared to the control group

| Gene id | Gene name | Log_2_(Fold Change) | p_Value |
| --- | --- | --- | --- |
| ENSRNOG00000035103 | 5S_rRNA | -23.188 | 0.00135 |
| ENSRNOG00000054722 | U1 | -19.970 | 0.02285 |
| ENSRNOG00000048073 | LOC103690821 | -19.868 | 0.00135 |
| ENSRNOG00000055311 | U1 | -19.700 | 0.0077 |
| ENSRNOG00000053409 | AABR07068852.1 | -18.222 | 0.0036 |
| ENSRNOG00000052706 | AABR07072841.1 | -18.132 | 0.0058 |
| ENSRNOG00000050226 | RGD1565894 | -17.224 | 0.0001 |
| ENSRNOG00000028803 | Cd209e | -17.027 | 0.00005 |
| ENSRNOG00000061507 | AABR07032328.1 | -16.982 | 0.02285 |
| ENSRNOG00000046991 | RGD1565472 | -16.861 | 0.00005 |
| ENSRNOG00000054929 | AABR07072414.1 | -16.798 | 0.02285 |
| ENSRNOG00000057989 | Zp2 | -16.743 | 0.00005 |
| ENSRNOG00000057266 | Rnase1 | -16.327 | 0.0001 |
| ENSRNOG00000059962 | AABR07025787.1 | -16.152 | 0.00135 |
| ENSRNOG00000047914 | Gng11 | -4.815 | 0.04585 |
| ENSRNOG00000061740 | Smarcb1 | -4.302 | 0.00005 |
| ENSRNOG00000057515 | Arf3 | -4.233 | 0.00265 |
| ENSRNOG00000053109 | Mrpl53 | -4.049 | 0.00265 |
| ENSRNOG00000020625 | Mcpt2 | -4.008 | 0.00005 |
| ENSRNOG00000031792 | Mcpt1l4 | -3.815 | 0.04275 |
| ENSRNOG00000047187 | Surf2 | -3.672 | 0.0305 |
| ENSRNOG00000049964 | LOC103690044 | -3.533 | 0.04675 |
| ENSRNOG00000045790 | LOC100910130 | -3.449 | 0.00005 |
| ENSRNOG00000013953 | Ntrk1 | -3.410 | 0.00155 |
| ENSRNOG00000008873 | Ino80b | -3.409 | 0.00005 |
| ENSRNOG00000013141 | Eno2 | -3.318 | 0.00005 |
| ENSRNOG00000047642 | AABR07051426.1 | -3.269 | 0.0053 |
| ENSRNOG00000005059 | Med22 | -3.240 | 0.0057 |
| ENSRNOG00000049096 | Mcpt8l2 | -3.097 | 0.0013 |
| ENSRNOG00000052899 | AABR07049886.2 | -2.882 | 0.021 |
| ENSRNOG00000003136 | Fcrla | -2.870 | 0.00005 |
| ENSRNOG00000010640 | Agtr1b | -2.835 | 0.0076 |
| ENSRNOG00000046548 | Rhox5 | -2.810 | 0.00005 |
| ENSRNOG00000014505 | Pmfbp1 | -2.685 | 0.00005 |
| ENSRNOG00000054531 | AABR07016578.2 | -2.660 | 0.00925 |
| ENSRNOG00000059883 | LOC100912578 | -2.576 | 0.00005 |
| ENSRNOG00000046068 | AABR07027088.1 | -2.506 | 0.0459 |
| ENSRNOG00000053147 | AC141521.1 | -2.495 | 0.0001 |
| ENSRNOG00000005180 | Vstm2a | -2.411 | 0.0281 |
| ENSRNOG00000062158 | Rn60_1_2212.4 | -2.385 | 0.00005 |
| ENSRNOG00000019848 | Rbm39 | -2.285 | 0.00005 |
| ENSRNOG00000046280 | Tceal5 | -2.267 | 0.04645 |
| ENSRNOG00000054855 | AABR07014855.1 | -2.265 | 0.0182 |
| ENSRNOG00000023622 | LOC100910021 | -2.242 | 0.0069 |
| ENSRNOG00000028310 | S100vp | -2.239 | 0.00175 |
| ENSRNOG00000028982 | Mrgprb3 | -2.237 | 0.00005 |
| ENSRNOG00000050877 | Cetn3 | -2.200 | 0.0022 |
| ENSRNOG00000026672 | MGC94199 | -2.173 | 0.00005 |
| ENSRNOG00000012430 | Cxcr5 | -2.149 | 0.0287 |
| ENSRNOG00000053404 | NEWGENE_1306267 | -2.120 | 0.03165 |
| ENSRNOG00000000582 | Ddo | -2.115 | 0.00245 |
| ENSRNOG00000020993 | Ms4a2 | -2.093 | 0.00005 |
| ENSRNOG00000010199 | Glrb | -2.008 | 0.00005 |
| ENSRNOG00000024120 | Rxfp1 | -1.996 | 0.00005 |
| ENSRNOG00000052664 | Tnks2 | -1.955 | 0.00005 |
| ENSRNOG00000002723 | Sele | -1.933 | 0.00005 |
| ENSRNOG00000011892 | Slc36a2 | -1.929 | 0.00025 |
| ENSRNOG00000045747 | Capns1 | -1.927 | 0.00005 |
| ENSRNOG00000057677 | Rpl30l1 | -1.916 | 0.0388 |
| ENSRNOG00000062153 | LOC103690026 | -1.909 | 0.0251 |
| ENSRNOG00000015768 | Nat8f5 | -1.904 | 0.0166 |
| ENSRNOG00000010268 | Vom2r44 | -1.875 | 0.00005 |
| ENSRNOG00000051672 | AABR07008030.1 | -1.854 | 0.01775 |
| ENSRNOG00000029408 | Mageb16 | -1.849 | 0.00005 |
| ENSRNOG00000025957 | Ooep | -1.841 | 0.00575 |
| ENSRNOG00000043026 | Rps15-ps2 | -1.838 | 0.04475 |
| ENSRNOG00000015498 | Il17rb | -1.809 | 0.0003 |
| ENSRNOG00000038106 | Map1lc3b2 | -1.805 | 0.0212 |
| ENSRNOG00000022946 | Slc22a3 | -1.784 | 0.00005 |
| ENSRNOG00000032300 | Rpl39l | -1.753 | 0.0441 |
| ENSRNOG00000032350 | Kcnip4 | -1.741 | 0.01785 |
| ENSRNOG00000017072 | Slc16a14 | -1.726 | 0.00005 |
| ENSRNOG00000013312 | Kcnt2 | -1.703 | 0.00015 |
| ENSRNOG00000012714 | RGD1564149 | -1.656 | 0.00005 |
| ENSRNOG00000000195 | Vwa5a | -1.652 | 0.00005 |
| ENSRNOG00000037984 | Sccpdh | -1.622 | 0.00005 |
| ENSRNOG00000011623 | Rab3c | -1.615 | 0.00005 |
| ENSRNOG00000011181 | Cpa3 | -1.610 | 0.00005 |
| ENSRNOG00000002331 | Aldh3a1 | -1.609 | 0.00005 |
| ENSRNOG00000023386 | LOC103690018 | -1.597 | 0.00005 |
| ENSRNOG00000005023 | Agr2 | -1.587 | 0.00005 |
| ENSRNOG00000011400 | Ralyl | -1.584 | 0.00065 |
| ENSRNOG00000042795 | RGD1564571 | -1.568 | 0.00485 |
| ENSRNOG00000042524 | Milr1 | -1.564 | 0.0067 |
| ENSRNOG00000018505 | Cidea | -1.563 | 0.00835 |
| ENSRNOG00000031716 | Ecm2 | -1.555 | 0.00005 |
| ENSRNOG00000034191 | Fmo1 | -1.548 | 0.00005 |
| ENSRNOG00000049229 | LOC100911238 | -1.539 | 0.00005 |
| ENSRNOG00000048760 | AABR07047219.1 | -1.534 | 0.03945 |
| ENSRNOG00000014518 | Adam28 | -1.524 | 0.00005 |
| ENSRNOG00000023182 | RGD1565317 | -1.508 | 0.0091 |
| ENSRNOG00000059964 | AABR07061825.1 | -1.501 | 0.04045 |
| ENSRNOG00000010663 | Col6a5 | -1.500 | 0.00005 |
| ENSRNOG00000019342 | Sult1a1 | -1.496 | 0.00005 |
| ENSRNOG00000036802 | Snhg11 | -1.495 | 0.00005 |
| ENSRNOG00000003221 | Myoc | -1.488 | 0.00005 |
| ENSRNOG00000011250 | Inmt | -1.484 | 0.00005 |
| ENSRNOG00000060602 | AABR07014275.1 | -1.483 | 0.00005 |
| ENSRNOG00000052977 | LOC103690033 | -1.481 | 0.0001 |
| ENSRNOG00000021735 | Akr1cl | -1.449 | 0.00005 |
| ENSRNOG00000005792 | Ankef1 | -1.445 | 0.00055 |
| ENSRNOG00000043100 | Rn50_18_0532.1 | -1.443 | 0.0228 |
| ENSRNOG00000018839 | Ntrk2 | -1.432 | 0.00005 |
| ENSRNOG00000004994 | Agr3 | -1.422 | 0.00015 |
| ENSRNOG00000001893 | LOC100362453 | -1.419 | 0.00005 |
| ENSRNOG00000013282 | Mctp1 | -1.408 | 0.00005 |
| ENSRNOG00000002031 | Naa11 | -1.405 | 0.00005 |
| ENSRNOG00000010655 | Mttp | -1.404 | 0.00005 |
| ENSRNOG00000048088 | Mest | -1.399 | 0.00005 |
| ENSRNOG00000029861 | Gsta2 | -1.394 | 0.01105 |
| ENSRNOG00000009177 | Fcer1a | -1.378 | 0.0009 |
| ENSRNOG00000053494 | Mcpt1l1 | -1.369 | 0.00005 |
| ENSRNOG00000030788 | Olr36 | -1.355 | 0.00115 |
| ENSRNOG00000015519 | Ces1d | -1.353 | 0.00005 |
| ENSRNOG00000042628 | RGD1561145 | -1.335 | 0.0002 |
| ENSRNOG00000051858 | AC120727.1 | -1.331 | 0.00005 |
| ENSRNOG00000030106 | RGD1564698 | -1.331 | 0.00005 |
| ENSRNOG00000050146 | LOC100909732 | -1.322 | 0.0007 |
| ENSRNOG00000045797 | Lep | -1.315 | 0.00005 |
| ENSRNOG00000052909 | AABR07036746.1 | -1.313 | 0.00005 |
| ENSRNOG00000048597 | LOC100910732 | -1.308 | 0.00005 |
| ENSRNOG00000047647 | Il2ra | -1.297 | 0.0001 |
| ENSRNOG00000011484 | LOC680700 | -1.294 | 0.03455 |
| ENSRNOG00000051627 | AABR07053580.1 | -1.289 | 0.01315 |
| ENSRNOG00000046120 | RGD1561252 | -1.286 | 0.01295 |
| ENSRNOG00000042317 | Cd209f | -1.285 | 0.0026 |
| ENSRNOG00000021207 | Lgals12 | -1.276 | 0.00005 |
| ENSRNOG00000046950 | Ewsr1 | -1.269 | 0.00005 |
| ENSRNOG00000011672 | Tph1 | -1.269 | 0.00005 |
| ENSRNOG00000059776 | Tnks2 | -1.266 | 0.00005 |
| ENSRNOG00000018131 | Slc16a4 | -1.264 | 0.0011 |
| ENSRNOG00000002158 | Ibsp | -1.263 | 0.00025 |
| ENSRNOG00000018445 | Agt | -1.263 | 0.00005 |
| ENSRNOG00000015428 | Mff | -1.256 | 0.00005 |
| ENSRNOG00000009530 | Uchl3 | -1.247 | 0.00005 |
| ENSRNOG00000050492 | LOC100911807 | -1.237 | 0.0108 |
| ENSRNOG00000011705 | Stmn2 | -1.234 | 0.00005 |
| ENSRNOG00000061215 | Crym | -1.232 | 0.0163 |
| ENSRNOG00000056493 | Mybpc1 | -1.230 | 0.00005 |
| ENSRNOG00000047365 | Fau | -1.226 | 0.0057 |
| ENSRNOG00000011648 | Aqp1 | -1.222 | 0.00005 |
| ENSRNOG00000001821 | Adipoq | -1.220 | 0.00005 |
| ENSRNOG00000008282 | Elf5 | -1.216 | 0.00075 |
| ENSRNOG00000000201 | Gsta5 | -1.214 | 0.00005 |
| ENSRNOG00000019751 | Cyb5r2 | -1.211 | 0.0007 |
| ENSRNOG00000061096 | Rn7sl1 | -1.210 | 0.00005 |
| ENSRNOG00000042727 | RGD1563159 | -1.208 | 0.02595 |
| ENSRNOG00000021015 | Sbsn | -1.201 | 0.00005 |
| ENSRNOG00000026112 | Tmem202 | -1.198 | 0.0238 |
| ENSRNOG00000008720 | LOC103692173 | -1.193 | 0.00005 |
| ENSRNOG00000040108 | Cd36 | -1.191 | 0.00005 |
| ENSRNOG00000021831 | Kcnrg | -1.189 | 0.04625 |
| ENSRNOG00000026979 | RGD1561444 | -1.188 | 0.0068 |
| ENSRNOG00000016961 | Rps27 | -1.182 | 0.00005 |
| ENSRNOG00000005718 | AABR07065406.1 | -1.180 | 0.0004 |
| ENSRNOG00000000386 | Pbld1 | -1.178 | 0.00005 |
| ENSRNOG00000049878 | Sytl2 | -1.178 | 0.0005 |
| ENSRNOG00000010063 | Htr2a | -1.176 | 0.00005 |
| ENSRNOG00000020212 | Prr35 | -1.162 | 0.00005 |
| ENSRNOG00000023320 | Tspan1 | -1.161 | 0.0001 |
| ENSRNOG00000030449 | Gsta4 | -1.152 | 0.00005 |
| ENSRNOG00000049743 | NEWGENE_620381 | -1.150 | 0.00405 |
| ENSRNOG00000046848 | PCOLCE2 | -1.146 | 0.00005 |
| ENSRNOG00000049814 | LOC100910882 | -1.145 | 0.00135 |
| ENSRNOG00000046832 | Rn50_X_0745.4 | -1.135 | 0.00005 |
| ENSRNOG00000015562 | Cdh17 | -1.134 | 0.00005 |
| ENSRNOG00000056863 | RGD1562725 | -1.132 | 0.02175 |
| ENSRNOG00000014149 | Npy1r | -1.124 | 0.00035 |
| ENSRNOG00000045686 | Nfs1 | -1.116 | 0.00015 |
| ENSRNOG00000012772 | Nqo1 | -1.112 | 0.00005 |
| ENSRNOG00000005017 | RGD1566369 | -1.110 | 0.00005 |
| ENSRNOG00000030069 | Faim | -1.107 | 0.0001 |
| ENSRNOG00000005826 | RGD1562420 | -1.106 | 0.02005 |
| ENSRNOG00000046758 | Tmem217 | -1.106 | 0.019 |
| ENSRNOG00000056249 | LOC108348568 | -1.093 | 0.0065 |
| ENSRNOG00000020563 | Cma1 | -1.092 | 0.00005 |
| ENSRNOG00000019776 | Sh3gl3 | -1.091 | 0.0001 |
| ENSRNOG00000059545 | AC114446.1 | -1.089 | 0.01195 |
| ENSRNOG00000003476 | Slc6a4 | -1.083 | 0.00005 |
| ENSRNOG00000005853 | Tacr1 | -1.080 | 0.00005 |
| ENSRNOG00000010345 | Ssmem1 | -1.080 | 0.03185 |
| ENSRNOG00000058206 | AABR07058788.4 | -1.072 | 0.01825 |
| ENSRNOG00000059069 | LOC100911337 | -1.066 | 0.00005 |
| ENSRNOG00000020644 | Nsg2 | -1.062 | 0.00005 |
| ENSRNOG00000032765 | RGD1559955 | -1.060 | 0.00015 |
| ENSRNOG00000016613 | Hoxc4 | -1.058 | 0.0034 |
| ENSRNOG00000004275 | Egfl6 | -1.042 | 0.00005 |
| ENSRNOG00000050181 | Chid1 | -1.035 | 0.00005 |
| ENSRNOG00000009686 | Aqp7 | -1.033 | 0.00385 |
| ENSRNOG00000039025 | AABR07051947.1 | -1.027 | 0.02345 |
| ENSRNOG00000055451 | Gcnt4 | -1.027 | 0.01745 |
| ENSRNOG00000057433 | AABR07027581.1 | -1.026 | 0.0052 |
| ENSRNOG00000026661 | Hcar1 | -1.017 | 0.00335 |
| ENSRNOG00000031591 | AABR07032255.2 | -1.013 | 0.0133 |
| ENSRNOG00000023664 | Lepr | -1.010 | 0.00645 |
| ENSRNOG00000007258 | Sbspon | -1.010 | 0.00005 |
| ENSRNOG00000009291 | Dnase1l3 | -1.009 | 0.00005 |
| ENSRNOG00000051303 | Rn50_13_0839.5 | -1.007 | 0.00005 |
| ENSRNOG00000003135 | Fcrlb | -1.001 | 0.0168 |
